# Evolutionary conservation and changes in insect TRP channels

**DOI:** 10.1186/1471-2148-9-228

**Published:** 2009-09-10

**Authors:** Hironori Matsuura, Takaaki Sokabe, Keigo Kohno, Makoto Tominaga, Tatsuhiko Kadowaki

**Affiliations:** 1 Graduate School of Bioagricultural Sciences, Nagoya University, Chikusa, Nagoya 464-8601, Japan; 2 Section of Cell Signaling, Okazaki Institute for Integrative Bioscience (National Institute for Physiological Sciences), National Institutes of Natural Sciences, Okazaki 444-8787, Japan; 3 Department of Physiological Sciences, The Graduate University for Advanced Studies, Okazaki 444-8585, Japan

## Abstract

**Background:**

TRP (Transient Receptor Potential) channels respond to diverse stimuli and thus function as the primary integrators of varied sensory information. They are also activated by various compounds and secondary messengers to mediate cell-cell interactions as well as to detect changes in the local environment. Their physiological roles have been primarily characterized only in mice and fruit flies, and evolutionary studies are limited. To understand the evolution of insect TRP channels and the mechanisms of integrating sensory inputs in insects, we have identified and compared TRP channel genes in *Drosophila melanogaster, Bombyx mori, Tribolium castaneum, Apis mellifera, Nasonia vitripennis*, and *Pediculus humanus *genomes as part of genome sequencing efforts.

**Results:**

All the insects examined have 2 TRPV, 1 TRPN, 1 TRPM, 3 TRPC, and 1 TRPML subfamily members, demonstrating that these channels have the ancient origins in insects. The common pattern also suggests that the mechanisms for detecting mechanical and visual stimuli and maintaining lysosomal functions may be evolutionarily well conserved in insects. However, a TRPP channel, the most ancient TRP channel, is missing in *B. mori*, *A. mellifera*, and *N. vitripennis*. Although *P. humanus *and *D. melanogaster *contain 4 TRPA subfamily members, the other insects have 5 TRPA subfamily members. *T. castaneum*, *A. mellifera*, and *N. vitripennis *contain TRPA5 channels, which have been specifically retained or gained in Coleoptera and Hymenoptera. Furthermore, TRPA1, which functions for thermotaxis in *Drosophila*, is missing in *A. mellifera *and *N. vitripennis*; however, they have other Hymenoptera-specific TRPA channels (AmHsTRPA and NvHsTRPA). NvHsTRPA expressed in HEK293 cells is activated by temperature increase, demonstrating that HsTRPAs function as novel thermal sensors in Hymenoptera.

**Conclusion:**

The total number of insect TRP family members is 13-14, approximately half that of mammalian TRP family members. As shown for mammalian TRP channels, this may suggest that single TRP channels are responsible for integrating diverse sensory inputs to maintain the insect sensory systems. The above results demonstrate that there are both evolutionary conservation and changes in insect TRP channels. In particular, the evolutionary processes have been accelerated in the TRPA subfamily, indicating divergence in the mechanisms that insects use to detect environmental temperatures.

## Background

Transient receptor potential (TRP) superfamily members of cation channels share six common transmembrane domains and permeability to cations. Despite these similarities, TRP channels are highly unusual among the known families of ion channels in displaying an impressive diversity of cation selectivities and specific activation mechanisms. TRP channels have crucial functions for various sensory modalities in different metazoans. They are involved in vision, thermosensation, olfaction, hearing, and mechanosensation and thus enable animals to perceive the external environment. Moreover, TRP channels enable individual cells to sense changes in their local environment, for example, osmolarity and fluid flow. Many TRP channels are activated by a variety of different stimuli and function as primary signal integrators. TRP channels are expressed and function in a variety of multicellular organisms, including nematodes, fruit flies, fish, mice, and humans. The TRP superfamily is divided into seven subfamilies, namely, TRPC, TRPA, TRPV, TRPN, TRPM, TRPP, and TRPML based on their sequence elements and domains. For example, TRPC, TRPA, TRPV, and TRPN channels have multiple N-terminal ankyrin repeats [1-3].

Physiological functions of TRP channels have been exclusively characterized in fruit flies and mice to date. Moreover, comparative genomics and evolutionary studies of TRP channels are also limited. We therefore performed a comparative genomics study of insect TRP channels as part of insect genome sequencing efforts. The sensory modalities of insects are almost comparable to those of mammals; insects can see, feel touch, hear, smell, taste, and detect temperature, humidity, wind, gravity, magnetism, and seismogram. It has been shown that some of these sensory modalities are dependent on TRP channels in fruit flies [4-10]. Among insect sensory modalities, the mechanisms of thermosensation could be quite different from those of mammals because insects are relatively small, and thus they have a larger ratio of surface area to volume than mammals. Furthermore, they do not have systems to keep their body temperature constant. Thus, their body temperatures are more susceptible to changes in environmental temperature than mammals. While mammals sense the temperatures of body surface and directly touched objects as somatosensory stimuli, insects are able to sense temperatures from the distance by detecting the temperature gradient of air [11,12]. This might lead to the different evolution of thermo-sensitive TRP channel genes in insects and mammals. Thermo-sensitive TRP channels are the specific TRP channels which are activated by either temperature increase or decrease [1-3]. Since all thermo-sensitive TRP channels belong to TRPA subfamily in fruit flies [8-10], the evolution of insect TRPA subfamily might be very dramatic. Here, we identify and compare all TRP channel genes in the sequenced insects, *Drosophila melanogaster *(Diptera), *Bombyx mori *(Lepidoptera), *Tribolium castaneum *(Coleoptera), *Apis mellifera *(Hymenoptera), *Nasonia vitripennis *(Hymenoptera), and *Pediculus humanus *(Phthiraptera). These insect species represent 4 major orders of holometabolous insects as well as 1 order of hemimetabolous insects. Evolutionary conservation and changes in the insect TRP channels are discussed.

## Results and Discussion

### Conservation of insect TRPV, TRPN, TRPM, and TRPML subfamilies

**Figure 1:**
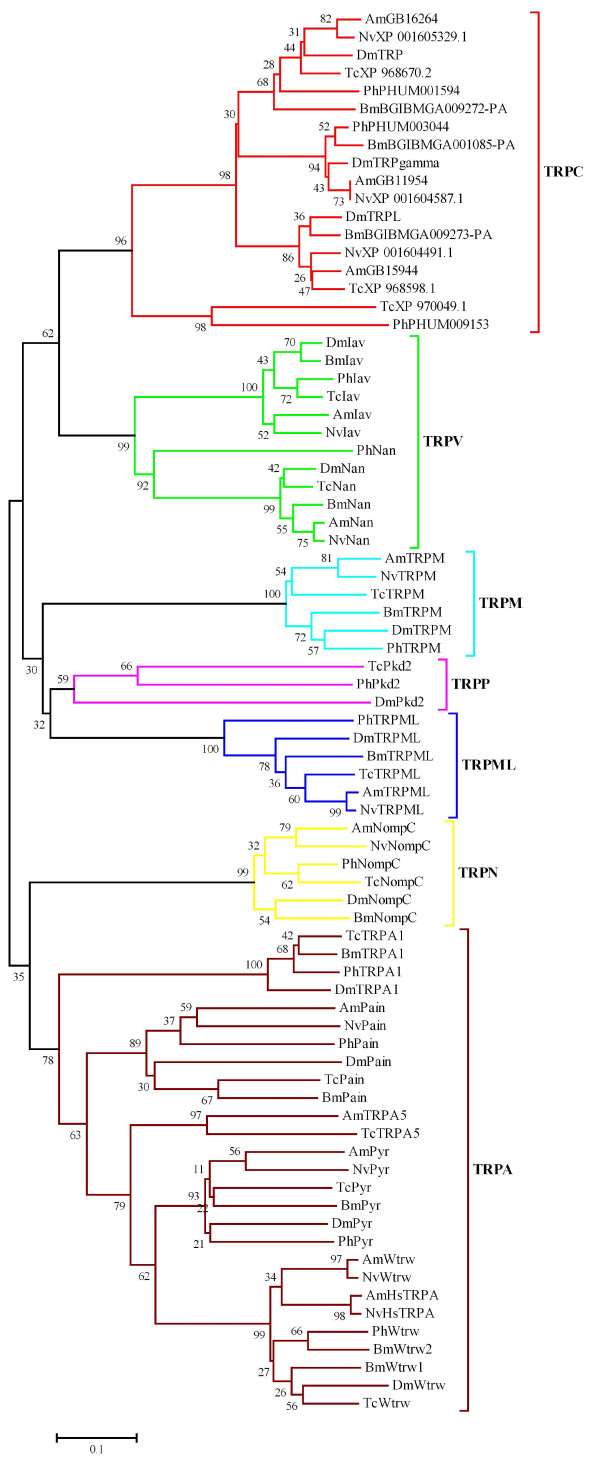
**Phylogenetic tree of the insect TRP channels**. Amino acid sequences of channel-forming six transmembrane domains of *Drosophila melanogaster *(Dm), *Bombyx mori *(Bm), *Tribolium castaneum *(Tc), *Apis mellifera *(Am), *Nasonia vitripennis *(Nv), and *Pediculus humanus *(Ph) TRP channels were aligned by the Muscle program, and then PhyML3.0 algorithm was applied for the maximum likelihood analyses, under the WAG amino acid substitution model and with 100 bootstrapped data sets using the PhyML Online server. The statistical confidence (bootstrap value) is indicated next to each interior branch. The insect TRP channels can be classified into 7 subfamilies, TRPC (red), TRPV (green), TRPM (light blue), TRPP (purple), TRPML (blue), TRPN (yellow), and TRPA (brown). The amino acid sequences of the insect TRP channels are listed in an Additional file 2.

**Figure 2:**
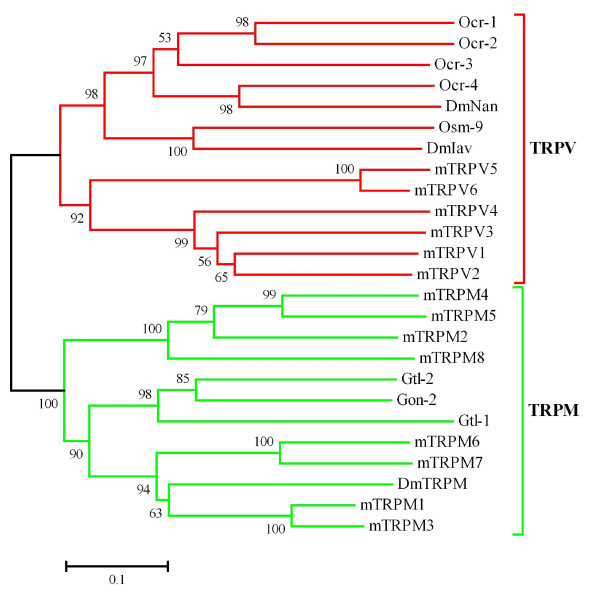
**Phylogenetic tree of *D. melanogaster*, *C. elegans*, and mouse TRPV and TRPM channels**. The phylogenetic tree was constructed from the amino acid sequences of channel-forming six transmembrane domains of *D. melanogaster *TRPV (DmNan and DmIav), TRPM (DmTRPM), *C. elegans *TRPV (Ocr-1, 2, 3, 4, and Osm-9), TRPM (Gtl-1, 2, and Gon-2), and mouse TRPV (mTRPV1, 2, 3, 4, 5, and 6), TRPM (mTRPM1, 2, 3, 4, 5, 6, 7, and 8) as in Fig. 1. TRPV and TRPM subfamily members are shown by red and green, respectively. Nematode and mouse TRPV and TRPM channels form independent clades, demonstrating that nematode and mouse have independently expanded these TRP channels.

There are 13 identified TRP channel genes in *Drosophila *[1]; we used them as queries to identify TRP channel genes in the *P. humanus, B. mori, T. castaneum, A. mellifera*, and *N. vitripennis *genomes. A summary of the results is shown in Table 1 and 2. Classification of the TRP channels into subfamilies is based on the phylogenetic tree (Fig. 1) constructed from the channel-forming six transmembrane domain sequences of all TRP channels identified. These sequences were chosen for the analysis because they are the only domains shared with all TRP channels, and the other parts are too divergent. All the insects examined contain 2 TRPV, 1 TRPN, 1 TRPM, 3 TRPC, and 1 TRPML channels. *Drosophila *TRPV channels (Nan and Iav) are necessary for hearing [6,7,13], and Nan functions as a dry receptor for hygrosensation [14]. The *Drosophila *TRPN channel, NompC, is associated with mechanosensation [4] as well as hearing [13,15]. *Drosophila *TRPC channels, TRP and TRPL, are necessary for phototransduction [16,17]. TRPML is necessary for lysosomal function and autophagy in *Drosophila *[18]. The functions of the TRPM channel have not been identified in insects. Some of mammalian TRPM channels are associated with taste [19] and cold perception [20,21]. These results may suggest that the mechanisms to detect mechanical and visual stimuli as well as humidity are evolutionarily well conserved in both holometabolous and hemimetabolous insects. *Daphnia pulex*, a crustacean, contains the same numbers of members of these TRP subfamilies except that it has 2 TRPM channels (data not shown). These results thus suggest that 2 TRPV, 1 TRPN, 1 TRPM, 3 TRPC, and 1 TRPML channels were at least present in the last common ancestor of insects and branchiopods [22-24]. Furthermore, they could be the "core" set of TRP channels present in arthropods. The nematode *Caenorhabditis elegans *contains 5 TRPV, 1 TRPN, 4 TRPM, 3 TRPC, and 1 TRPML channels, more TRPV and TRPM channels than are present in insects [25]. Similarly, the vertebrates, for example mouse, have 6 TRPV and 8 TRPM channels. Phylogenetic analysis of *C. elegans*, *D. melanogaster*, and mouse TRPV and TRPM channels demonstrates that *C. elegans *and mouse independently expanded these subfamily members during their evolution (Fig. 2). It is therefore unlikely that insects have lost them from the last common ancestor of Bilateria (Urbilateria). The origin of the TRPN channel can be traced back to Urbilateria because it is present in both Ecdysozoans (insects, water flea, and nematode) and zebrafish [26], and has been specifically lost in mammals.

The total number of insect TRP family members is 13-14, approximately half that of mammalian TRP family members. Nevertheless, insects have highly developed sensory systems almost comparable to mammals, suggesting that some single TRP channels are responsible for detecting multiple sensory stimuli in insects as reported for mammalian TRP channels. This is also supported by the facts that Nan, the *Drosophila *TRPV channel, is essential for both hearing [6] and hygrosensation [14], and DmTRP and DmTRPL, *Drosophila *TRPC channels, function in vision [16,17] and possibly thermosensation [27].

**Table 1:** The number of TRP subfamily members in *P. humanus, D. melanogaster, B. mori, T. castaneum, A. mellifera*, and *N. vitripennis*

	**TRPV**	**TRPN**	**TRPM**	**TRPC**	**TRPML**	**TRPP**	**TRPA**
***P. humanus***	2	1	1	3	1	1	4

***D. melanogaster***	2	1	1	3	1	1	4

***B. mori***	2	1	1	3	1	0	5

***T. castaneum***	2	1	1	3	1	1	5

***A. mellifera***	2	1	1	3	1	0	5

***N. vitripennis***	2	1	1	3	1	0	5

**Table 2:** The number of TRPA subfamily members in *P. humanus, D. melanogaster, B. mori, T. castaneum, A. mellifera*, and *N. vitripennis*

	**TRPA1**	**Pain**	**Pyr**	**Wtrw**	**TRPA5**	**HsTRPA**
***P. humanus***	1	1	1	1	0	0

***D. melanogaster***	1	1	1	1	0	0

***B. mori***	1	1	1	2	0	0

***T. castaneum***	1	1	1	1	1	0

***A. mellifera***	0	1	1	1	1	1

***N. vitripennis***	0	1	1	1	1	1

### TRPP subfamily is missing in *Bombyx*, *Apis*, and *Nasonia*

Interestingly, a TRPP channel is absent in *Bombyx*, *Apis *and *Nasonia *(Table 1). Because TRPP is present in yeast, it is thought to be the most ancient TRP subfamily [28]. This suggests that TRPP was present in Urbilateria, and has been lost in Lepidoptera and Hymenoptera. TRPP channels are present on both motile and primary cilia, and they may function to sense fluid flow, osmolarity, and mechanical stretch [2]. The *Drosophila *TRPP, Pkd2, is essential for sperm entry in the female storage organs [29,30], and is also involved in larval feeding behavior [31]. The lack of TRPP demonstrates that the functions of TRPP can be compensated by evolving an alternative pathway or other TRP subfamilies in Lepidoptera and Hymenoptera. Moreover, Fig. 1 shows that the evolutionary rate of TRPP channels in *Pediculus, Tribolium*, and *Drosophila *has been accelerated relative to other TRP subfamily members. Thus, TRPP channels could have different physiological functions in each insect species.

### Differences in insect TRPC subfamily members

The *Drosophila *TRPC subfamily members, DmTRP and DmTRPL, were the first and founding members of the whole TRP superfamily. They were originally identified in association with phototransduction [16,17]. It has been suggested that the primary channels functioning in phototransduction are DmTRP homomultimers and two types of heteromultimers--DmTRPL/DmTRP and DmTRPL/DmTRPgamma--based on the observation that these channels were activated by stimulation of signaling pathways coupled to activation of phosphoinositide (PI)-dependent phospholipase C (PLC) *in vitro *[32,33]. Nevertheless, DmTRP homomultimers should be the major components *in vivo *because *trpl *mutant flies show only subtle defect in the light response [1]. *Bombyx, Apis*, and *Nasonia *contain 3 TRPC subfamily members that cluster with either DmTRP, DmTRPL, or DmTRPgamma (Fig. 1). This suggests that TRPC subfamily members of *B. mori*, *A. mellifera*, *N. vitripennis*, and *D. melanogaster *share the same functions in phototransduction. However, *Tribolium *and *Pediculus *appear to lack the DmTRPgamma and DmTRPL orthologs, respectively. Instead, these insects contain TcXP970049.1 and PhPHUM009153 that cluster separately from the other TRPC subfamily members (Fig. 1). This suggests that both heteromultimers corresponding to DmTRPL/DmTRP and DmTRPL/DmTRPgamma and the heteromultimer corresponding to DmTRPL/DmTRPgamma may be absent in *P. humanus *and *T. castaneum*, respectively. Thus, TRPC subfamily members may have been co-opted to play physiological roles other than phototransduction in *P. humanus *and *T. castaneum*. In fact, the nematode has 3 TRPC channels similar to insects, and one of them, TRP-3, is expressed in the spermatids and functions for fertilization [34]. To address this question, it will be useful to examine where the TRPC subfamily members are expressed in *Tribolium *and *Pediculus*.

### Dynamic evolution of the insect TRPA subfamily

As shown in Table 2, 5 TRPA subfamily members are present in *Bombyx, Tribolium, Apis*, and *Nasonia*, while 4 members are present in *Pediculus *and *Drosophila*. This is in contrast to mammals which have only 1 TRPA channel. Interestingly, *Daphnia *and *Caenorhabditis *appear to contain only 1 and 2 TRPA channels, respectively. These results demonstrate that insects specifically expanded the TRPA subfamily members during their evolution after diverging from other metazoans.

The phylogeny of insect TRPA channels demonstrates that all insects examined contain TRPA channels orthologous to *Drosophila *Pain, Pyr, and Wtrw. *Drosophila *Pain and Pyr function as thermosensors responding to different "hot" temperatures (The temperature thresholds for activating Pain and Pyr are 42.6 and 37.5-40°C, respectively) [8,10,35], and *Drosophila *Wtrw functions as a moist receptor for hygrosensation [14]. These results suggest that the mechanisms to detect harmful high temperatures as well as moisture are conserved among different insect species.

**Figure 3:**
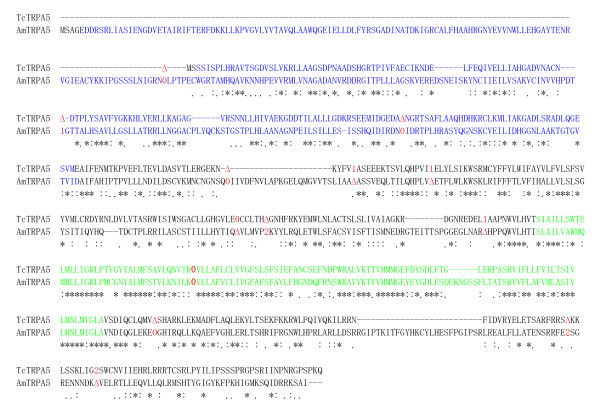
**Intron positions and phases of *TcTRPA5 *and *AmTRPA5***. Protein-level alignment of *TcTRPA5 *and *AmTRPA5 *is shown. Identical amino acids are indicated by asterisks, and the conserved amino acids are shown by either dots or colons. Ankyrin repeat and ion transport domains are shown by blue and green, respectively. Intron positions are indicated by digits corresponding to the phase of the intron relative to the surrounding codons (phases 0, 1 and 2 introns fall before the first, second and third bases of a codon, respectively) in red. Delta indicates the absence of an intron. Introns 1, 2, and 4 of *AmTRPA5 *are located in the alignment gap regions. One intron in the ion transport domain-coding region of both *TcTRPA5 *and *AmTRPA5 *shares the same position and phase (indicated by red bold letters).

*Tribolium, Apis*, and *Nasonia *(data not shown) have an extra TRPA channel, TRPA5, (Fig. 1). Based on the recent phylogeny of holometabolous insects [36,37], it is likely that the ancient *TRPA5 *was present in the common ancestor of holometabolous insects, and then has been lost in Lepidoptera and Diptera. To address whether *TcTRPA5 *and *AmTRPA5 *have the same origin, we compared the positions and phases of their introns [38]. We were not able to analyze *NvTRPA5 *since there is a sequence gap in the genomic region. *TcTRPA5 *and *AmTRPA5 *contain 6 and 8 introns, respectively; however, 3 introns of *AmTRPA5 *are located in the alignment gap sites (Fig. 3). Among them, one intron in the most conserved ion transport domain-coding region shares the same position and phase (Fig. 3). Intriguingly, this intron is absent in all other insect TRP genes at the corresponding sites. This suggests that the ancient *TRPA5 *was retained only in Coleoptera and Hymenoptera, and then intron gain and loss have occurred. Nevertheless, the possibility that Coleoptera and Hymenoptera have independently gained *TRPA5 *could not be completely ruled out. AmTRPA5 expressed in HEK293 cells cannot be activated by temperature fluctuations (data not shown); thus, it remains to be determined how the TRPA5 channel is activated.

**Figure 4:**
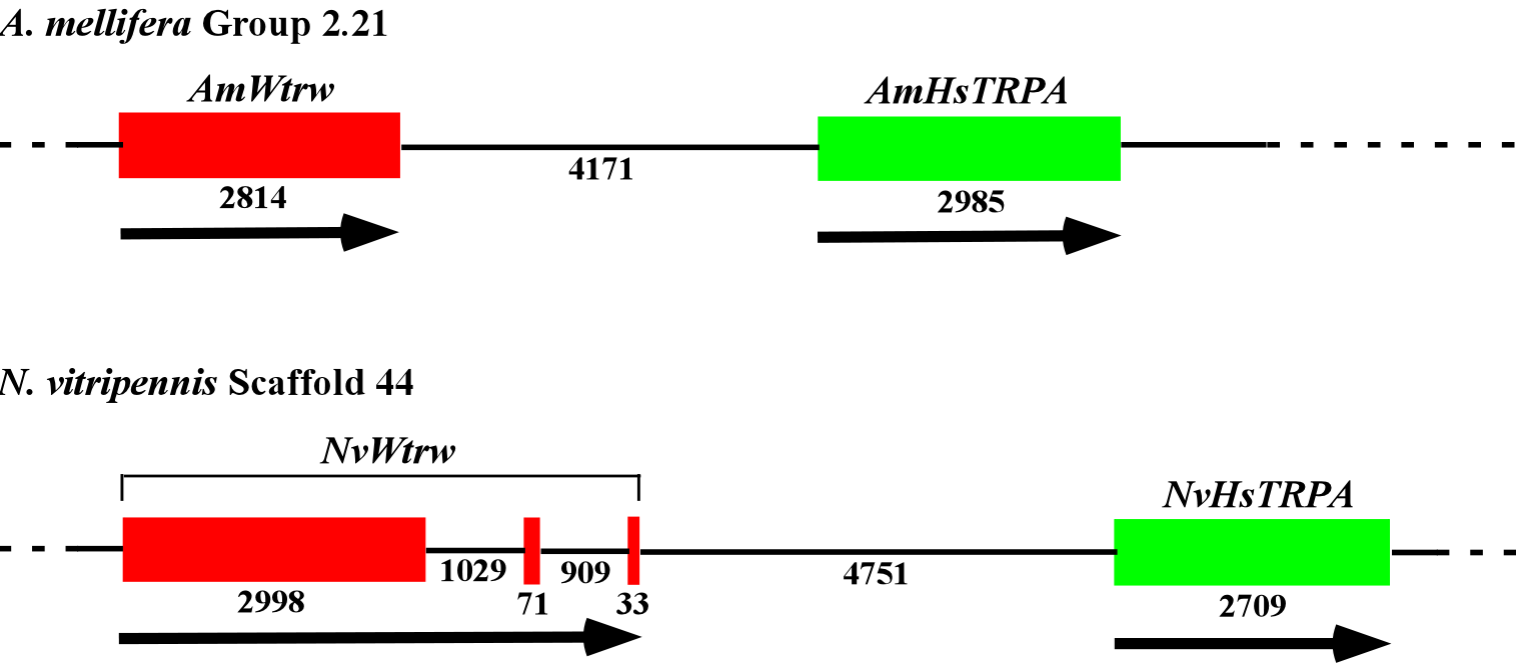
***HsTRPA *has evolved by duplication of the *Wtrw *gene**. Positions of *AmWtrw *and *AmHsTRPA *genes on *A. mellifera *Group 2.21 scaffold as well as *NvWtrw *and *NvHsTRPA *on *N. vitripennis *scaffold 44 are shown. The exons of *Wtrw *and *HsTRPA *genes are indicated by red and green rectangles, respectively. The numbers indicate the sizes of DNA (bp), and the arrows show the direction of transcription of genes. *AmWtrw*, *AmHsTRPA*, and *NvHsTRPA *have no introns, while *NvWtrw *has 2 introns. The genes adjacent to *Wtrw *and *HsTRPA *are also conserved between *Apis *and *Nasonia*.

*Apis *and *Nasonia *lack TRPA1, which functions in thermotactic behaviors of *Drosophila *larvae and adults [9,39,40] (Table 2 and Fig. 1). Moreover, DmTRPA1 was shown to be activated by temperature increase [39,41]. Remarkably, *Apis *and *Nasonia *have HsTRPAs (Hymenoptera specific TRPA) which cluster with Wtrw members (Fig. 1). We will show below that HsTRPA functions as a thermosensor to detect temperature increase. *HsTRPA *genes have evolved by duplication of *Wtrw *genes, since these two genes lie next to each other in *Apis *and *Nasonia *chromosomes (Fig. 4). Interestingly, 2 *Bombyx Wtrw *genes, encoding BmWtrw1, which forms a clade with DmWtrw and TcWtrw, and BmWtrw2, which forms a clade with PhWtrw, have also evolved by duplication since they are present in the same genomic scaffold (data not shown). Because it is unlikely that the duplication of *Wtrw *has occurred independently in Lepidoptera and Hymenoptera, this event must have occurred in the common ancestor of holometabolous insects, and the duplicated genes have been lost in Diptera and Coleoptera. Since DmWtrw was shown to be necessary for detecting moist air [14], it will be interesting to see how BmWtrw1 and BmWtrw2 channels are activated. *AmHsTRPA, NvHsTRPA, AmWtrw, BmWtrw1, BmWtrw2, TcWtrw*, and *PhWtrw *lack introns, while *NvWtrw *and *DmWtrw *have 2 and 3 introns, respectively. This suggests that the ancient *Wtrw *gene evolved by retrotransposition of another TRP channel gene, most likely *Pyr*, and then *Drosophila *and *Nasonia *have gained introns in the *Wtrw *genes. The lack of introns in *NvHsTRPA *suggests that it also has evolved by retrotransposition of *NvWtrw *in *Nasonia*. Apparently, the evolution of TRPA channels has been most dramatic among TRP subfamilies in insects, suggesting that the mechanisms to detect environmental temperature vary in different insect species.

### NvHsTRPA is a thermo-sensitive TRP channel

**Figure 5:**
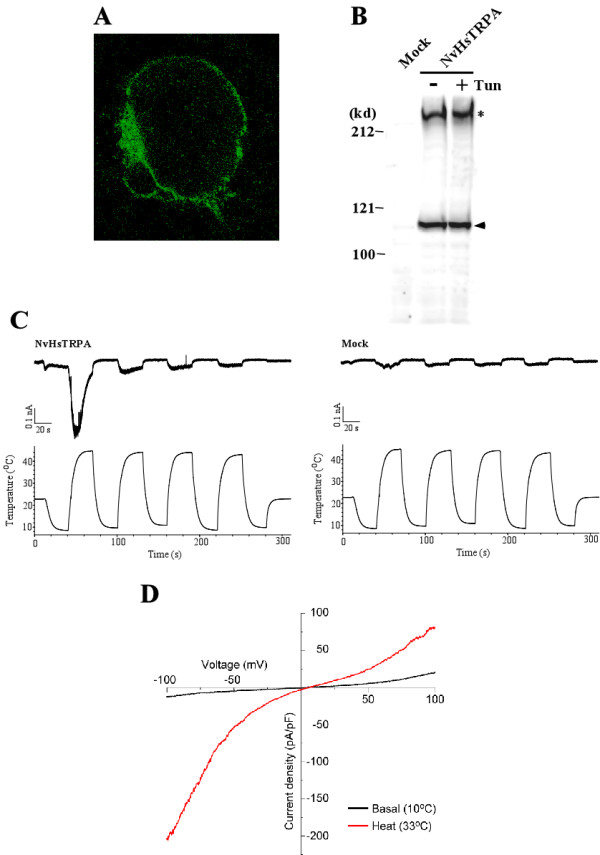
**NvHsTRPA is a thermo-sensitive TRP channel**. (A) Localization of V5 and His epitope-tagged NvHsTRPA protein in HEK293 cells visualized by immunofluorescence. (B) Detection of the epitope-tagged NvHsTRPA protein by Western blot. The lysates of HEK293 cells transfected with empty vector (Mock) and *NvHsTRPA *expression vector (NvHsTRPA) were analyzed by Western blot. Arrowhead indicates NvHsTRPA protein band, while asterisk shows the heat aggregated protein band. The *NvHsTRPA *expressing cells were treated with (+) or without (-) tunicamycin (Tun). The positions of MW markers are shown on the left in kDa. (C) Heat elicits inward current activation in a NvHsTRPA-expressing HEK293 cell (NvHsTRPA, left panel) at -60 mV holding potential in a whole-cell patch-clamp mode (*n *= 6). A standard bath solution and Cs-Asp/Ca^2+^(-) pipette solution were used. NvHsTRPA is activated by temperature increase, and quickly inactivated by constant heat application. Desensitization of the channel is also observed by repetitive heat applications. The current activation is not observed with the mock-transfected cells (Mock, right panel). (D) Current--voltage relationship of heat evoked current exhibits dual rectification with a slight positive reversal potential. Heat-dependent shifts of the liquid junction potentials (ΔJP_H_) were not corrected in the plot (*n *= 9). A standard bath solution and Cs-Asp/Ca^2+^(-) pipette solution were used.

To characterize the properties of the NvHsTRPA channel, we expressed it in HEK293 cells. Localization of the V5 and His epitope-tagged NvHsTRPA protein showed a staining pattern at the plasma membrane, demonstrating that it reaches to the cell surface (Fig. 5A). Nevertheless, some proteins are present in the cytoplasm as well. The molecular weight of the tagged protein was approximately 112 kDa, which is slightly larger than expected (105 kDa) from the amino acid sequence. The protein size was constant in the presence of tunicamycin, an inhibitor of N-glycosylation (Fig. 5B), indicating that NvHsTRPA does not contain N-glycans unlike the vertebrate TRPV1, 4, 5, and TRPC3 and 6 as reported [42]. The partial denature of NvHsTRPA (60°C for 5 min) may result in the aberrant migration through 6% SDS-PAGE gel which makes the correct estimation of protein size difficult. Nevertheless, the possibility that NvHsTRPA undergoes the other post-translational modifications can not be ruled out. Patch-clamp experiments with the cells expressing the tagged NvHsTRPA protein showed that it is activated by temperature increase, and quickly inactivated by constant heat application (Fig. 5C, left panel). Desensitization of the channel by repeated heat applications was also observed (Fig. 5C, left panel). Similar rapid inactivation and desensitization was also observed with DmPain [35]. The current activation was not observed with the mock-transfected HEK293 cells (Fig. 5C, right panel), suggesting that it is specifically mediated by NvHsTRPA. The rapid inactivation as well as desensitization properties of NvHsTRPA suggest that it is only once activated by rapid temperature increase during a short period (<60 sec). Thus, NvHsTRPA may allow *Nasonia *to detect the rapid increase of environmental temperature. To determine the temperature threshold for NvHsTRPA channel activation, we performed several experiments. As shown in an Additional file 1, NvHsTRPA appears to be activated as soon as temperature rises regardless of initial temperatures. The channel was activated even by small temperature increase (Additional file 1 right panel, RT to heat, a red dotted line), suggesting that it is very sensitive to temperature changes. We therefore lowered the temperature to ~7°C, and then applied heat stimulation very slowly (~0.25°C/sec) to activate the channel. However, the current developed gradually, and was small (< 100 pA, data not shown), demonstrating that NvHsTRPA activation is highly dependent on the heating rate. Thus, it is difficult to define the temperature threshold for this channel activation in HEK293 cells. NvHsTRPA may sense temperature increase without a specific temperature threshold, which requires further analyses. The current-voltage *(I-V) *relationship of NvHsTRPA showed dual rectification with a slight positive reversal potential (Fig. 5D), suggesting that it had high permeability to one of the cations (most likely Ca^2+^) included in the bath solution. It will be very interesting to compare these channel properties with those of TRPA1, which has not been fully characterized in any insect. We would like to propose that HsTRPA, originally created by the duplication of Wtrw, has gained the thermoresponsive property, and this has resulted in the loss of TRPA1 in Hymenoptera. If NvHsTRPA complements the functions of TRPA1, it could have roles in thermotactic behavior of *Nasonia *similar to DmTRPA1 in *Drosophila*. Knock-down of *NvHsTRPA *by RNAi followed by the analysis of thermotactic behavior will test this possibility.

DmTRPA1 was shown to be necessary for *Drosophila *larvae to discriminate between 18°C and slightly higher temperatures (19-24°C), and function downstream of a phospholipase C-dependent signaling pathway [40]. Under this behavioral paradigm, DmTRPA1 is not necessary to be heat-activated, and thus any TRP channel could substitute for the lack of TRPA1 in Hymenoptera. However, TRPA1 activation by temperature fluctuations appears to be established in both vertebrates and invertebrates. DmTRPA1 and the vertebrate TRPA1 are activated by temperature increase [39,41] and cooling to 10°C [43], respectively. Thus, the thermoresponsive property of NvHsTRPA demonstrates that it is a likely channel to complement the lack of TRPA1 in *Nasonia*.

## Conclusion

TRP channels respond to diverse stimuli, and thus function as the primary integrators of various kinds of sensory information. To understand the evolution of insect TRP channels, and thus the mechanisms of integrating sensory inputs in insects, we have identified and compared TRP channel genes in 6 genome-sequenced insect species. All the insects examined have 2 TRPV, 1 TRPN, 1 TRPM, 3 TRPC, and 1 TRPML subfamily members, suggesting that the mechanisms for detecting mechanical and visual stimuli and maintaining lysosomal functions may be evolutionarily well conserved in insects. Although *P. humanus *and *D. melanogaster *contain 4 TRPA subfamily members, *B. mori*, *T. castaneum*, *A. mellifera*, and *N. vitripennis *have 5 TRPA subfamily members. *T. castaneum*, *A. mellifera*, and *N. vitripennis *contain TRPA5 channels that have been retained or gained in Coleoptera and Hymenoptera but not in other examined insect orders. Furthermore, TRPA1, which functions in thermotaxis in *Drosophila*, is missing in *A. mellifera *and *N. vitripennis*; however, they have other Hymenoptera-specific TRPA channels which function as novel thermal sensors. The total number of insect TRP family members is 13-14, approximately half that of mammalian TRP family members. As reported for mammalian TRP channels, this may suggest that some single TRP channels are responsible for integrating multiple sensory inputs to keep the breadth of insect sensory systems comparable to that of mammals. These results demonstrate that there have been both evolutionary conservation and changes in insect TRP channels. In particular, the evolutionary processes have been accelerated in the TRPA subfamily, thus indicating the diversity of mechanisms for detecting environmental temperatures used by various insect species

## Methods

### Bioinformatics

The amino acid sequences of 13 *Drosophila *TRP channels were retrieved from FlyBase , and were used as queries to identify TRP channel genes of *Pediculus, Bombyx, Tribolium, Apis*, and *Nasonia *by TBLASTN search using the E value 1E-10 against the genomes and gene model DNA sequences. The genome databases used were VectorBase , Silkworm Genome Research Program , BeeBase , and Baylor ( and ). The list of amino acid sequences of insect TRP channels analyzed in this study is shown in an Additional file 2. The amino acid sequences of *C. elegans *and mouse TRPV and TRPM channels were retrieved from the NCBI protein database . The six transmembrane ion transport domains of each TRP channel were identified by InterProScan  search. The amino acid sequences of ion transport domains were aligned by the Muscle program [44], and then PhyML3.0 algorithm [45] was applied for the maximum likelihood analyses, under the WAG amino acid substitution model and with 100 bootstrapped data sets using the PhyML Online server. A phylogeny created with Neighbor Joining using the identity matrix and correcting for multiple replacements had an identical topology. The amino acid sequences of TcTRPA5 and AmTRPA5 were aligned with the CLUSTALW program [46], and the intron positions and phases were identified by searching the above databases.

### Characterization of NvHsTRPA channel protein

The full length *NvHsTRPA *cDNA was isolated by RT-PCR with adult *N. vitripennis *head RT and two primers, 5' TTTTTGCGGCCGCACCATGTCGCGCTCGTGGAAAC TGGACGAGGTC 3' and 5' TTTCTAGACTCCTCGGTTGCACTCTTCGAGCCACG AGATT 3'. The PCR product was digested with *Not*I and *Xba*I, and then cloned in pAc5.1/V5-His B vector (Invitrogen) in which the *Drosophila actin 5C *promoter was replaced with CMV promoter. The cloned cDNA was sequenced; it was identical to the one deposited in a database. HEK293 cells grown on cover slips were transfected with 1.2 μg *NvHsTRPA *expression vector using Attractene transfection reagent (Qiagen) for 2 days at 37°C. The cells were fixed with 4% paraformaldehyde/PBS for 15 min at room temperature (RT), and then permeabilized with 0.5% TX-100/PBS for 5 min. After washing the cells with PBS containing 0.1% TX-100 (PT), they were blocked with PT containing 5% normal donkey serum for 30 min followed by incubation with rabbit anti-V5 antibody (Sigma, 1000 fold dilution) for 2 hr at RT. The cells were washed, and then incubated with FITC-conjugated anti-rabbit IgG antibody (Chemicon, 300 fold dilution) for 2 hr at RT. The cells were observed with a confocal laser microscope. The cell lysates prepared from the cells transfected with empty vector (mock) and *NvHsTRPA *expression vector were analyzed by Western blot using rabbit anti-V5 antibody and HRP-conjugated anti-rabbit IgG antibody (GE healthcare, 3000 fold dilution). Tunicamycin was added to the cells at the concentration of 10 μg/ml for 15 hr where indicated. The signal was detected by an ECL detection system (GE healthcare).

### Electrophysiology

HEK293 cells in a 35-mm dish were transfected with 1 μg of *NvHsTRPA *expression vector and 0.1 μg of pGREEN LANTAN 1 using Lipofectamine Plus reagents (Invitrogen). After incubating for 3-4 hours, cells were reseeded on cover glasses and further incubated at 33°C and 5% CO_2_. Cells were used for the experiments 20-40 hours after transfection. The standard bath solution contained (in mM) 140 NaCl, 5 KCl, 2 MgCl_2_, 2 CaCl_2_, 10 HEPES, and 10 glucose, pH 7.4, adjusted with NaOH. The CsCl pipette solution contained (in mM) 140 CsCl, 5 EGTA and 10 HEPES, pH 7.4, adjusted with CsOH. Whole-cell recording data were sampled at 10 kHz and filtered at 5 kHz for analysis (AxoPATCH 200B amplifier with pCLAMP software, Molecular Devices). Membrane potential was clamped at -60 mV. The current-voltage (I-V) relationship upon heating was obtained by using voltage ramps (-100 to +100 mV in 100 ms). Cells were stimulated with temperature changes by decreasing or increasing the bath temperatures with an iced or preheated perfusate through the lines, respectively (with a minimum 8°C for cooling, and a maximum 44°C for heating). After whole-cell configuration was achieved, the cell was raised 50 μm and placed in the center of the chamber. Temperature was monitored with a thermocouple (TA-30, Warner Instruments) placed within 100 μm of the patch-clamped cell.

## Authors' contributions

HM maintained *Nasonia *and performed bioinformatics analysis of insect TRP channels. KK characterized NvHsTRPA channel protein. TS and MT carried out the electrophysiological experiments. TK designed the experiments and wrote the manuscript. All authors read and approved the final manuscript.

## Supplementary Material

Additional file 1 **Temperature thresholds for NvHsTRPA channel activation**. The representative traces show the activation currents of NvHsTRPA elicited by two different heat applications, 8-44°C (Cold to heat, left panel) and 25-44°C (RT to heat, right panel). The red dotted lines indicate the apparent initiation points of the currents. Click here for file

Additional file 2 **List of amino acid sequences of insect TRP channels analyzed in this study**. It describes the amino acid sequences of all insect TRP channels analyzed in this study. Click here for file
