# Supplementary figures and images for: Evolutionary conservation and changes in insect TRP channels
Source: BMC Evol Biol. 2009 Sep 10;9:228. doi: 10.1186/1471-2148-9-228 (PMC2753570; doi:10.1186/1471-2148-9-228)

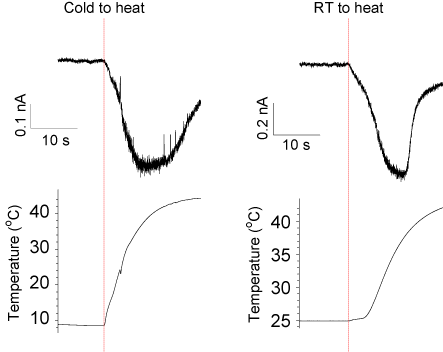

Supplement: Additional file 1 — Temperature thresholds for NvHsTRPA channel activation. The representative traces show the activation currents of NvHsTRPA elicited by two different heat applications, 8-44°C (Cold to heat, left panel) and 25-44°C (RT to heat, right panel). The red dotted lines indicate the apparent initiation points of the currents. [file 1471-2148-9-228-S1.tiff]
