# Supplementary material for: Evolutionary conservation and changes in insect TRP channels
Source: BMC Evol Biol. 2009 Sep 10;9:228. doi: 10.1186/1471-2148-9-228 (PMC2753570; doi:10.1186/1471-2148-9-228)
Supplement: Additional file 2 — List of amino acid sequences of insect TRP channels analyzed in this study. It describes the amino acid sequences of all insect TRP channels analyzed in this study. [file 1471-2148-9-228-S2.doc]

>PhNan

MGNTESNVTSGVKKQAGTSEQPLYKLVGLKGGGLLVDMMKRATQTKQYAELDHAIKTKIE

PFLYNKGKGRLIPISQLVLLRNCERSRTKLLPLLKNMEDPESEFDMDRDGNPGDFQPAPD

GVQEDFHDYNKKYRDVCWDLKKRGAVGETCLHLCMLNATSIHADLAKRLLKFYPKLINDI

YMSDEYYGESVLHVAIVNEDPAMVKYLLDSGSNFHERCFGNFMCPEDQKATRTDSLDHEY

VNLSTETNYEGYVYWGEYPLSFAACLGQEESYRLMLAKGANPDNQDTNGNTVLHMLVIHN

KLPMFDVAYEVGANLSLKNAKCLTPLTLAAKLARVELFFHILNIEREIYWQIGSITCAAY

PLSQIDTIDIETGNISKNSALNLVVFGEKDEHLELMDGVLVDLLVAKWNTYVKFRFYRQF

ISFFFYFLISVICFTLRPGPPTGTTTTTNGIVVLNNNNNNITSVVDNSNNVTTTTATTTA

TSYSFPGHVIVTLLSYDKYEDKIRIISELAMEIGSVLYLLGALREAKFLGIHMFIENLMT

APSRVLFLGSCCIMQFVPWLRITCKEETEDIVAVIIMLTTAPYFLFFCRKMNDPVFLFLF

FLIKKIFFLIFFFFFFTKPTYYIIFLSFDNPKTPDGVDDSGTNPMQSPVESIMAMFLMSL

TNFGEYYGAFEKTEHEFVAKLMFVVYMAIVAILLVNMLIAMMGNTYQKIAETRNEWQRQW

ARIVLVVERGVSPQERLKKLMLYSQPMSDGRRALVLRLHQTPEDKEEMKEILDMKRIHNR

TVQRRKAKENKNVTFAGTPKNGIKNRLLGPVPPLKSIPSKPKI

>PhIav

MGAGLCGTSQDPQNQGSVLDRVISQASNKDDCLLYKLANYKNSGELIEAYNIGGQAEVEK

LIKEQFGVLMYADGKGEVIKRAEYLRWKFRDQAQVVLPIEASLSIYDPLAKWEDHEACWQ

MQYRGSLGETLLHVLIICDSKIHTKLARTLLKCFPKLALDIVEGEEYLGASALHLAIAYN

NNELVEDLVDAGANINQRAVGSFFLPKDQQRAKPLKTTDYEGLAYLGEYPLSWAACCSNE

SVYNLLLDVGADPDSQDSFGNMILHMVVVCDKLDMFGYALRHPKVPASNGIINNEGLTPL

TLACKLGRADVFKEMLELSAKEFWRYSNITCSAYPLNALDTLLPDGRTNWNSAIFIILNG

TKEEHLDMLDGGIIQRLLEEKWKTFARNQFLKRLVIFFLHIFCLSGSVYLRPDDRNKPLL

GGTSVQDVVRYCFEIGTILGVLCYLCFQQGDEIRNQGLISFLKQLPHDPAKFIFLISNLL

ILACIPYRVAGDTDTEEAILVFAVPSSWFLLMFFAGAIRLTGPFVTMIYSMITGDMLTFG

IIYSVFLFGFSQSFFFLYKGSKNVSSSLFTSYPSTWMALFQVTMGDYNYNDLSLTAYPAI

SKMVFTIFMVLVPILLLNMLIAMMGNTYAHVIEQSEKEWMKQWAKIVVALERAVNQEDCH

RYLQEYSIKLGPGDDPSTEQRGVLVIKSKSKTRAKQRKGALCNWKRVGKVTIRELHKRGM

TGEQLRRLMWGRSSISTPVKPAPIKLGHVTSISGVADITVPETTGNTVGTGGGGLLAALD

VMAFTNDLEFSSENQTSTSFKTQPQPSEVSCVESDSNSDPLYNDPLRQLNEELNKTREEI

LTLARVAANNDGIEEIPPQICQYGWINNYNFFGNTFAQNQKVEDPVQQPTFQSTPNHSDS

DGLGDGLLLGSNRRLKRTRSANIKRKFSGSSTNEKNLLEPEDSSSTNSEDFSCLTENYIN

KNIKVNANFSENSLRSNRSSANLVVKKFGRRSGLKSSTNRIAPADLSPVGNVSKYSETLC

NSYCITSSSDVLYQWSIKGITNMNTLLGLENEDSM

>PhPkd2

MESGKNYQIVPYGHNLKLENETIVRSVFTKGKSKKNLFDPKKSIEKNIDENRSIDLSNSE

TVEKPANKDVIVIKSPKPRKTKKNKNSPNQKINKNKDKNKNDNDVDSDDEEDDEKLKPKN

ESGAIFFYNKFKKATKRRLTKTRLKLKSKLHPEQDFKGPYIYDFEVAVHSQTANMFYYKD

ITVQLFFNTPYLPDFINMSVQIDAENITTGKTLSESHTLSDFWSVKTTMINLFYESFFFP

PIDYTQYMDDDREMFYENEILGVPRMRQVRVEDNSCLIHPSMQRFFRHCFSYYSIYTEET

EPIKTGMEGTISETAWVYTEAEELNTSSYWGQISIYGGGGYYVDLGRTKESAIRIINELR

DNEWISRATRAVFIDFNIYNANVNYFCICKIVIEFPPMGGMIPNADFHPLKLIKYLWFWD

FVVLGLEFVRFVFLIYFIYILVNRILTQKLQFFFSFWNIFEFFLIALSIIHYILALYLYV

LVSKTIKSLAERFTLYTEFDYIKQLHLLYGTNFAFIVLFSWLKLIKYLNYNTIMVEFQDT

IARSVGSILAFFPMFLSMFTGFSLLGFLLFGSQIRDYRTWWSSSLSLLQTIIGEFEYQEI

QNAEPVFGPIFYIVYIFFMVFLALNVFIAIITDAYTHVKTKVTTRVPKHGHYLKRGFMNV

LTPCGWDKKFAKFNVKEKAGFTDSEIKIFFSKYDINMNNILSEGIKDKIINDLEFKIADY

EESAEEFEIKLLKRRMNEVYDKIEDIEANLQPILDKVDALLHKY

>PhNompC

MSGTGGRKGGGGGGGGKKKEDGNNESKEDDTGSGNGDKKDSNVKNDKTSNNGDVKPGSAG

VSIREGSHRLLALAMRGEWPPVDQVLKAIEKAVAAGGDDVNSTPLSGVVDPITGMTPLMY

AVKDNRTTFIDRMIELGVDVAARNFDNYNALHIASMHSREDVIKLLLSKKGVDVYVTGGM

KEQTAVHMVATRQTGTATSILRILLNAAGKEIRLRTDGKGKIPLLLAVESGNQSMCRELL

GSQAADQLRATTPDGDTALHLATRRRDIDMVRILVDYGAAIDLQNGDGQTALHIAAAEGD

EVLVKYFYGVRASASIVDNQDRTPMHLAAEYGHANIIELLADKFKASIFERTKDGSTLMH

IASLNGHSECAQMLFKKGVYLHMPNKDGARSIHTAARYGHVGIINTLLQKGEKVDVTTNE

NYTALHIAVESCKPLVVETLLGYGADVHITGGSHKETPLHIASRVKDGDRCALMLLKSGA

GPNITTEDGETSVHVAAKYGNATTLSLLLEDNGDPLFRNKLGETPLHLACRGCKSDVVKL

LIDFVREKKGPEVATSYINAVNDNGASALHYAAKVSKTEVERPLEDQEVVRLLLESGANS

SLMTKHALETAFHYCAEAGNNDVMTEMISQMNATDVQKALNKQSAVGWTPLLIACHKGHM

ELVNNLLANHARVDVFDLEGRSALHLAAEKGYIEVCDALLTNKAFINSKSRVGRTALHLA

AMNGYADLVSFLIKEHNAMIDVLTLRKQTPLHLAAAAGQIEVCKLLLELGASIDATDDLG

QKPIHVAALNNYSDVVQLFLQHYPSVVTASTKDGNTCAHIAAIQGSVAVLEELMKFDRQG

VIAARNKITEATPLQLAAEGGHADVVKMLVRAGASCTDENKAGFTAVHLAAQNGHGQVLE

VMRSSQSLRVSSKKLGVTALHVAAYFGQADTVRELLTYVPATVKSDPPSGVGLVEELGAE

SGMTPLHLASYSGNENVVRLLLNSAGVQVDAATTENGYNSLHLACFGGHITVVGLLLSRS

ADMLQSADHHGKTGLHIAATHGHYQMVEVLLGQGAEINATDKNGWTPLHCAARAGYLSVV

RLLVESGASPKSETNYGSPAIWFAASEGHNDVLEYLMTKEHDTYSLMEDKRFVYNLMICG

KNNKNRPIEEFILVSPAPVDTAAKLSAILVNLATKEKERSKDLVEAGKQCEAMATELLAL

AAGADSAGRILTALDRRNIEFLDVLIENEQKEVIAHTVVQRYLQELWKGNLMWSAWRLMF

LFLLFIICPPIWVFFTLPLGHSFYKVPIIKFMSYLTSHIYLMLFLMLVGITPIYPVVRNT

LLPYWYEWTLLVWLSGLLLNELTNPSDKSGLGWIKLAVLLFGIFGVALHLLGLFIDRPYW

PTLMYLRNQLFALSFLLACVQILDFLSFHYLFGPWAIIIGNLMKDLARFLAVLAIFVFGF

SMHIVALNQPFRNLTPQQVQANVKPHPYQGLFGEVRMNPLMAFELLFFAVFGQTTPQNLQ

GTTIQPWWTLILFKITGGIYMLVSVVVLINLLIAMMSDTYQRIQAQSDIEWKYGLAKLIR

NMHRTTTTPSPINLMTTWLIILLRFCKQKVIQRKRPSLVQMMGLQSRSRVSPRTKVATKW

LAKVKKGQVAPKDPVALSVVHLSPLGSQMSFGNATRIENVADWEAIAKKYRALMGESSED

RMGKDTNQQDVTSSTITLLENNNNNNNNNSNNNNNNATS

>PhTRPM

MNFEKKCCCGLSLTHHRGTGAKIQPPYESQWISSRHTISVPTDAYGTLEFQGGPHPSKAQ

YVRLSYDTRPELILQLFTREWSLETPKLLITVQGGKANFDLQPKLKKILRKGLLKAAKTT

GAWIFTGGTNTVTRQVGDALLLERSQRSSRIVTIGIAPWGIVENNYELIGRNRDVPYHSI

SSPRYGAEIILRRKLEKYISIQKLHPCTHCSTPVVCLVIEGGTNTIRAVLEYVTDDPPVP

VVVCDGSGRAADLLAFVHKYASESGEQTVLENMKDYLIGTIQRTFEVGSEQSECLYSELL

QCTQCKTQELDQTILTALFKSQHLSPSEQLSLALTWNRVDIARSEIFVYGQEWPPGALDE

AMMQALEHDRIDFVKLLLENGVSMKKFLTIPRLEELYNTKQGPTNTLGYILRDVRPHIPR

GYVYTLHDIGLVINKLMGGAYRSYYTRRKFRLIYAKVMKKSPHVHRNSASFIRYYGNQNL

TLSLLAEALPATSDTPLFDYPFNELMIWAVLTKRQQMALLMWQHGEEALAKSLISCKLYK

AMAHEAAEDDLETEVYEELRNYGKEFENIALELLDFCYRQDDDQAQQLLTCELQNWSGQT

CLSLAVAANHRALLAHPCSQIILADLWMGGLRTRKNTNLKVILGLLCPPYICQLEFKSKE

ELQSMPQTEEEHLTGLEEENESVDGEMKFNQTHRNTDPETEALFAQDFGVKDTIIQENGK

VITEDDTEIGNSFHHIPLDFHDVKAARPLRLKKKFYEFYTAPITKFWAHSLAYMVFLMFF

SYVVLVKMENMPSWQEYYIITYICTLGFEKIREIVSSEPVAISHKFAVWTWNMWNPCDSA

AIIFFLIGLALRLKPSSMAIGRVIFCVNIVYWYLRILNILGVNKYLGPLVTMMGKMVKNM

IYFVVLLLVVLMSFGVCRQSILNPNREASWTSVKEVFYQPYFMLYGEVFAGDIDPPCGEH

PTDSPCPTGRWVTPVVMSIYLLVANILLINLLIAVFNNIFIEVNAVSHQVWMFQRFTVVM

EYEQKPGIPPPLIIISHLFLLFKFVRRKLKGIEETYDNALKLFLDNDDLERLYDFEEECV

EGYFREKDLKLLQSSDERIKTTTEKVENMYQKVEDINQKENAQTTSIQSVEFRLRKVEDV

AEQILNHLDVVHRFMATHTRDTAFPDFPEVNIVADRTRTASERSEDVKVMAAPDSEFAER

LLSLTEVRPTDRLCLSAERIYVDPKRHEIPLENSNNSNQKGEDEVEYPGDEKSFIDRRLS

ESSAAEKHEDVSVCRESTKGSHVEVNRPQETQEIERPLSSADGSVGLRKRRGSSTEEKSD

RGSVLPLCHLSTRRQLSQTHSEPDVDFLGLGSSVPRAVTVERSVTWAEPRITVIPPTAST

ARSMLLAMHSEYTSITDELETVCGLLSPPRTPSLLTPTTKKDTVHPKRTRHTSEMSNPEM

AIYIEKEHLRDAEESDYQLMQDLIDKRFYIGENSEIDERFDNNAFFLTVTDEVNDYRSPF

QSRYLRRSSAIEIKDQPQSNDGSQSNSQLQINNSVSIDSSGPCQVEVCLSSRGNSVEEPQ

VIVKEHPASDETNANEVFESDDVTRAKYLNDKCDNKGSVETSDETKQSSHFQSGETDSKN

GGRQQQQQQQQQQQQQQLVQEPQEYQIKKIHLHRNNSETEGSTSTKGTTQQSSEISNETH

FLYREHIKPTTGRKLSRQESQGKVGRTNTLAMDFEMGHNSKKISSQPSLPPQKEEESSSP

PENEERGSRDSLMMPSETMC

>PhTRPML

MTTETNNELDDDRILEESLRENAFSGFDERLIEEESLKRKLKFFYMNPIEKWNNFMFSNK

ISFCHLFLSGWDATREVIAYPPQSGDLAIYKISDFYTVIDYAVINYANLDDAIGSYKYPN

NSSMGHIQMCLKLKNGLNESDNCKHYNCYNLNDFQIRVECLTIIVTKENSKNFSIKNYFD

ENKIKLEFSSLIDATLSFEIKTVHSNLISLNCFSFNISIKFDNKHNSGQMKYDLTLNIKG

LNCDKKYFNPPYSIIISFLNFSVIIICCLSIILCVRSLLRTQYLKKKTIYHFKKYYSRNV

SWEGRMEFFNPWYVIMIFGDVLILISLVFRYKIEEDEFSGDELNMCSLILGCGNFLVWFS

LLRYLCFFKTYNAIILTLKMAAPNIFRFMVCVLLIYSSFTFSGWLILGPYHMKFRTLSGT

SECLFALINGDDVFATFNTLQPTDSKTIWYFCRIYLYVFISLFIYVVLSLFLSIVEGAYE

IIKQSGKDGFPKSDIKTFIETSSNNSLQSLASSSYPDCTLICNADNLKLKNITAKICCCQ

SLLKNPRT

>PhPHUM001594-PA

MKVTESREELFKKSIEDLEDSCDYNLSALAETFLTPTEKKFLLCAEKGDIPTLRSLLIQF

EDRPDELNINCTDSLRRSALVTAIENENMDLIKFLLKSNIKVKDALLHAIKEEFVEAVEV

LLEWEEKIHVDGEPYSWEAVDSASATFTPDITPLILAAHTNNYEILKILLDRGATLPRPH

NVRCGCDECLASSEADSLRHSQARINAYKALSSSSLISLSSRDPLLTAFELSWELKNLSY

CEAEYRSEYNEMRQATQDFATALLDHARTSGELEVMLNYNPNGDIWEPGERHTLERLKLA

IKYKQKKFVAHPNVQQLLASIWYDGLPGFRRKSTVRQITDVLKLACLFPFYSCIYMIAPN

SKMGLFVKKPFVKFIFHSSSYAFFLLLLGLASQRAENTFLELLAMEIEWLHDVLAEWKRK

ERGSWPGVVESALYLYIISLIYSEVRALWTNGLKEYMADLWNIVDFITNCFYLVWIGLRF

TAIYIVMRESNLGLDPYIPREQWHPFDPMLLSEGAFAGGMILSFLKLVHVFSVNPHLGPL

QISLGRMIIDILKFFFIYMLVLVAFGCGINQLLWYYSNLEKVKCFGEDGTLEPDFDNNEK

ACTVWRRFANLFETSQSLFWASFGLVDLTSFDLVGINSFTRFWSLLLFGCYSVINIIVLL

NMLIAMMSNSYQIISERSDIEWKFARSHLWMSYFDDGDTLPPPFNLLPTMKTLLRIFKTE

KNLTADSFKVKSMDKARKRHELVMNNLIARYVTAEQRKRCDYGITEDDVIEIRQDISSLR

YELVHILRANGMKTPEMNPDQNLVTKKGKIMERRLEKDFRIGIVEGIIHEFLSSDKDSLD

IFGKIAKVFNRKRNKSKKQDWNTIVRKSSYQKNLIGSSEERRERLAKQSVRRYIIEKLSS

SEALDPEKLVQYNPQLLQMAPAARIAYTKFSRIKITREFAEQEKIINEEIEEEKHAEELV

KLTHSSTERKKDEEQPPNDTTDFSIVRPRPQSRSNSKLMKLISIPSKEGTETKDCIIKMS

EEDENETIVTVENEIKQEKKTSSISIQSPPSLNDVIPTVVSIESPLKDVKKFEPVAAIIR

QMSIKSITTSPVQKSPPTDEMTPPTPPIPIIKKPEMKTDENEENNKFTSLDIKGSRSPLA

DSSRGRSKVTGEIISGWL

>PhPHUM003044-PA

MSVNYLDMEEGRLPQAPLSYIPKDPDKSNYKNKVKRHSIHGMMEEENVVRPHQEMAQLSL

DEKKFLLAVERGDVASTRRTLQKAQETDFININCVDPLGRSALLMAIDNENLEMVELLIE

HKVETKDALLHAISEEFVEAVEVLLDHEKTIHKPGEPHSWEALPPDTATFTPDITPLILA

AHRDNYEIIKILLDRGATLPDPHHVRCGCKECVTSRQEDSLRHSRSRINAYRALASPSLI

ALSSKDPILTAFELSWELRRLSFLEHEFKIQYQIKTRSKQCQDFATALLDHTRSSYELEV

LLNHDPTGPAFEHGERMHLNFVAHPNVQQLLASIWYEGLPGFRRKNMILQAMEIVRIGIL

FPFFSLMYILAPHSKIGQTMRKPFIKFICNSASYLTFLFLLILASQRIETVVGDLFGLTN

DGMKAPDITTKRGALPSIIEWLILFWVAGLIWSEIKQLWDVGLEEYVHDMWNVIDFVTMS

LYVATVALRIVAYYRVQKEISEGTGSNRLYIELQREEWDTWDPMLIAEGLFSAANIFSSL

KLVYIFSVNPYLGPLQVSLSRMVLDIMKFFFLYVLVLFAFSCGLNQLLWYYADMEKKKCV

PEYDLGKNMTKNLSSDRTACLVWRRFSNLFETTQTLFWAVFGLIDLDSFELEGIKTFTRF

WGMLMFGTYSVINIVVLLNLLIAMMNHSYQLISERADVEWKFARSKLWISYFEEGGTVPP

PFNIIPTFKSMWYLSQWLYRKLCGHSRAAKKEHMRTIRSIMRNLVRRYVTVEQRKAESQG

VTEDDVNEIKQDISAFRCELIEILKNSGMNTSTASGMGAGSGGKKNRQKERRLMKGFNIA

PTPGPGVPLAPVAEFIANLQAEPEILQHQDLFGSTISGIIGTPNTVPKKSNQLRESMHDA

PSSAPPYVQTGSLSTSLPSQKSLSKLTPKFNISKRSSSRKKRWGTLMEAAKSGKVSKLIN

RSRSEDSVCNS

>PhPHUM009153-PA

MATNPHRDRKGSRSHEHRDRDPREITDRVYEEDNAEERERELFWNNNTPNAQQNPKNTFA

PDDRGSRFLIREREYYTVHDDPETQRRRQQEEPGEVIHNVDNNRRRASRTEPENVEPHFN

PNLRQDSSKNFNKNRREFTTDERNSKGEVEHHYSHYQNRSREGPLNRREPHHEQEIPPDF

NEKGYFDPFKENHKRQGSLHGRSPMATTPLPDINHDAPLPPIPKYSRKYGSPERIPEKTY

SKNDLHEIQPKENSRMIKEEHNMADNNHENKQDIYKEGELMRGDKKPESHLNLVSAETFR

HLSKQEENAGGGGPAEFSRHKTTGEHTGSKKAFNRISHVGSKSRVYEREKDETLRRRSIN

AGTGGGTGMGTGSKMNKGPRTYYILKEEDDDIERKASVLLPVLEDIEKVFHDLVASGDVA

ATKNFLNNHQDFNINCVNYQKISALHVAVKQQNEEMVEFLLEQKGIDISDCALYAIKEGS

IKIVELIFEKIREISPGLEFAGTTHSVDFSDDLTPIILAAQLGNYEMIDFLIKRGHGIPP

VHPPFCKCDACKADLKKYDRLKYSTMKLNLYKAICNPMYIFHTRDDPFQRAFELTKELQD

AATVFPQFKTSFEELWCSLRTFTVELIGCCRNAQEVEIILKKSPKSVAEHVKFPRLQDAI

DLKQKEFVSHPYTQAVLSDAWTGELFEWKIRSPLMQSLAVFPRIPMLPFMTILCMFLPNH

SLVRKWKTPVNKMISNVASYLIFLIILFLESNLDKTKQKRGPPNSGLEIVIIIYVIGYIW

LNIRTCFKQGLKRFFSTMWHWYDTIMLLLFALTIIFWIASLVDITANNDVDLPRKYWNSL

DPELVAEGLFAAAVIMAFFRLNLFLSLNYHLGPLQVSIGKMTLDIFRFIIIFFIIILAWC

AGLCRFYQPYDGMVQKDAVSGSTTTQVSSFVSFLMTLKTFFWAIFGMSPIESADVIIENL

PGDTPDTTIINKHQFTEAVGYIAFALFQFLSVVVVLNMLVACMANTFTKVIDNVTVEWVF

GRSQIYLIFMAYSVLPPPFNLLPTASGIKDMCKWFRLLARPTEGQRAKCNLAHCCYIESE

TEAYVNNEFPSLISELAQRYIRLKVQEGEEQQTNQEIEELRNELHEIKEIIKKLYPIKRS

NIY

>PhPain

MSGKLPVECFEMTSKDFCSTNPAHVQNDLLNAWRKKDITNFLSVLSSNECDPNFLYENDN

YMRLLDSAVENGNVEFAEAIIKAGADVNLINPVRSKAPVHFAAETGQLELLKLLVSRGAD

INIRDSSGSTILHYVVKQKWPPGEQKFKKCLAYLLELDSLKVNMTNRKGLTAIHLAAMQG

SGEAVKMLLEKGSHLDIDSIKVGSARRTARQEIMHKFPHLEKDLPSPRDIYDIKMDGDKL

FSLLHDNHEDRFCETFNEEMKDKAQKNLLKYTDGKQTFLQYCCEKKFSKAVKTLLEYDVD

VNYAPNNSTRPVLIACRKGDSETLRLLLSAKPDLSCNEDGENPLHIVVKYTDNSPDHLDC

LQILLRRLNKSNLDINAQDLKGNSPLHYAVKYGGPACVEELLKNGAYIGTLNKFKKPPLA

DMSSSTLGNYLDSCITATDVLDRGNNYVIKFNYNFLVPKNCLVHSELEDIKIPLTDDNET

VNLLQESIPESQPLLYMSKDSHLRKLLVHPVLTSFILLKWQRVRIFYYVNLIFYVLFCFL

LTVYILFGCVEHTSENVLILTTVAINPEDFLGGGGNGSNEGKDIFNESPGVDFLRVCLII

FLIALTLRELFQIVIHPKKYVLDPENWLEILLLIVLAFVLVKDCSREFPKSTRLGLWCPQ

FSALAILLSWFELVLLAGKHPLQSLHIEMFKTVSINFMKFLAWYVILIVAFALTFYTLFR

DCGADCEENNPFVNPGLSIFKSIVMLTGEFDASDIPFVTFIGTSHLIFIAFIFLIAIVLL

NLLNGLAVTDTQTIRNDAYLCRCIALVKLISYIENMLLVESDPIPCLNLFKCCSKKKKNN

NFNLNCFDYLAKRIVFFPKLLKDGIIYVLPNQRYKIYFEDPKSIKEEDTICYNRLTNYYL

DPSTAKTAERILSERVSSKGNPVAEMDTLRNEINNCTERIFEMEKKMESLEQSTKQNEKL

LIQILAILSLNNANNDDK

>PhTRPA1

MVPLRDNIRNFLPNFICRRDTVTPKVTPPTIVVRNWKKVLLGFPFSQKLSPDELIEAAES

GNLEDFGRLFMADPNRLEIRDSKGRAAAHQAAARNKVNILQFIHSHGGDLNIRDYAGNTP

LHVAILNDSYNVMDFLLQCGVNTSILNEKNQAPIHLITELNKVKALEVLSKHRSKIDIQQ

GGEHGRTALHLTAIYDYEECARILITEFGACPRKPCNNGYYPIHEAAKNASSKTMEVFLQ

WGESRGCSREEMMSFYDSEGNVPLHSAVHGGDIRAVELCLKSGAKISTQQHDLSTPVHLA

CAQGAIEIVRLMFKMQPHEKEICLTSCDVQKMTPLHCAAMFDHPEIVEYLISEGAEINPL

DKENRSPLLLAASRAGWRTVLTLIRLKANILLKDSSYRNILSLVVMNGGRLEDFAQEVLK

VQSKKDLLLLLNEKDISGCSPLHYASREGHIKSLESLIKLGACINLKNNNNESPLHFAAR

YGRYNTVKQLLDSEKGTFIINECDGEGLTPLHIASKNGHSRVVQLLLNRGALLHRDHYGR

NPLHLAAMNGYTQTMELLHSVHSHLLDQVDKDGNTTLHLASMENKPNAISLLLSLNCKLL

YNYLEMSAIDYAIHYKFQEAALAMVTHPTRSCEVMALKSDKHPCVTLALIASMPKVFEAV

QNGCITKANCKKDSKSFYVKYHFSCLQCPTIYAQVDEKTGETLTITNPIPLPALNAMVSH

GRVELLAHPLSQKYLQMKWNSYGKYFHLINLLFYTIFLTVVTTFTAHLMHSNITTTCEES

NQTVVLSEISLRLESSSNYTIIYTTAVAIAVYVSVQLFRETIQMYQQKWNYCMDPSNFIS

LGLYISSITMIVPIFMDKCTDLQFSSAAITVFLSWFNLLLYLQRFDQVGIYIVMFLEILQ

TLIKVLLVFSILIIAFGLAFYILLSGGSHLSFSTVPMSLMRTFAMMLGEIDFLGTYVQPF

FRKPEDSDDVSEDKIKTLPYPFPAFFILGIFMVLMPILLMNLLIGLAVGDIESVRRNAQL

KRLAMQVVLHTELERKLPKCLLDRVDKMELIEYPNEKKCKLGFLDTLLGKWFFNPFSDDV

GWEMVVDNTEDYLTSEMAKQKKKLKEISSCLETQQLFLRLIIQKMEIKTEADDVDEGISP

NALKNLLNRSSSSYTSPYARKKLRSSLSITKSSNY

>PhWtrw

MELNFTKDYDQKQPFDRSISFGRELSVIDTSQKCRTPSFLERLKSFGEDRASMLQRSKHI

DLEDSSSSALDDIIFLDSFEEIHQKGDANVLVSRDVVRQNILDMMKSGSGNVKLLTDIET

GNTSVNNIEEHFANSSQFEKNVGFLWSVFHRRRDLLDYFLQFNVDVNFSEPQLGFTPLHL

SSFSGCNNCTALLISKGADVNTLTNKYTPLHCAAFGNAASTAKLLLKAGAKIVSEKVCNE

TPIHSAVRSKAVDCLQLFITENMDLSNMGGCGYSPLHLAADLGHLQCLQILLNSNKCDIN

LQTLERKCSALHLAAENGYYECLKTLLEYGADSNAVNYRQQTPLHAAAKVCSESVELLLK

YGADPNSPDADNRTPMHAAVCKCESEHSLEILDYLTMWKGDVNRRDKYGYTPLHIAALNE

LTECVECLILSGSDVTARTRGGTSALSIISRKTPAALGAVNQKLDESITTNDPDASKEIV

LMFNFRYLLKNCKRGEVSFLQTLVDEGQKEFLSHPLCEAFLHLKWQKIRKYYITRLIFCL

LFVILLSLYVLTALAYQCYNDSVSDVFPDVEARILIVDPELCTKKSYFGELLRRNPIIME

MEWFFLVFITVLEIFRKIYGFRGYKSFRHYISQKGNIVEWFVVLSVFLISFVYTGRTYIW

QNHVGALAVLCGWANLMIMIGNLPIFGSYVAMYTKVQKEFTKLFLAYICLLIGFTVCFCV

IFPKAKQFSNPFVAVIKILVMMTGELEFDSLLENITQEESHSATLLEISALTTFVFFLLF

VTIILMNLLIGIAVHDIQGLQKTAVLSKLVGQTKLITHIESALFNGCLPNCFLKLLQITA

LISPSSYRVVVSVKPLNPRETRLPQDVMRSGLKVAKQKRAKGPTPSARSSMRYPTMKKRK

TMMMMNEEVIADMRNALSKLMDEVGTIKRLIMAGERNDILFPLLLTDMLGKVDIECTVEN

GGPSGQAGALRYAISKTLYTLVDENVRAKMMLAGLTQRDIRRRERKKPGRDGARRRYTWL

KR

>PhPyr

MNGKRDVEEQEVIDDDDDDNVSNETEEIGAAGNDTDQPQSLQIQEIWHKKEILESLNILP

GSQELQSLIAARDYKRVLSHPNSSLTGLLLASFSGNAELLQAILRRSMVVNVRDREGRTP

LHLACCAGSAECVKILLDHKAMPNVWNKEGVITPLHCAASVGNIQCLKLLLDAGAILDAG

LTTTGFGKTPLYYAVLSNSINCVEELLKRNASINTSQVYTETPLHVAAAMGFASCLKLLL

DHGADFRVKFGTAKSTPLHLAAEDGNAECAKLLIEAGADLMSRTNRIQTPLHLAALAQSV

QTLELLLMHRADPNAEDIDKRTPLHCAIVKSSRSCDSVQLLLQYGAKVNAPDVFGYTPVH

IAALNEFPNCLKLLLDHGGDVTKRTNGNVSALSFIARRTPEILKYLERKLDQGIRLHDHE

IGDVDCEIKLDFRVLVPSLSSGIFLDSCADFKKCTIPMSFRYVSYVLLVCNFSILGKELF

QIAHIRRGYIYQWENWLQWLIILAVFVTLVPPNWLSLKFVTWQHHIAVFGIFFNWIELMV

LIGRFPMFGLYVQMFTKGAVNFGKFLLAYFCLLAAFSFSFRMLFPKYPSFNSTINSIVKI

VAMMTGELEFEDIFFNSEDPLYYPGTSHIFFLFFTLIVTVVLTNLLVGLSVSDIQGLQTS

AGLDRLTRQVELVAYMESMLFSRLLNWIPKKILRVCHRSALLLSSPRQFTLIIKPNDPRE

KRIPKELVKEAYKCVSERRGIQSPVLKTEFITNYFERNRFPHSRHEIYVSKRKKRNK

>DmTRPA1

MTSGDKETPKREDFASALRFLMGGCAREPEMTAMAPLNLPKKWARILRMSSTPKIPIVDYLEAAESGNLDDFKRLFMADN

SRIALKDAKGRTAAHQAAARNRVNILRYIRDQNGDFNAKDNAGNTPLHIAVESDAYDALDYLLSIPVDTGVLNEKKQAPV

HLATELNKVKSLRVMGQYRNVIDIQQGGEHGRTALHLAAIYDHEECARILITEFDACPRKPCNNGYYPIHEAAKNASSKT

MEVFFQASYPFHFPIRTCPSCMLQLRWGEQRGCTREEMISFYDSEGNVPLHSAVHGGDIKAVELCLKSGAKISTQQHDLS

TPVHLACAQGAIDIVKLMFEMQPMEKRLCLSCTDVQKMTPLHCASMFDHPDIVSYLVAEGADINALDKEHRSPLLLAASR

SGWKTVHLLIRLGACISVKDAAARNVLHFVIMNGGRLTDFAEQVANCQTQAQLKLLLNEKDSMGCSPLHYASRDGHIRSL

ENLIRLGACINLKNNNNESPLHFAARYGRYNTVRQLLDSEKGSFIINESDGAGMTPLHISSQQGHTRVVQLLLNRGALLH

RDHTGRNPLQLAAMSGYTETIELLHSVHSHLLDQVDKDGNTALHLATMENKPHAISVLMSMGCKLVYNVLDMSAIDYAIY

YKYPEAALAMVTHEERANEVMALRSDKHPCVTLALIASMPKVFEAVQDKCITKANCKKDSKSFYIKYSFAFLQCPFMFAK

IDEKTGESITTASPIPLPALNIKYSFWPYQKTPEQIEAKRKEFNDPKWRPAPLAVVNTMVTHGRVELLAHPLSQKYLQMK

WNSYGKYFHLANLLIYSIFLVFVTIYSSLMMNNIELKAGDNKTMSQYCNMGWEQLTMNLSQNPSVASQIRLDSCEERINR

TTAILFCAVVIVVYILLNSMRELIQIYQQKLHYILETVNLISWVLYISALVMVTPAFQPDGGINTIHYSAASIAVFLSWF

RLLLFLQRFDQVGIYVVMFLEILQTLIKVLMVFSILIIAFGLAFYILLSKIIDPQPNHLSFSNIPMSLLRTFSMMLGELD

FVGTYVNTYYRDQLKVPMTSFLILSVFMILMPILLMNLLIGLAVGDIESVRRNAQLKRLAMQVVLHTELERKLPHVWLQR

VDKMELIEYPNETKCKLGFCDFILRKWFSNPFTEDCKLSGKRSGTRSIQYTFNFYAASMDVISFDNNDDYINAELERQRR

KLRDISRMLEQQHHLVRLIVQKMEIKTEADDVDEGISPNELRSVVGLRSAGGNRWNSPRVRNKLRAALSFNKSM

>DmPkd2

MAQQGQSGRSPSGPPGPPPPRPPKTPPGASPRGTPTADSTPGDVTPARGPPPPPATKRVSIGVGSTRSAPPPATDPTGPP

PTAGKPVRDTGPSTSPRPSLRDPGQSTSDKPSTSDKPSTSDKPSTSARTTVREPGPSSSPSRPVSTRESGRITFQEAAPS

TSAKVVKLAASEPRPAKPPEKEGFSLFKRKKKSVGSSTPPSPSRGHRAPSIIAASALQNRAKTNRILYTTDEEVREALVE

FSVFIIFLILTSLVVLSVRHTYMFYFNDTMKKLFTNREMVVAPSVTVGFEKLITVPDWWDYLKYNFLVTLHGDLTFMDDA

QANLTSMPPEGSEQGSEQGSEQGSEQSPEEVVGEAPEEAPEMDRLQEDFKYNGNPYIDLRGHRRAKRQSGVDGNSEDGSE

DVSGNYLKDDSDSIQANMSFHHLEGRVFLYENLLLGPPRLRQIRVRKESCYVNDAFIRYFNTCYAAYSSGAEDRKPMHKG

SPFRTMHDLDSTPIWTVLAFYRTGGYTVNLDYDKDRNVKIINDLKDIHWLDRGSRLCLVEFNLFNENTDIFQSIKLIAEI

PPTGGVIPQAHLQTVKMYSFFTDRSMLMTVIYIFWYIMVIYHTIYEITEIRKSGIKIYFCSMLNILDCAILLGCYLALVY

NIWHSFKVMSLTARAHSDVTYQSLDVLCFWNIIYVDMMAILAFLVWIKIFKFISFNKTLVQFTTTLKRCSKDLAGFSLMF

GIVFLAYAQLGLLLFGTKHPDFRNFITSILTMIRMILGDFQYNLIEQANRVLGPIYFLTYILLVFFILLNMFLAIIMETY

NTVKGEITQGRSHLGSYIYRKLSGMLYWITHCGRKRRHHPQASETEDKDAEHDVGAAHDETHEIRKNMTPAEQQYFKDIP

QGENQDMVRLNNRVGLLEEILEKLINNMDDILKRVEKDYHNKKK

>DmPyr

MENVRFSIIENDLKWNSESDPTADVDLLLDKRSISSEANSAGVFSDEAHE

IFARQQQNQILWDLDEVKETLTESPGGKHVVDMVQSGCFLELMTDSADCN

LALICCSVFGSVENTLFLLKHYNADPNVADSRGRTPLHFACCRANAPIAK

VLLDFGADPNRWDARKEVTSLHCAASSKSVECILLLLRRKASINIGIEKR

SALHYAIDVNAVDCVEILLKYGADPNTPQVYTETPLHTASAAGFAKCVQL

LLSHNADVRSQFGEGKVTALHLAAENDYVECARLLLEHRAEVDCRNASHQ

TPLHLACLSQSIGTVDLLISYGANVNAVYRDGRTALHAAIVKQSRSLDCC

NALLKAGADVNKADNYGYTPLHIAALNEFSSCVYTFIEHGADITARTDGR

VSALSFIVRRTPEIIPKLMQKLDSSIKANDQEIGDVDCQIKLDFRLLVPS

SSMDRGETELLLSLIEVGQKRILMHPLCETFLFLKWRRIRKFFLMSLAYH

TLFVILFTFYVIWVYVRCCKKEELCVAPGYVSTIGYLVIILNLILLGKEV

FQMAHGLRGYAKYWENWLQWTIGTGVLLCVTPETVRTDDLTAVPVWQHHV

AAIVILLVWLELMMLVGRFPIFGVYVQMFTKVAVNFAKFLLAYICLLVAF

GLSFAVLFNDYPAFENITWSFLKSITMMSGELEFEDIFYGDYAVKFPVTA

HIIFLSFVLLVTVILTNLMVGLAVSDIQGLQVSATLDRLVRQAELVSRLE

SLFFSRLLRSAPTNLIQLCKRSALLRTSRDKLQFTIRPNDPRDNQLPEDI

KLNVYKLVAERRDRNQSLRRRQFENNYNIFSRSLQRQQQPLHTDFLKPEP

ATGTTKKTPQNLFHMHELLRPRSATNVPQQFRQEAEGTVQMKNQANVLSA

VLAEVQAIKTQLVDLVAKFERFSENATRKLNYSTDELCRLRQQGQSVASS

HIRRHR

>DmWtrw

MENLGYKEGSTKPRRMTRSISVVTKTEVEQPLTENNANNRFKSIPPNLMVRWRNNTDAMIEAQYPTAGEFEYMECGPSPP

AESAPSMYDSFEEPTSELSVQICNDTLRISLIDQMKSAAGRVKLFEDIEQSNVVAEGIRTHFESASKLEKNLCYLWAAYL

KRWDLIESLLEAGADLHFCDQNGISALHLSAFSGCLATLGLLVAKGLNVNLQSKCYTPLHCAAFGNAAEAAKLLINNGAD

ISKDTSKPNCEESLLHCAVRSNALECLQIFIAEGADVNSLKPNGTNAIHLAADLGNIQCLEALLNAPNADANVRICIREK

ESTALHLAADEGNVECVDLLLAKGADAKLKNHRGFTPLHLAARTSSLDCVESLLRNGNADANAEDFDHRTPLHAAVGKSE

NAYDIMETLIQWGANVNHKDIYGFTALHLAALDGLVQCVEMLIFHGADVTTKSKKGTSALNVITRKTPASVAMIRQKLDA

AITLHHSQDPVNREVELELDFRQLLQHCHPREISYLNTFVDEGQKEILEHPLCSSFLYIKWGKIRKYYIGRLIFCFSFVL

FLTLYVLTALAHNCYNGSKNDNTTIPAQELCQKQSILGDMLRNNPFVMEMQWWVLVAITIVEIFRKLYGITGYSSFRHYV

TQVENIMEWFVITSVFVISYIYTNKTYTFQNHIGAFAVLLGWTNLMLMIGQLPVFDVYVAMYTRVQGEFAKLFMAYSCML

IGFTISFCVIFPSSSSFANPFMGFITVLVMMIGEQDLSLLINDPEGKDPPFLLEVSAQITFVLFLLFVTIILMNLLVGIA

VHDIQGLKKTAGLSKLVRQTKLISYIESALFNGYLPTWLRNLLHYTALVSPQAYRVVLCVKPLNPSEKRLPREILMKAYE

VGKMRKHFGHTISSKNSAENYLSYKNKYNNNNGATTGYVLPDSDPDAGQFTTLTTKIDDNADRIEFLTQEIQELKQALIS

QQQQASKVIDKLLIVISNQQKQNLRK

>DmTRP

MGSNTESDAEKALGSRLDYDLMMAEEYILSDVEKNFILSCERGDLPGVKKILEEYQGTDKFNINCTDPMNRSALISAIEN

ENFDLMVILLEHNIEVGDALLHAISEEYVEAVEELLQWEETNHKEGQPYSWEAVDRSKSTFTVDITPLILAAHRNNYEIL

KILLDRGATLPMPHDVKCGCDECVTSQTTDSLRHSQSRINAYRALSASSLIALSSRDPVLTVFQLSWELKRLQAMESEFR

AEYTEMRQMVQDFGTSLLDHARTSMELEVMLNFNHEPSHDIWCLGQRQTLERLKLAIRYKQKTFVAHPNVQQLLAAIWYD

GLPGFRRKQASQQLMDVVKLGCSFPIYSLKYILAPDSEGAKFMRKPFVKFITHSCSYMFFLMLLGAASLRVVQITFELLA

FPWMLTMLEDWRKHERGSLPGPIELAIITYIMALIFEELKSLYSDGLFEYIMDLWNIVDYISNMFYVTWILCRATAWVIV

HRDLWFRGIDPYFPREHWHPFDPMLLSEGAFAAGMVFSYLKLVHIFSINPHLGPLQVSLGRMIIDIIKFFFIYTLVLFAF

GCGLNQLLWYYAELEKNKCYHLHPDVADFDDQEKACTIWRRFSNLFETSQSLFWASFGLVDLVSFDLAGIKSFTRFWALL

MFGSYSVINIIVLLNMLIAMMSNSYQIISERADTEWKFARSQLWMSYFEDGGTIPPPFNLCPNMKMLRKTLGRKRPSRTK

SFMRKSMERAQTLHDKVMKLLVRRYITAEQRRRDDYGITEDDIIEVRQDISSLRFELLEIFTNNSWDVPDIEKKSQGVAR

TTKGKVMERRILKDFQIGFVENLKQEMSESESGRDIFSSLAKVIGRKKTQKGDKDWNAIARKNTFASDPIGSKRSSMQRH

SQRSLRRKIIEQANEGLQMNQTQLIEFNPNLGDVTRATRVAYVKFMRKKMAADEVSLADDEGAPNGEGEKKPLDASGSKK

SITSGGTGGGASMLAAAALRASVKNVDEKSGADGKPGTMGKPTDDKKPGDDKDKQQPPKDSKPSAGGPKPGDQKPTPGAG

APKPQAAGTISKPGESQKKDAPAPPTKPGDTKPAAPKPGESAKPEAAAKKEESSKTEASKPAATNGAAKSAAPSAPSDAK

PDSKLKPGAAGAPEATKATNGASKPDEKKSGPEEPKKAAGDSKPGDDAKDKDKKPGDDKDKKPGDDKDKKPADNNDKKPA

DDKDKKPGDDKDKKPGDDKDKKPSDDKDKKPADDKDKKPAAAPLKPAIKVGQSSAAAGGERGKSTVTGRMISGWL

>DmTRPgamma

MMEEENTIRPHQEIRQLTLEEKKFLLAVERGDMAGTRRMLQKAQDTEYINVNCVDPLGRTALLMAIDNENLEMVELLINY

NVDTKDALLHSISEEFVEAVEVLLDHENVTFHSEGNHSWESASEDTSTFTPDITPLILAAHRDNYEIIKILLDRGAVLPM

PHDVRCGCDECVQSRQEDSLRHSRSRINAYRALAGPSLIALSSKDPILTAFELSWELRRLSLLEHEFKNEYQELRKQCQD

FATALLDHTRTSHELEILLNHDPTGPVYEHGERMHLNRLKLAIKLRQKKFVAHSNVQQLLASIWYEGLPGFRRKNMALQA

VDIIRIGIMFPIFSLAYILAPYSSIGQTMRKPFIKFICHSASYFTFLFLLMLASQRIETFIGGWFFADSSGMLNTMEELP

TKRGAKPTFIEWLILAWVSGLIWSEVKQLWDVGLQEYLNDMWNVIDFVTNSLYVATVALRVVSFFQVQKEMIYNSHATDL

PRERWDAWDPMLISEGLFSAANIFSSLKLVYIFSVNPHLGPLQVSLSRMVMDIMKFFFLYVLVLFAFGSGLNQLLWYYAD

LEKKRCPEVSPMSALLNMNGTNDPNACIVWRRFSNLFETTQTLFWAVFGLIDLDSFELDGIKIFTRFWGMLMFGTYSVIN

IVVLLNLLIAMMNHSYQLISERADVEWKFARSKLWISYFEEGGTCPPPFNIIPTPKSIWYAIKWMRRVFCSGSSAARREH

LKTIRRKAQQASDRDFKYQQIMRNLVRRYVTVEQRKAESQGVTEDDVNEIKQDISAFRCELVEILKNSGMDTNVTAGQGG

GGGGKKNRQKERRLMKGFNIAPPGSTGSLAPVAEFSTSLDNYDNQHEILSSTLSTLFTPNFMHKRQQSQAGSGGGGSESP

TTPTAPQGTQGAAMTASSQVTKYNKSALKPYNKRIAGHKKRWGTLIEAAKVGNVSKMLGRSKSEDSVCNSSHTSTPVHGQ

MRVTYAQNSPQQEYGYHGETSSTTISTPTPTISVVSNSPAAHAGVGSHFFHTTSGLTAIAALKRKRKKFSSSKNICPVTE

SVAAANAAEILNNKTLKRVSSYPAAEAGVQHNPAQLVKPRRHEQTQSQHDSVETNSTFTLSIDPSNTSVNSREPLISTSC

VSTTGAIG

>DmTRPL

MGRKKKLPTGVSSGVSHASSAPKSVGGCCVPLGLPQPLLLEEKKFLLAVERGDMPNVRRILQKALRHQHININCMDPLGR

RALTLAIDNENLEMVELLVVMGVETKDALLHAINAEFVEAVELLLEHEELIYKEGEPYSWQKVDINTAMFAPDITPLMLA

AHKNNFEILRILLDRGAAVPVPHDIRCGCEECVRLTAEDSLRHSLSRVNIYRALCSPSLICLTSNDPIITAFQLSWELRN

LALTEQECKSEYMDLRRQCQKFAVDLLDQTRTSNELAIILNYDPQMSSYEPGDRMSLTRLVQAISYKQKKFVAHSNIQQL

LSSIWYDGLPGFRRKSIVDKVICIAQVAVLFPLYCLIYMCAPNCRTGQLMRKPFMKFLIHASSYLFFLFILILVSQRADD

DFVRIFGTTRMKKELAEQELRQRGQTPSKLELIVVMYVIGFVWEEVQEIFAVGMKSYLRNMWNFIDFLRNSLYVSVMCLR

AFAYIQQATEIARDPQMAYIPREKWHDFDPQLIAEGLFAAANVFSALKLVHLFSINPHLGPLQISLGRMVIDIVKFFFIY

TLVLFAFACGLNQLLWYFAALEKSKCYVLPGGEADWGSHGDSCMKWRRFGNLFESSQSLFWASFGMVGLDDFELSGIKSY

TRFWGLLMFGSYSVINVIVLLNLLIAMMSNSYAMIDEHSDTEWKFARTKLWMSYFEDSATLPPPFNVLPSVKWVIRIFRK

SSKTIDRQRSKKRKEQEQFSEYDNIMRSLVWRYVAAMHRKFENNPVSEDDINEVKSEINTMRYEMLEIFENSGMDVSSAN

KKERQPRPRRIKVWERRLMKGFQVAPVQNGCELDAFGNVNGQGEMQEIKVESIPSKPAKETAKERFQRVARTVLLQSTTH

KWNVVLRAAKDSQIGRCTKNERKSLQNLGRAIEEAKRLIMLNPGCPSGRESPIRIEFEDEKTSTLLELLNQISAEISDSE

KPKIRPIWRPPLKTVPARAMAANNTRSLTAPELKISRKSSPAPTPTPTPGVSHTALSQFRNRELPLCPSKLIANSAPSAP

TAPPKKSAPTAPTPTYKPTTHAPFSVEGGNRENTRASDGVRSDNSNFDIHVVDLDEKGGHLGRDNVSDISSIASTSPQRP

KHRN

>DmPain

MDFNNCGFIDPQAQLAGALAKQDIRQFVAALDSGALADLQDDRHTSIYEKALSTPGCRDFIEACIDHGSQVNYINKKLDK

AAISYAADSRDPGNLAALLKYRPGNKVQVDRKYGQLTPLNSLAKNLTDENAPDVYSCMQLLLDYGASPNIVDQGEFTPLH

HVLRKSKVKAGKKELIQLFLDHPELDIDSYRNGEVRRLLQAQFPELKLPEERHTGPEIDIQTLQRTLRDGDETLFEQQFA

EYLQNLKGGADNQLNAHQEEYFGLLQESIKRGRQRAFDVILSTGMDINSRPGRANEANLVETAVIYGNWQALERLLKEPN

LRLTPDSKLLNAVIGRLDEPPYDGSSHQRCFELLINSDRVDINEADSGRLVPLFFAVKYRNTSAMQKLLKNGAYIGSKSA

FGTLPIKDMPPEVLEEHFDSCITTNGERPGDQNFEIIIDYKNLMRQERDSGLNQLQDEMAPIAFIAESKEMRHLLQHPLI

SSFLFLKWHRLSVIFYLNFLIYSLFTASIITYTLLKFHESDQRALTAFFGLLSWLGISYLILRECIQWIMSPVRYFWSIT

NIMEVALITLSIFTCMESSFDKETQRVLAVFTILLVSMEFCLLVGSLPVLSISTHMLMLREVSNSFLKSFTLYSIFVLTF

SLCFYILFGKSVEEDQSKSATPCPPLGKKEGKDEEQGFNTFTKPIEAVIKTIVMLTGEFDAGSIQFTSIYTYLIFLLFVI

FMTIVLFNLLNGLAVSDTQVIKAQAELNGAICRTNVLSRYEQVLTGHGRAGFLLGNHLFRSICQRLMNIYPNYLSLRQIS

VLPNDGNKVLIPMSDPFEMRTLKKASFQQLPLSAAVPQKKLLDPPLRLLPCCCSLLTGKCSQMSGRVVKRALEVIDQKNA

AEQRRKQEQINDSRLKLIEYKLEQLIQLVQDRK

>DmNan

MGNTESNVTSGVKKQAGVSTQALYKFVNLKGGGLLVDMMKRACQTKQFAEIDHAIKTKVEPFLYNKGAGRYFPISKLVLL

RNRDRPRTRQLPEIRALENPDDDFNIHDYCPEVSEAEYISNPTAYRFVCWDLNMRGAVGETILHLCLLNASSLHADLAKR

LLKFYPKLILDIYMSDEYYGESVLHIAIVNEDPAMVKYLLDANADVQERCCGAFMSAEDTKFSRTDSPDHEYVALCPMTN

YDGYVYWGEYPLSFAACLSQEECFRLVLARGADPDFQDTNGNTVLHMLVIYEKIEMFDVGYEVGTNIHIKNIQNLTPLTL

AAKLGRVEMFFHVMSIEREIYWQLGSITCAAYPLLMIDTINEETGNINKDSVLNFVVFGDKLEHLELLDGVVIDLLKTKW

DTFCKSRFYKQFYMFALYFLISLFSFILRPGPDAKDEDEDGANSTTAKSDLYRQNGSDSYHLHSKRATMTTEYKTFWLNF

TEYYDPSEVEVLPAWWESYAQCPLMNLESDLAKLRIMAELLNFVGAILYLLVALREARFLGLKMFIENLMTAPSRVMFLF

SCALMMTIPWLRVSCLTEIDDHVTVVIMLTTAPYFLFFCRGFKTVGPFVVMIYRMVMGDLLRFVSIYLVFVMGFSQAFYI

IFLTFDNPSSPEDQDAESNPMPSPMESIVAMFLMSLTNFGDYYGAMVSTQHEYEAKILFFLFMVIVSVLLVNMLIAMMGN

TYQKIAEIRNEWQRQWARIVLVVERSVPPAERLKNFMQYSQSMSDGRRALVLRLNMTEEKEEMKEVQEMKRIHQRFAKKR

QMEREARALRRQQEYEKFFGTAPKSECSDNNNF

>DmIav

MKFLLKKCLRKKAPEMKPGAILDAVISQSSATACKCLLYKLADYKRGGDLIDAINSGGLIAVEQLIREQFGVFMYNDGKG

QVINRAEFLRWKYRDHTEVTIPIEASLSIHDPLGKWEDHKACWQMQYRGALGESLLHVLIICDSKVHTKLARVLLRVFPN

LALDVMEGEEYLGASALHLSIAYSNNELVADLIEAGADIHQRAIGSFFLPRDQQRANPAKSTDYEGLAYMGEYPLAWAAC

CANESVYNLLVDCGSDPDAQDSFGNMILHMVVVCDKLDMFGYALRHPKTPAKNGIVNQTGLTPLTLACKLGRAEVFREML

ELSAREFWRYSNITCSGYPLNALDTLLPDGRTNWNSALFIILNGTKPEHLDMLDGGIIQRLLEEKWKTFAQNQFLKRLLI

LSTHLLCLSVSVYLRPAHDGEAEDEDSEGSDASAAALLDIQSDEGDSGGGDYNAQTVARYCAEFATLVGVLSYVIFQQGD

EIKNQGLSAFLKQLSHAPAKAIFLFSNLLILACIPFRLIGDTDTEEAILIFAVPGSWFLLMFFAGAIRLTGPFVTMIYSM

ITGDMFTFGIIYCIVLCGFSQAFYFLYKGHPQVQSTMFNTYTSTWMALFQTTLGDYNYPDLNQTTYPNLSKTVFVIFMIF

VPILLLNMLIAMMGNTYVTVIEQSEKEWMKQWAKIVVTLERAVPQADAKGYLEAYSIPLGPSDDSGFEVRGVMVIKSKSK

TRAKQRKGAVSNWKRVGRVTLTALKKRGMTGEEMRRLMWGRASISSPVKVTKQKLKDPYNLHTDSDFTNAMDMLTFASNP

ASSNGVTLRSVTAPPPAPPAPDPFRELIMMSDQRPETHDPHYFAGLQQLANKAFDLVEQTMKTQPQAPVAKKVDPLPVAS

VAKASPAAPATQATATAAAASDLMAMPLPISNLSNLFQDPKDIVDPKKLEEFMAMLAEVETEESDSGGPILGKLSLAKRT

HNALSKAEIRRDQQGFEGHSHGQFQPMSSVWAPPGLDVDTGFHFDEAVAEEVLTIEQEAEVETEDGNGGQDSEDIPTAEE

VHATMKQFHLRKCQPAQDEAARRAKSARVRRRNKVSPEQSDDPDERSQRGRSAYTRRTQSPPDPLEPWSTRELQDINKIL

ARK

>DmNompC

MSQPRGGRGGGRGGGVGRKTPSSLTGPPDESATPSERATPASKADSDPKDDSSSNGDKKDMDLFPAPKPPSAGASIRDTA

NKVLGLAMKSEWTPIEAELKKLEKYVANVGEDGNHIPLAGVHDMNTGMTPLMYATKDNKTAIMDRMIELGADVGARNNDN

YNVLHIAAMYSREDVVKLLLTKRGVDPFSTGGSRSQTAVHLVSSRQTGTATNILRALLAAAGKDIRLKADGRGKIPLLLA

VESGNQSMCRELLAAQTAEQLKATTANGDTALHLAARRRDVDMVRILVDYGTNVDTQNGEGQTPLHIAAAEGDEALLKYF

YGVRASASIADNQDRTPMHLAAENGHAHVIEILADKFKASIFERTKDGSTLMHIASLNGHAECATMLFKKGVYLHMPNKD

GARSIHTAAAYGHTGIINTLLQKGEKVDVTTNDNYTALHIAVESAKPAVVETLLGFGADVHVRGGKLRETPLHIAARVKD

GDRCALMLLKSGASPNLTTDDCLTPVHVAARHGNLATLMQLLEDEGDPLYKSNTGETPLHMACRACHPDIVRHLIETVKE

KHGPDKATTYINSVNEDGATALHYTCQITKEEVKIPESDKQIVRMLLENGADVTLQTKTALETAFHYCAVAGNNDVLMEM

ISHMNPTDIQKAMNRQSSVGWTPLLIACHRGHMELVNNLLANHARVDVFDTEGRSALHLAAERGYLHVCDALLTNKAFIN

SKSRVGRTALHLAAMNGFTHLVKFLIKDHNAVIDILTLRKQTPLHLAAASGQMEVCQLLLELGANIDATDDLGQKPIHVA

AQNNYSEVAKLFLQQHPSLVNATSKDGNTCAHIAAMQGSVKVIEELMKFDRSGVISARNKLTDATPLQLAAEGGHADVVK

ALVRAGASCTEENKAGFTAVHLAAQNGHGQVLDVLKSTNSLRINSKKLGLTPLHVAAYYGQADTVRELLTSVPATVKSET

PTGQSLFGDLGTESGMTPLHLAAFSGNENVVRLLLNSAGVQVDAATIENGYNPLHLACFGGHMSVVGLLLSRSAELLQSQ

DRNGRTGLHIAAMHGHIQMVEILLGQGAEINATDRNGWTPLHCAAKAGHLEVVKLLCEAGASPKSETNYGCAAIWFAASE

GHNEVLRYLMNKEHDTYGLMEDKRFVYNLMVVSKNHNNKPIQEFVLVSPAPVDTAAKLSNIYIVLSTKEKERAKDLVAAG

KQCEAMATELLALAAGSDSAGKILQATDKRNVEFLDVLIENEQKEVIAHTVVQRYLQELWHGSLTWASWKILLLLVAFIV

CPPVWIGFTFPMGHKFNKVPIIKFMSYLTSHIYLMIHLSIVGITPIYPVLRLSLVPYWYEVGLLIWLSGLLLFELTNPSD

KSGLGSIKVLVLLLGMAGVGVHVSAFLFVSKEYWPTLVYCRNQCFALAFLLACVQILDFLSFHHLFGPWAIIIGDLLKDL

ARFLAVLAIFVFGFSMHIVALNQSFANFSPEDLRSFEKKNRNRGYFSDVRMHPINSFELLFFAVFGQTTTEQTQVDKIKN

VATPTQPYWVEYLFKIVFGIYMLVSVVVLINLLIAMMSDTYQRIQVVLLNALLSNSTLFINSYFNHKYINFILHCVLIIL

YFSIRSKFTYEDDLYFLDI

>DmTRPM

MYFETNWVFHQPRSWIETNFQKRECIKFIPCPKDDTKCCCGQAQITHQTIPGIESGSPGDLWLPTKHTRPQPTDAYGTIE

FQGGAHPTKAQYVRLSFDTRPELLVQLFTKEWNLELPKLLITVQGGKANFDLQAKLKKEIRKGLLKAAKTTGAWIFTGGT

NTGVTKQVGDALLLEGQQRTGRVVSIGIAPWGIVERNHELLGHNREVPCHSISSPRSKLAVLNNRHAYFLLVDNGTQAKY

GAELILRRKLEKFISNLKLHPFTHSSTPVVCLVIEGGTNTIRAVLEYVTDSPPVPVVVCDGSGRAADLLAFVHKYASDGE

EQPVLESMRDYLIGTIQKTFEVGLDQSEKLYQELLQCTRNKNLITVFRIQEKPEGEAQELDQTILTALFKSQHLSPPEQL

SLALTWNRVDIARSEIFVYGQEWPNGALDEAMMQALEHDRIDFVKLLLENGVSMKKFLTIPRLEELYNTKHGPANTLGYI

LRDVRPHIPKGYIYTLHDIGLVINKLMGGAYRSYYTRRKFRPIYAKVMNSYANACRKSSTYQYQRYAGANSLSLVTGLLP

FTSEMALFEFPFNELLIWAVLTKRQQMALLMWTHGEEALAKSLVSCKLYKAMAHEAAEDDLDTEIYEELRSYAKEFESKG

NKLLDFSYRQDAEKAQRLLTCELHSWSNQSCLSLAVAANHRALLAHPCSQVILADLWMGGLRTRKNTNFKVILGLAMPFY

IRQLDFKSKEELQQMPQTEEEHLENQNLDNDDSDRSQPDAEALLADTYSVRDTKVHENGKVSLTDSDTAQFREFFNLSEY

NEVKQHQPLRLKKKFYEFYTAPITKFWADSIAYMFFLIMFSFTVLVKMEQMPRWQEWYSIAYITTLGFEKVREIISSEPV

AITHKFSVWAWNMWNPCDGAAIILFVIGLAFRFRENTMDIGRVIYCVDSIYWYLRILNILGVNKYLGPLVTMMGKMVKNM

IYFVVLLAVVLMSFGVSRQAILYPNKQPTWSLIKEVTFQPYFMLYGEVFAGDIDPPCGEDPSQPGCVTGHWVTPITMSMY

LLIANILLINLLIAVFNNIFNEVNSVSHQVWMFQRFTVVMEYQQKPVLPPPFIALCHFYSLLKYCVRKAKGEPGMSKFSF

VPCESIVSDAL

>DmTRPML

MQSYGPGAQTAPAVKRRTDSYEAAQQQQQSPESDEEYVNTRILRRQVQLQSTPVAPVVPMPISAGSGTAPPSVDGREEQP

EFPGSSAASYQEERMRRKLQFFFMNPIEKWQAKRKFPYKFVVQIVKIFLVTMQLCLFAHSRYNHINYTGDNRFAFSHLFL

RGWDSSREVESYPPAVGPFALYLKSEFFDTVQYAVNGYANVSRSIGPYDYPTPNNTMPPLKLCLQNYREGTIFGFNESYI

FDPHIDEVCERLPPNVTTIGVENYLRQRDVEVNFASLVSAQLTFKIKTVNFKANGGPLSAPDCFRFDISITFNNRDHDGQ

MLLSLDAEATRLKCHGATDFISDANFDSMLRSVLNIFVLLTCALSFALCTRALWRAYLLRCTTVNFFRSQFGKELSFDGR

LEFVNFWYIMIIFNDVLLIIGSALKEQIEGRYLVVDQWDTCSLFLGIGNLLVWFGVLRYLGFFKTYNVVILTLKKAAPKI

LRFLIAALLIYAGFVFCGWLILGPYHMKFRSLATTSECLFALINGDDMFATFATLSSKATWLWWFCQIYLYSFISLYIYV

VLSLFIAVIMDAYDTIKAYYKDGFPTTDLKAFVGTRTAEDISSGVFMTDLDDFDQTSFLDVVKSICCCGRCGRHQEPAQP

NSGYTSLSSIMK

>BmTRPM

MALLLPITVFRIQERAEGGEVQELDQVILTALFRAQHLTPSEQLSLALTWNRVDIARSEIFVYGQEWPPG

ALDEAMMQALEHDRIDFVKLLLENGVSMRKFLTIPRLEELYNTKSGPSNTLRYILRDVRPHLPKGYVYTL

HDIGLVINKLIGGAYRCYYTRRKFRPIYAKVMNKSVNVHRNSASFTRHNAGGLSLITGFMPVTSEMALFD

YPFNELLMWAVLTKRHQMALLMWTHGEESLAKSLIACKLYKAMAHEAAEDDMETEVYEELRHYGKEFENK

ALELLDYCYRQDDDQAQQLLTCELQNWSGQTCLSLAVTANHRALLAHPCSQIILADLWMGGLRTRKNTNL

KVILSLLCPLYILRLEFKSKEELQLMPQTEEEHLENESIEDDRSTKDPTDAEALIGSGECETRVDQNGKV

GKVGWEPSYRELPEYHPSEIKRMRPLRLRKKIYEFFTAPITKFWADSIAYILFLLMFTYTVLVKMNPTPS

WPEIYSICYILTFLCEKIREIVTSEPVAIRHKFSVWAWNMWNTYDAGFIIFFLVGLTLRLREISRDVGRV

IYCVDIIYWYLRILNILGVNKYLGPLVTMMGKMVKNMIYFVVLLLVVLMSFGVARQAILHPDKDASWHLI

REVFFQPYFMLYGEVFAEQMAPPCGLPGLEECRVGHWVPPLVMSVYLLIANILLVNLLIAVFNNIFIEVN

GVSHQVFYQPYFMLYGEVFAGDIDPPCGKEIGDRKCVTGRWITPIAMTVYLLIANILLINLLIAVFNNIF

NEVNEVSHQVWMFQRFTVVMEYEQKPVLPPPLIIFCHIYAVGKWVTRNVSHKRFQYDNGLKLFLEKEDME

RLYDFEEECVEGYFREQEIKLSNSIEERVRNTTDRVEHITTKMEDLNQKANCQIQSMQSVEFRLRKLEDI

AEQTMSHLAVIHRFMATHPSLVNRGSTDTLGPPPPSFQAGGNRLRTASDRSEVLSDSDTGARFAVRPVQE

EDEQPSCHSDVSHSDTLKLRDRCLNNRSFKSRLWKRERERERERERERERERESERERARERERERERER

ESERERARERERERESRERERERRSEGGNDCGMGPYSSQHLAPRRQHSQTHSEPDNSAVDAGSVHNSVTG

PQSGWEGAGATGAAGGTGRARVGGAPRSLLLAMHEYTSITDELETVYGLFSPPHTPRTPITSRLLSPARA

ASPSVRRRHASEMSNPEFALFMEKEHLRGAEEDDYIIMENLIQRRLEMGSVLDRYEEEEGGEGAGAGGAI

SISVCVNTSEEQPQSSSLLTVTDRRLPHQRHSLRRSSAVDSRELPSPLQPLLGDDACCEVLLPVPRQPSG

DTNASSDLSLSHMVSENLPPRPSIVLDSSQLPRQRSAEQQQTQPPGPSRPETMC

>BmWtrw

MLHESESAAELNCILSACISVKFNVHCQVYERPNNDDDKVRTIHLVIDGRSTIAAESGEQCVATSRMPNT

RRLLPARWLRARAPDEPRPADRRGRDLPDHESLERAYPSMGHLEYVLAGSSPPAESAPNMYDSFEEPPLD

LTAHICADSLRQSAHEQMRAVGGRLRLLDELESGVIPADATTDAFATATDAEKNVCLFWTAFLKLANLLP

PLIEVGAEPLYYDSLGLSPLHVAAFSGSVECASYLLSCGADPNYMPRCFAPLHCAAFGNSVQVANLLIAR

GASVHAAVKYINCEGGLLHCAVRANSAECLKLFISHGVDVNLIEPGGTNAIHLAADLGVKQCLIILLDTP

GADPNVRTRVGDRESTALHLAADGGFVECVDLLLSKGADASLKNHRGFTALHLAARSASLECVESLLRKG

NADPNAMDFDKRTPLHAAIGKSDSACDIIETLISWGANVNQKDEYGFTPLHLAALDGLSACVETLIYHGA

DVTTRSKKGNSALNVIARKTPASLAMVTRKLDCAITLHHSQSSNREVELELDFRSILQHCYPREISYLNT

FVDEGQKEVLLHPLCSAFLYIKWEKIRKYYVARLFLSFIFVLCLTLYVLTALAHNCYNGSKDMEETIQEQ

ELCQKQSILGDLLRKNPFVIEMQWWVLAGITIFEIFRKVYGIAGYSTVKQYLMQSENIIEWFVIISVFLI

SYIYTNITYTWQNHVGAFAVLAGWTNLMMMIGQLPVFGTYVAMYQKVQKEFAKLLMAYSCILIGFTISFC

VIFPDSSSFANPFMGFITVLTMMIGELNLDLLLNEPDGNDPPVLLEFSAQITYVLFLMFVTVVLMNLLVG

IAVHDIQGLRKTAGLSKLVRQTKLISYMELALFNGYLPKCLLKILHSSALVSPQAYRVVLSVKPLNPSEK

RLPRDIMMAAYDIAKMRKQYGHTISSSGSTTGAYSCFKKYESNNDSGYREYGYSGLGSIHARLDETSEHV

RQLTQEVKELKKLINAQQLVIQQALVGAMDRP

>BmWtrw2

MDNFGYREMGWPSPLQTPTRSRLSSSIRSRGREPEEFPLNPQYKHVKNIDLLESAREPGKAKEYLFVGPS

PPADDAPNMYHSFDVLAPDPDARLTDDAARRGLYERALTLGAGRFFDDLECGLITAENIEEHISSAPEAV

VNLTLLWASYLARDELLPGVLDAGADIHYSDSSGLTALHLSAFSGAGRAAVFLISRGADVDFVPKYFTPL

HCAAFGNSLEVAEILIANGASLHGVVQRAGCEDNLVHCAVRADAVECMEMFIERGVDPAYATSGGLNALH

LAAELGSRRCLSYLLRETKISVNGMSKQRDKECTALHLAASRGYVECVELLLSEGAKANTKNYRGFTALH

LAARLSSIDCVEVLLRDGNADPNAEDYDKRSPLHAAITRSERACDIIELLVNWGAQVNKKDEYGYSAIHL

AAMDGLTQCVETLIFLGADVTSKSKKGHTALSVIARKTPKSLAILKHNLDCGISLSRSIEGNEEVQIEFD

FGKLLKFSYPREITYLNSLVDEGQKDILQHPLCSAFLFMKWRKIRKFYLARLIFCFLFVSFLSIYVLTAV

VKACQGKHSKKYGVPNELCQPQSILGVILNDNPIEFERWVLMAITVFEIVRKLTGITGYSSFYQYFTTFE

NLMEWFVLLSVFSLYNIRNDYSWQNHVGGYAVLGAWTNLMLMMGQLPMFGDYVAMYQKVLMEFLKLLLAY

ICLLLGFTICFCVVFPNEEMFSNPLMGFISTLSMMVGELNLNILINDPMQDDPPIFFELSAQIIFIFFLM

FVTIILMNLLVGIAVHDIQGLRKTAGLSKLVRQTKLILFVEMGMFSAWLPKCLHKYVYRTALVSPEAGKV

ILSVKPLNPREKRLPTDIMMAAYELAQLNKVKSGRSVKEVLYKNKISSKLKNEGHNNEQNVGFEIRGMQE

KIDQATFNLKKIDQEMRHLNTLLMEQQNFFQSLFKSTELVPYKSQHASTPVYSDSPIIFGNNT

>BmIav

MGNAIGKFLTAGNVQGAGSVLDRVISQPSSEDHTVLYKLADYKKGGLLLETYAKGGMTAAEKLMRDEFAA

YMYGGGRGRVINRAEYLRWKFRDQEQVVLPIEASLSPYDPLAKWEDHTACWQMCYRGALGESLLHVLIIC

DTKIHTRLARTLVKCFPKLSLDVVEGEEYLGASSLHLAIAYSNNELVQDLVEAGADVNQRAIGSFFLPRD

QQRVPPARQTNYEGLAYLGEYPLAWTACCANEAVYNLLLDSGADPDAQDSFGNMILHMVVVCDKLDMFGY

ALRHPKVPASNGRMNKAGFTPLTLACQLGRASVFREMLELSAREFWRYSNITCSAYPLNALDTLLPDGRT

NWNSALFIILNGTKQEHLNMLDGGIIQRLLEEKWKTFARTKFLKRLLILMLHLLLLSVSVYLRHSSAEAD

AHPNWGLEINDARSGLRLASELGTILSTLCYIILQQGDELKNQGLVAYFKQLIHEPAKFIFLASNILVLA

CIPARLLKETNVEEAILLFLLPGSWFLLMFFAGAVKLTGPFVTMIYSMITGDMFTFGIIYCIVLFGFSQS

FYFLYRGFPNVQSTLYSSYPSTWMALFQITLGDYSYSDLSQTTYPNLSKTVFTVFMIFVPILLLNMLIAM

MGNTYAHVIEQSEKEWVKQWAKIVVSLERSVAQDDAHKYLQEYSIGLGPSDDPRYEQRAVMVIKSKAKTR

AKQKKDALTNWKHVGKVTIAELRRRGISGEELRRLMWGRISISTPTKAPLPRRVPAPPPDCVVSSDVGLA

SDVGNGVAPALSSALNVMAFTHELDIGTTGSDQKQTTPDLLVNGKTSNAPILTTGTQMPKVSSKTLGTPV

ARIEASSLKTPLDVKLNPTTLTQSTVQGNIGNVNIPISGVKITTNVGQNSAVVPEIQQSNENQKPEQVHQ

DYLRELIVLAEKPATTNLELKQLAEKAADLRDVPEIDININMAAKSARKMVAGAVSGLFGVAADTPAPDA

GWRRDRHDNSDSDPISGTI

>BmNan

MIGGGLLVDMMKRALQNKQYAEIDHAIKTKVEPFLYNRGRGRYIPISHLVLLRNKERPRHKLLPPLRGME

NPDEEFDVDKDWPVVTQEEYDANPSGYRELCWDVKERGAVGETILHLCLLNATSLLAHLAKRLLRFYPKM

INDIYISDEYYGETVLHITIVNEDPTMAKFLLDAGADYHERCYGNFMCPEDQKASRTDSFDHEWVNVQPD

TNYNGYVYWGEYPLSFAACLGQEECYRLILARGADPDKQDTNGNTVLHMLVIYEKMSTFDMAYEVGASLN

IRNVQNLTPLTLAAKLARTEMFFHILNIEREIYWQIGATTCAAYPLGQVDTIDTETGLISKDSALNLVVF

GEKDEHLGLLEGMLIDLLKTKWNTFVKFRFYRQFILFSCYFLVSLICFTLRPGPPDRALNTTVLNSTIGP

NVTDAELVSDVENCTMTPNADFDTNAVEVLNGTKFGGSHCARFKSHPKEKSTEAPRENDVEGWWEDLTEE

CRLMNLDTWQAKLRISAELLLWMGALAYFGAALREAKFLGIKMFIENLSTVPSRVMFLFSCLLMLILPTL

RLWCADEAEDHLAVMIMLTTAPYFLFFCRGFKTVGPFVVMIYRMVMGDLLRFVCIYLVFVMGFSQAYYVI

FLSFDNPNTPEGVDDSVSNPMPSPMESIMAMFLMSLTSFSDYYTAFDRTDHEIEAKLLFVIYMIIVAILL

VNMLIAMMGNTYQKIAETRNEWQRQWARIVLVVERGVPPAQRLKQLMTYSQPMANGKRALVLRINQKDED

KEEMKEILEMKRTHERIVAKRKQREALKPGEIPPPARDYPIRK

>BmNompC

MSDSGRDVPRSANINFSSGVRSPPANQQKDLQRLELSFIELNKERAKQKEWKKLIRDLVFQRGKVPLLLA

VEAGNQSMVRELLSAQTAEQLKATTPAGDTALHLAARRRDVDMARILVDYGAVVDATNGAGQTPLHIAAA

EGDEPLVKYFYGVRANAAIADNEDRTPMHLAAENGHAAIIELLADKFKASIFERTKDGSTLMHIASLNGH

ADCAMMLFKKGVYLHMPNKYFEKQTFDEVLFVKIENGLIKGLKSDDGSYSMFLGIPYALVNESNPFGDSL

PTLPFNEIYEAFDDSTACPQKEEFNNTIVGEMQCLRLNIYVPKSLNNNLPVLIYFYGGTFEIGFAGRYLY

GPKYLVRHEIILVTVNYRLGPYGFLCTDSSNAIGFKTAQLDLAIDFLSKENYKDVINAAVELDVKFYCCN

ENYFSGAQNFLTDYPINLNTPIVEGMSILIGNTNNERAHNYYGKKNLNFDIFQDHLQIAFNFDDEHLLNT

KEIVKKQYIGNEKNIDMVENNIVQFSSDFYYYHPTKRNVLKYIDNGAKKVYNYLFSYDGNRNFLKKTFNL

NGSGAFHADEIGYLFDISFMDKTLTIEDMLIVDRITTLWANFVKSGNPTPVPTELLPIIWKPISKDSFYT

LNIDTAMELKSGFFEDRMIFWDTFFEKYKHFDGARSIHTAARYGHVGIINTLLQKGESVDVTTNDNYTAL

HIAVESCKPAVVETLLGYGADVHIRGGKQRETPLHIAARIPDGDKCALMLLKSGAGPNKATEDGMTPVHV

AAKFGNLATLVLLLEDGGDPLRKTKVCIYDVVTGETPLQMACRSCMPDIVRHLIEFVKDHKGESVSTAFI

DAVDEDGASALHYACKVTKEEVKIPTADRQVVKCLIENGADVSLQTRHNHETAFHYCAIAGNNDVMTEMI

AHISTADVSRALNRQNSIGWTPLLIACHRGHMQLVNTLLTNHARVDVFDVEGRSALHLAAEHGYLQVCDA

LLTNKAFINSKARNGRTALHLAAMNGYAHLVKFLIRDHNAMIDVLTLKKQTPLHLAAASGQIEVCKLLLE

LGANIDATDELGQKPIHAAAQNNYSEVVQLFLQQHPNLVMATTKDGNTCAHIAAIQGSVKVIEELMKFDR

TGVISARNKLNDSTPLQLAAEGGHADVVRVLVRAGASCTDENRAGLTAVHLAAEHGHTNVLDVMRSTNTL

RISSKKLGLTPLHIAAYYGQAETVRELLSHVPGTVKSEAPTGVSLVPVLGAESGLTPLHLAAYNGNENVV

RLLLNSAGVQADAASNENGYNPLHLACFGGHMSIVGLLLSRSAELLHSTDRHGKTGLHIASTHGHYQMVE

VLLGQGAEINATDKNGWTPLHCAAKAGHLNVVKLLCESGASPKSETNLNCAPIWFAASENHNDVLEYLLH

KEHDTQSLMDDKRFVYNLMVCSKNHNNIPIEEFVLVSPAPVDTAAKLSNIYINLSTKEKERAKDLIAAGK

QCEAMATELLALAAGADSAGHILTATDNRNIEFLDVLIENEQKEVIAHTVVQRYLQELWRGSLKWTGIKI

MFLFFAFIVCPPVWMVFSLPLGHRYHKIPIIKFMSYLTAHIYLMVLLALVAITPIYNSIFRDSLIPRWYE

WMLLISLSGLLLLELTNPSDKSGLGWIKIAVLLLGMIGVATHVVGWIFIQPKYWPTLMYCRNQCFALSFL

LACVQILDFLSFHHLFGPWAIIIGDLMKDLGRFLAVLAIFVFGFSMHIVALNQPFRNINKSEDNKYASQA

RRKLFSDENQRNKRQAFVPQWIKPLSGESYRRKQGPAGPTLTPIEAFEKLFFALFGQITLSDLNYISNLR

PSWTSNLFKFVFGAYLLMSVVVLITLLIAMMSDTYQRIQLLFFAVFGQTTTEQTKVHSKDSNIQPAWTNY

LFKIVFGIYMLVSVVVLINLLIAMMSDTYQRIQAQSDIEWKYGLSKLIRNMHRTNTAPSPLNLVTTWLMW

LIARCRERLTKKKRPSLVHMIGLQRQDQLSARSKAGAKWLSKVKRGQVVPKDSTRLSVVHLSPLGSQLSF

NNATRIENVVDWEIIAKKYRALMRDEPEESTAKESETDSSDEVSEIIPNNIAAAPP

>BmPain

MSRDNYEMKPKQLSRNSSLFGTDPQELLNKALQNNDYAKFKKLVTEANVDLEHVYEYPDYKTCLEVAVSD

RNKLEFVKLLLQYQVEVNKVNVTHSAAPIHFAVENGNIDALAALLEDDRIDVNVKSRGNTAILMAVKQIE

ELDDAREHELTIYEDMIELLLKAGCNANSPDLKGVTPIYSAAKQGLERVITLIIDYAKHAIDIDTYKDRR

GKTARDYLKEAFPYLEAKFDSTTQDPEIVDSDKLFSYLSRHEEDNFIRDFLKLANKNEHRKILPVNNGMN

TMLQLATEKGFEKAVSTLLRYGADPNATCSSNTSRPIALACQNGYYKIVKMFMDNESTLFDPVNSESLVQ

ITIKGRRSSINIPNVNFDECLRLLLNHPKMDINVNHMDMKNNTALHYAARNGDNKTVLELLRNGACIGLR

NSFDEPPLADINAKTLEAFLDECVTTNNERPSNDDYEIHMKYSFLVYPNNSLENELCKVPLIDNTNNNVK

EYDTILAPETDALLYMTRNEELRPLLKHPVITSFLYLKWQRISCLFFANITFYSFLWLCLILYIILGYGA

EKKQRDSFEALNVITHVGVIIGVVLLLIRELFQLLLSPTRYLQSIENWMEIGLIFVTIWIVFSKSATEST

KQQLSAVAILLSSAELVLLIGQFPTLSTNIVMLKTVSWNFFKFLLWYCILIIAFALSFYTLFRQENEEDQ

RAPDPNTVGKEEEEEDFFEDPGRSLFKTIVMLTGEFDASSIKFSTYPLTSHIIFIVFVFMIPIVLFNLLN

GLAVSDTQEIRADAELVGHISRIKLISYIESVLIGSAKTHSRPSKCWSLLPFNIQNLNLIKPKTFFTKSF

AKRICLFPHFLPKYKILVKPNQNNIIEIPHADDKDLERSGGCCFERCQNYRLDRKIVKNAKLVISNKTKI

TEFDDKLSMYENKIESLEATLKKVLLAIESQRN

>BGIBMGA009272-PA

MQKTFSRRLIEQNLGKPEVLDINCVDPLNRSALIAAIENENIELIKLLLGSGIKVKDALLHAIKEEYVEA

VELLLQWEEEHYEPGEPYSWESVDSDGATFTPDITPLILAAHRNHYEILKILLDRGATLPVPHDVKCGCD

ECVKSSQEDSLRHSQARINAYRALSSPSLIALSSADPLLTAFQLSWELGRLSRMETEFRVEYKALRQQCQ

EFATSLLDHTRTSKELEIMLNYNPWDLDCWEPGERQTLGRLKLAIKYKQKMFVAHPNVQQLLGAIWYEGL

PGFKRKNIFGQCVQVAKLGVMFPVYCTIYMLAPNSEYGRFMKKPFVKFICHSSSYMLFLTLLSLASQRAE

YVVLEWSNVSWLQELVGYWKDHERGSLPGVIEFAVIIYIASLIWAEIRALWTGGLTEYISDLWNIVDFIT

NMFYIAWISLRISSWYIVQRDHHSGLDPWYPRERWDSYDPMLLSEGAFAAGMIFSFLKLVHIFSINPYLG

PLQVSLGRMILDILKFFFVYMLVLFAFGCGLNQLMWYYAELEKDKCYHLPNGLPDFDGQERACSIWRRYA

NLCETSQSLFWASFGLVDLTTFELTGIKSFTRFWALLTFGSYSVINIIVLLNMLIAMMSNSYQIISERAD

TEWKFARSNLWMSYFEDGDTVPPPFNIIPTPKHFFCWIKSYFNKRERRSILNKSREKARQEHEAVMRILV

RRYVTAEQRARDEVGVTEDDVMEIRQDISTLRYELIDILHNNGMKTPRVSLQDTAVSGKKGKVMERRILK

DFQIGIVEGIIKDVISKENKPKDVFSQIAKAIVRRSSTDSKKRDWNALVRTNTVRRDPIGTAAEAEIRRS

RQSLRAHILENVQSRTIDPQKLLQYNPKLSELTPTTRVAYAKFMRSKLRSDFSAKERNKRKSDESSLDGE

DFNDEVLEAPQRHDQKRPVRSFRSRTPIPEMSDDEGLRENEITIEVDVEDQDTDSIKTKPLKIYSGDHTE

PNTPSMDGNYHHFPSPESQDHVIQIEPDTPTKNLLLMKEEERNLQDPTAKSPSLKQNEQILSASALESQY

TQASSSRTSITKGGTEKLPYKSCLKYTNDTPKNVESAIPSTSRLSRKDSKLVSASLQSQMAPSRFSQSNV

TKSPDKSLASSKTSSPKSLSPRSPRVVPSSPTPSIMSAGKGKSKATGRIVSGWL

>BGIBMGA001085-PA

MSGGSGGGGSSADGRRPSTCRVDMGALLASEPRPLDSKVKRHSIHGMTEEENVVRPHQEMAVLSLEEKKY

LLGVERGDVAGTRRVLQRARDTGHINVDCVDPLGRSALLMAIDNENLEMVELLLEFGVETRDALLHAISE

EFVEAVEALLDHEERTRKPGEPNSWEALPPETATFTSDITPLILAAHRDSYEIIKLLLDRGAQLPEPHDV

RCGCDDCVRSRREDSLRHSRSRINAYRALASPSLIALSSKDPILTAFELSWELRRLSALEHEFKTEYQEL

RVQCQEFATALLDHTRTSNELQILLNHEKGASPQAPLTEPGAPERMRLSRLKLAIKLRQKKFVAHPNVQQ

LLASIWYESVPGFRRKNMLLQAAEMVRIGAMFPLYSLAYIAAPHSAVGRTLRKPFIKFLCHSASYFMFLF

LLILASQRIETAPGGLLWDVSHDEPLSRRGSMPSIVEWLILAWVSGLIWSEVKQLWDMGLREYVHDMWNV

IDFVTNSLYVATVALRIVSHYQVRREMAMGLQWNQPREKWDAWDPMLLSEGLFSAANIFSSLKLVYIFSV

NPHLGPLQVSLSRMVLDILKFFVLDILVIFAFSCGLNQLLWYYADMEKKRCTVGTALTPNGTLPDPDACV

VWRRLFETMQTLFWAAFGLVDLDSFELDGIKIFTRFWGMLMFGTYAVINVIVLLNLLIAMMNHSYQLISE

RADVEWKFARSKLWISYFEEGGTAPPPFNVLPSPKSLLYAWRWLQRRLCGHARAKREHMRTIRAIMRNLV

RRYVTVQQRRAESGGVTEDDVNEIKQDVSAFRCELVEILRNSGMNTSTANAGAPEVMMLRAGGGGGKKNR

QKERRLMKGFNIAPGGSLAPVDEFMSPVSWLQHDHGVPHYSLSTLLGPRLRASQSSLSDGPGAGMSASRR

KPQHKRRWGTIIDAARAARVSRLIGRSRSEDSVCDHARQSPSGSEPSESGSDSQRSPERTGSRSGPLHPL

TALAALKRKRKKFSDSRRPEATVRPVPAPESLQRASSVVLPVKF

>BGIBMGA009273-PA

MAEKKDLEAGDPELECVRKPMPLPVLPKPLTLEEKKYLLAVERGDMANVRRLLQKGHRKKHIDINCVDSL

GRGALTLSIDGENLEMVELLVIMGVETRDALLQAINAEFVEAVELLLEHEELIHKDGEPYSWQKVDPNTA

MFTPDITPLMLAAHKNNYEIIKILLDRGATLPNPHDVRCGCEDCIRQSTEDSLRHSLARLNEYRALASPS

LIALSSTDPILTAFELSWELRNLAFAEQESKAEYLELRRQAQLFAVDLLDQSRSSQELAIILNHDPDEPA

FVDGEHMKLARLELAIDFKQKKFVAHPNIQQLLASIWYEGVPGFRRKTTMEKIMIICRVALLFPFYCTLY

MIAPNCATGKLMRKPFMKFLIHASSYLFFLLILILVSQRAEVQAIQLFGPEWMVKELEKELLKQRGNGPT

YLELVVVVYVLGFIWEETQEIYIEGIRSYLRNMWNFIDFTRNSLYVAVALLRFAAYLQQAAEIRRDQQTK

FIPRESWDAFDPQLIAEGLFAAANIFSALKLVHLFSINPHLGPLQISLGRMVIDIVKFFFIYSLVLFAFA

CGLNQLLWYFADLEKKKCYVLPGGLPDWDNAGDSCMKWRSFGK

>BmPyr

MSQLQEDAEDDVEGGYISSDESAHGADLAERLRVKPSKDIWELDEIQYTLRLLPYSEEISVLIGNGNYDE

TIQFASEAEGGFGLRTAILWACWLGKSILLFKLLKMGVDPDELDDAGRTCLHLSCLVGSEECVKLLLDHG

AHPNTWDSSTETKATPLHCAASAKSLACVKVLIAHGADVNAGLSDRSPLHYAVLSDAPEVVKELLEAGAC

PDTPQVFTETPLHVAASLGSASCTKLLLNAGADVRAAMGPGKATALHLAAEDGHAECARLLLDHGAHIDW

PNFRGQTPLHLATLAQSLEVVEMLVDRCADVRAKDADGRTPLHGAIVRGARACDIARLLLSAGADPNAAD

NFGYTPLHIAALNEFSACVLLLLDYGGDVTLRTNGGVSALSFIVRRVPDVIPRYLCKFDDAVHVSEHELG

DVDCELTIDFRPLVPCLTRGEAELLLAFVEIPYWDVDNGVLRTNTTNWQHDVAAITIFFCWLELMMIIGR

FPTFGLYVQMFTTVTVNFATFLLAYSCLLIAFGLAFSVLFSNYPAFHLPAGLVKTVMMMSGELEYEDIFY

NNCTNSEIYYPLTAHIMFLIFVLLVTVILTNLLVGLAVSDIQALQEGAGLDRLIPKEFISAIYKMVASRK

NTKRSVRKNNNYEFRIRRCPEEETISKPEIVKRKSNLYDRFSFDSQQVERRKRPTSGSNTDSRNRPPSLT

INAIQEICMVDIKTQLLELTKKVNKLLEGTEARLNQIESKVNGPLP

>BmTRPML

MHAPDDTTCSEGEDDHKKEIRPNANNEQDFPHQSTSFSPPTQMEEKMRRKLQFFFMNPIEKWKAKRKFPY

KFVVQVIKIVLVTFQLCLFAHNRYNHVNYTWDNRITFSHLFLLGWDSTREINAYPPGAGPLALYKIDEFY

NTLDYAVAGYTNLSNAIGPYSYNDENNFMPDPVFCQYNYKQGIIYGFNESYQFNSEVFETCINFTIKDNE

DFKSEAYIRDAGLNISFSSLVRAKLMFSIKTINFRAAGPITPPDCYRFDVEIIFDNEDHDGQMSLFLEAE

PHKLECKGDTAYVTDNKIDQILRSTLNILVILICAASFVLCSRALYRAQLLKELTVQFFRRQYDRDLSLD

GRLEFLNIWYIMIIINDLLIIMGSAIKEQIERNQFTNDQWNVCSLFLGTGNLLVWFGVLRYLGFFKTYNV

VILTLKKAAPKIFRFSICALLLYAGFMFCGWLILGPYHMKFRSLATTSECLFSLINGDDMFATFSIMSKK

SPMLWWFSRVYLYSFISLYIYVVLSLFISVIMDAYDTIKQYYKDGFPKSDLQQFIGETRIEEVSSGLYRV

HSSSSLNAIMNSLFCCNIYRSAYSKIGGRSSTVNLM

>BmTRPA1

STYIHLSTSTKVNLSFFVQAAESGNVDDFMRLYLSEPSRLAVRDGRGRTAAHQASARNNTNILHFINNYA

GDLNAKDNAGNTPLHVAVENEALDAIEYLLQQHVETSVLNEKCQAPIHMATELNKVSVLQVFAKYKSLFN

VNQGGEHGRTALHFAAIHDHDLCAKILITDLDAEWKKPCNNGYYPIHEAAKNASSRTIEVFLQWGEQRGC

TREQMISLYDNEGNVPLHSAVHGGDIKAVELCLRSGAKISTQQHDLSTPVHLACAQGALDIVKLMFTMQP

KEKHACLTSCDVQKMTPIHCAAMFDHPEIVNYLINEGSDINPLDKERRSPLLLAASRAGWRTVHTLIRLG

ADIQLKDINSRNVLHLVVMNGGRLEDFAASCKDHCEKSLLQLLNEKDNTGCSPLHYASREGHIRSLENLI

RLGACINLKNNNNESPLHFAARYGRFHTACQLLDSDKGTFIINESDGEGLTPLHIASREGHTRVVHLLLN

RGALLHRDHNGRNPLHLAAMSGYTKTIELLHSVHSHLLDQIDKDGNTSLHLATMENKPSSIALLLSMGCR

LSYNNMDMSAIDYAIYYKFPEAALAMVTHEQRAKEVMALRSDRHPCVTLALIAYMPRVFEAVQDKCITKA

NCKKDSKSFYIKYSFEALCPQLMDEDGTRKSQQAQQIPLPALNIKYSFKYYQKSKHEIEALRLAHNDPKY

RPEPLSVINAMVAHGRVELLAHPLSQKYLQMKWNSYGKYFHLVNVLFYCIFLIFVTVYSYLLMEHVNPIN

KRERSRVGDVYYNYTATNRTQTNWDIDADFEAILIMYTSSVVIMVYISVCMMREAYNLKQQKWHYIVDPS

NLVSWTLYISATITVFPTLYGHYSNYQFSAASITVFLSWFELLLLLQRFDQVGIYVVMFLEILQTLIKVL

MVFSILIIAFGLAFYILLSKVGSVEMGNHLSFSNIPIALMRTFAMMLGELDFVGTYVQPYYKDDSDIILP

FPIPTFIILALFMILMPILLMNLLIGLAVGDIESVRRNAQLKRLAMQVVLHTELERKLPAFLLEKVDKLE

LIEYPNNKKCKLGFLDLILHKWFCNPFTEDTGLDLVLENNDDYVTEQMEKQKRKFREMQNVLDQQYLLLR

LIVQKMEIKTEADDVDEGVSPNDTKVVPRWSSHRNRKKLHSARAASFNKST

>TcTRPA1

MDKKRYGVHNDLLQSLKDFQISLRRTTMPFVTLANVSINSGEALRLNIQAEGGRISIPEFPLGPRGLQRFRPPRQTLIEI

GTTKTARSWRIDTEDPQRLSPSTPIITVLPSVEVPEKTTIYPDEVKCETSQDRTRRDTLNWLMSSLRSILPSSGHKSASG

SPSELQNMLPSSVKVHRLSNAGKPPEDNGGICLMTESPFRILRVAECGNLETFQRLYFADPTRLSIKDSRGRTAAHQAAA

KNRITILQFILSQGGDLNNQDNAGNTPLHVAVEHESLDAVDFLLQAGVKTNILNDKKQAAIHLVTELNKVSVLEVMGKHK

DKIDILQGGEHGRTALHIAAIYDHEECARILISVFDACPRRPCNNGYYPIHEAAKNASSKTLEIFLQWGESRGCTREEMI

SFYDSEGNVPLHSAVHGGDIKAVELCLRSGAKISTQQHDLSTPVHLACAQGATDIVKLMFKMQPEEKLPCLASCDVQKMT

PLHCAAMFDHPEIVEFLINEGADINPMDKEKRSPLLLAALRGGWRTVHVLIRLGADINVKDVNRRNVLHLVVMNGGRLEQ

FASEVSKAKSQTSLLQLLNEKDINGCSPLHYASREGHIRSLENLIRLGATINLKNNNNESPLHFAARYGRYNTVRQLLDS

EKGTFIINESDGEGLTPLHIASKQGHTRVVQLLLNRGALLHRDHNGRNPLHLAAMNGYTQTIELLLSVHSHLLDQTDKDG

NTALHLATMENKPNAIALLLSMNCKLLYNQMEMSAIDYAIYYKFPEAALAMVTHEDRAEEVMALKSSKHPYVTLALIASM

PRVFEAVQDKCITKANCKKDSKSFYIKYNFSALQCSQFYADMDHKTGDALAISKPIPLPALNAMVSHGRVELLAHPLSQK

YLQMKWNSYGKYFHLTNVLFYSIFLTFVTCFAYEIMRHEDQIITYNATNLTHDEYVNFSKANILNVKITPMMYMSALAII

TYIILNTIREMVQVYQQKFMYFLDPNNLVTWVLYTCAVVMVFPIFWGTMYELQFSCASVTVFLSWFNLLLLLQRFDQVGI

YVVMFLEILQTLIKVLLVFSILIIAFGLAFYILLSRGDHLSFKTIPMSLVRTFSMMLGEIDFLGTYVKPYYLTTEDEKSF

LPFPLPAFFILGLFMVLMPILLMNLLIGLAVGDIESVRRNAQLKRLAMQVVLHTELERKLPKMLLERVDKCELIEYPNDT

KCKLGFFDSILRKWFGNPFSDEGLDMAMEGVEDYVVNELDKTKRKLKEITTALETQQQFLRLIVQKMEIKTEADDVDEGV

SPNDLKPITGHASKWTSPKIRKKLRSVVSFNNKGSST

>TcNan

MGNTESNVTSGVKKQAGASVQPIYKLCDLKGGGLLVELMKRATQNKQYAELDHAIKTKVEHFLYNKGAGRYFTISDLVLL

RNKERSRQKWLPQLKAMENPEDFEIDDEGPEITEEQYQKNPHLYRHVCWKIKERGAVGESIMHLCLLNATSLHADIAKRL

LRFYPKLINDIYMSDEYYGENVLHIAIVNEDPSMVKFLLDSGVNIQERCCGNFMCPEDQKSSRYDSLDHEWVNVCPVTNY

EGYVYWGEYPLTFAACLGQEESFRLMLSRGADPDAQDTNGNTVLHLLVILQKLEAFDMAYEVGAKLSIRNVLSLTPLTLA

AKLARIDMFFHILNLEREIYWQIVLGSITCAAYPLSQIDTIDIETGQISKTSALNLVVFGDKDEHLELMDGVLIDLLNAK

WNTFVKFKFYKQFFTFAFYFLISLVAFTLRPGPPHKEAKLVNVTINATVSNTSKWENLTSIPFGKTPDDDDSDMEEWWDN

LQEECRLMQLESPESKIRLTAEVLMVVGAFAYLAAAVREARFLGGRMFFENLMTAPSRVMFLFSCILMLTVPCLRLACLD

EFEDIVAVVIMLTTAPYFLFFCRGFKTVGPFVVMIYRMVMGDLIRFASIYLVFVMGFSQAFYIIFLSFDNPLTPDDVDDS

ATNPISTPIESIMAMFLMSMTNFGDYYAAFEKTDHEYEAKMLFVVFMVIVSILLINMLIAMMGNTYQKIAETRNEWQRQW

ARIVLVVERGVSPSERLKQLMVYSQPMSDGRRALVLRLNQSDEDKEEMKEILEMRRRHERHIKKRSEKLKENAKKN

>TcIav

MGAKVCKPCKKRKANTFQGGSILDRVISQASNQDQCLLYKLANYKKGGELIDAYNQGGQAEVEKLIREQFGQLMYQEGKG

QIINRSEYLRWKFRDHEQVILPIEASLSRYDPLAKWNDHEACWQMQFRGSLGESLLHVLIICDTKIHTRLARTLIKCFPK

LALDVVEGEEYLGASALHLAIAYNNNELVQDLVEAGANVNQRAIGSFFLPRDQQRQKPAKHTDYEGLAYLGEYPLAWAAC

CANESVYNLLLDSGAHPDYQDNFGNMILHMVVVCDKLDMFGYALRHPKLPASNGIVNKAGSPVMLELSAKEFWRYSNITCSAYFLNALDTLLPDGRTNWNSALFIILNGTKEEHLAMLDGGIIQRLLEEKWKTFARNQFLKRLLILVVHLLFLSLAVYLRPDDPDESLLTWSDDVTLIARYVCEVGTILGVLSYLVLQQGDEIRNQGLTAFLKQQLNSPPKLIFLISNFLILACIPCRLYGDKETEEAILCFAVPGSWFLLMFFAGAVRLTGPFVTMIYSMITGDMLTFGIIYTVFLFGFSQSFYFLYKGFPGVKTSLYNTYMSTWMALFQITLGNYEYSELSATTYPAVSKTVFAIFMVFVPILLLNMLIAMMGNTYAHVIEQSEKEWVKQWAKIVIALERAIPQSDAQHYLQEYSISLGPSEQDPSTEKRGVLVIKSKSKTRAKQRKGAVANWKRVGKVTINALKKRGLTGEEMRCLMWGRESINTPVKTKKPVKDPLLDPQGPNLTGGFGDALTTALDVMTFTHDLDIVGASQGLNLATNPKPVPPTTTASAVTVNNQINSALNAQQKAVAGAGVGALAMIGTVTQLTDSQGYIMQNSVKKEEKIVATLEDPFRELVINANDSNCDPEKLKMLALSAANLKDVEELSVAKPQTKSVKSLAGIFAGTETFVRKVEETIKKKYAALDPSDSEGFGEPPLLGKISRTRRAKSANLRNSSARSKASDKKKLVAGSQSSSTDTVNNEINEKNIENSDLDYAEERIKLVKESLKQVVDVAQIRPINIDVALEEQVSVTISETMRVQGSGDGAEVQSSNVKPKRKKRSKTAKNNK

>TcPkd2

MADRKPKTDSPEAVIRPNPDIKKGENKEQEKTEGKDEKPDKAKGKGEPKTETKETRVRWLTRAMAEDFTRHDVLFTTFRE

AVLYIIFVIAVTICTVGRRSASMFYITQALKGQFLEKDFQTANEKDIKYYDIRSATDFWHYTQSLMLENFYWEHYYNPYN

PVAKAKDDDKKILFENKLLGVPRIRQVKVKNDSCIIHEYFRRLFTSCYDLYGPTDEDRAPFGLEAGTAWTYNTAEKTESI

PFSGKISKYGGGGFYLDLSTNNEDTAKLIRDLKENLWITRGTRAIFIDFSIYNANLNLFCVCKLIFEFPPTGGIIPSHSF

HAVHLVHYVNSWDYVTFVFECTVYAIAGFFLAEEIREIIYFKLRYFLQFWTYIDVVIIGMAFANLITSIVVFPNVDDAIL

KIQKNPQKYGNLEYLAEMRIFYNNFAASLLFFSYIKLFKYLNFNKTMGQLNNTLKRCAFDILGFSIMFFIIFFAFALLGY

LLFGSQVEDFSSFGVAMFTLLRTILGDFDYQAIEKANRVLAPIYFLAYIFFVFFVLLNMFLAIINDTYADVKTEIAIAPD

EMQMTEFLKKGFYKMLQKCGCNIKYFQQQKAEFNATIQQIRDALKKCGFSDLEIEMFFARYNIDPLAEVGDYDIKKIMKE

LEGQSMAKEKVDEDTTLVHVSDFITQQERLDQIEKTIQMLATKIDTLIKKLEALENVRKAKATQG

>TcNompC

MSQPAGKKGGGKGPPKDKNDDSKDQNNKAAKDSKEKDKEETASNPEGDDTPSSKPQSAGANVRDAAQRILVLCQKGEWAP

VDQVLKSLEKSIAAAGDDANTVPLAGVADLATGMTPLMYAVKDNRTSLLDKLIDLGSDVGARNNDNYNVLHISAMYSRED

VVKLLLTKRGVDPYSTGGSRNQTAVHLVASRQTGTATAILRALLQAAGKDIRLKPDGRGKIPLLLAVEAGNQSMCRELLS

AQTAEQLKAAAANGDTALHLAVRRKDIDMVRILVDYGTSVDIRNGEGQTPLHIAAAEGDEALVKYFYGVRASASVTDNQD

RTPMHLAAENGHANIIELLADKFKASIFERTKDGSTLMHIASLNGHADCAAMLFKKGVYLHMPNKDGARSIHTAARYGHV

GIINTLLQKGEKVDVTTNENYTALHIAVESAKPAVVETLLGYGADVHVRGGKLKETALHIAARVKDGDRCALMLLKSGAG

PNLTTHDGQTPVHVAAQYGNLQTLLLLLEDGGDPQFKNKAGETALHLASRGCRPDVVQHLINYLKEHKGDEVAASYINEI

SEHDESALHYVSAVKKEDVEVPLADKEVVKLLLQNGADVKLQTKQHETAFHYVAKAGNNDVLMEMIAHMTPNDVQKALNK

QNLTGWTPLLIASHKGHQEMVNNLLSNHARVDVFDNEGRSALHLAAEHGYLQVCDFLLSNKAFINSKSRNGRTALHLAAM

NGYIHLVKFLIKDHNAVIDILTLKKQTPLHLAAAAGQIEVCRLLLELGADIDATDEQGQKPIHAACQNNFSEVAKLFLQQ

HPSLVMATTKDGNTCAHIAAAQGSVTVIEELMKFDRQGVISARNKLTDATPLQIAAEGGHAEVVKALVRAGASVTDENKG

GFTAVHLAAQNGHGQVLEVLRSSNTLRVTSKKLGVTPLHVAAYFGQADTVRELLTHVPGTVKSEPPNGASLVPALGNESG

MTPLHLASFSGNENVVRLLLNSAGVQVDAATHENGYNPMHLACYGGHVTVVGLLLSRSAELLQSHDKHGKTGLHIAATHG

HYQMVEVLLGQGAEINAPDKNGWTPLHCASRAGCFEVVKLLTESGASPKSETNLGAVPIWFAASEGHHDVLEYLMTKEHD

TYALMEDRRFVYNLMVCSKNHNNKPIEEFILVSPAPVDTAAKLSNILIVLSGKEKERAKDLISAGKYCEAMATELLALAA

GADSAGKILTATDRRNVEFLDVLIENEQKEVIAHTVVQRYLQELWRGALNWAAWRTLLLFVLFIICPPVWIAFTLPLGHK

YYKVPIIKFMSYLTSHIYLMLFLLIVGITPPYPVVRKNLLPYSYEWILLIWLSGLLLFELTNPSDKSGLGWIKLSVLLFS

IFGVGVHLMGLLFIDRNYWPTLMYCRNQLFALSFVLACVQILDFLSFHHLFGPWAIIIGNLMKDLARFLAVLAIFVFGFS

MQFVALNQPFKNGELNPRRGKYFVDEQEALAEWIEPLPEESPTWRASLRKKRPPPMVMNPLLAFELLFFAVFGMTTYDEL

TAKDRSDPKRLLRPAWTDNLFKVVFGIYMLVSVVVLINLLIAMMSDTYQRIQVIIRFQKNPIRAFELLFFAVFGQTNTDA

LKIDTYNTATKNNNQPTWTEVLFKIVFGVYMLVSVVVLINLLIAMMTDTYQRIQAQSDIEWKFGLSKLIRSMHRTTTAPS

PINLITTWLFYLVNICKKRVNESVKARKRQSLVHLMGSFQRPQQLSPRSKAGAKWLSKVKKGSQVAPKESVALSVAHLSP

LGSQLSFTAHTTRIEHVTDWEAIAKKYRALMGEVDEVKEQNADEENESDEDAVQASNSVQPVT

>TcTRPM

mnepcgcgrt laqhkhntet vpplageiwf psrcttalpt daygilefqg gphpskaqyi

rvahdtkpeh lmqlitkewn lelpkllisi qggkanfelq pklkkvlrkg llkaarstga

wiftggtntg vtkhigdall lersqrtgrv ntlgiapwgi vennqeligh ntevpyhsis

sprskfaaln nrhayfllvd ngtvgkygae ivlrrrleky iskqrlypft qspipivclv

ieggtntira vleyvtddpp vpivvcdgsg raadliafmc kyelsvlksm rdyiiatitr

afevnrelae clygelmqcv enknlitvfr iadkydqkpq eldqtiltal fksqhlspte

qlslaltwnr adiarseifi ygqewprgal eeammkaleh drcdfvklll engvsmrkfl

tiprlenlyn skegpsnilr yilrdvrphi pagyvytlhd iglvinklmg gayrafytrr

kfrpiyakvm nkgqnmqrns tsfvkqygna msllaqalps nanpclfdyp fnelliwavl

mkrhkmallm wqhgeealak alvgcklyka maheaaeddm etevyeelrs ygkefenial

evldfcyrqd ddqtqqlltc elqnwsgqtc lslavaanhr allahpcsqi iladlwmggl

rtrkntnlki mlgllcppyi lklefkskee lqlmpqttee hmelendddd ksdsdknvdg

evsvmlsdpe enkyhvfftd vpqervprnr elkiskklye fytapitkfw ansiayitfl

iifsytilvk mderpswqew laisfmctyg ceklrelfss epvglkqkla vwcwnlwnpc

dmaavlffli gvvlrfkest fevgrvfycv nsiywylhil nilsvnkylg plvtmmgkmv

knmiyfvvll livlmsfgvs rqailfpdtp pswgiardvf lepyfmlyge vyadkidppc

gedeekpcqt grwvspvims vyllvanill inlliavfnn ifnevnavah qvwmfqrftv

vmeyeqkpil pppfiifchi yllikylrrk megkeesydn glklfldrde merlydfeed

cvegyfaeqe lklqqsteer ikvtterieh ltqkiedint kenihttalq nlelrcrrlv

dqmqevstem ekirtsiesq gstagpsfda gfirertvse ptealvedvp tlkigssatk

rkpivrslte vrpdayifdn gqhieyryde eeecneeidp lpksesqqpl tferqrtrst

dsktsndsae vsaddlgals levlrnwaiq krkdstgtgr rssdggnged ssngskrsln

krqlsqthse petsdppata tnpshpmtle rsvtftepri kvipptsiag gsnrtallmh

mhteytsitd elesvcglls pprspgllsp prpeqsppsq rkrhpsemsn peiainfeke

hlrsaeecdy mvmenliqrr ydeedgehgp lnpdllsvvt etrefrissi gsrplkrasa

vegdaqplgi tvsckavavt nfadsgsdsa iqgdsterqd stesnditsa llnqppmpqr

psivmdssql piqrevqkae skdslhmqse tmc

>TcTRPML

MEPMQGNSPESVTTCGTDDERCCLTHQPSCHSSKSLLTEEKMRRKLQFFFMNPIEKWQAKKRFPYKFTVQLIKIILVTMQ

LCLFAYNRYNHVNYTWDNRISFSHLFLRGWDATREVSAYPPATGPLAIYKADDFYATIDYAYSGYANLSQAVGSYSYANE

DNTMTDMELCLSQYKKGIIFGFNESYVFDAEIVDTCLNITHRQDHLLDSKQYLDNLNINFSALVKAVLKFSVKTVNFKTA

GRISPPNCYRFDININFDNEDHDGQMLLWLDAQPVRLMCKGDVEYVTDDEIDSLLQSLLNYLVITICILSFVLCTRALLR

AQKLKKMTNSFFINQFGRPLSKEDRNKFLNLWYVMIIINDILIIIGSSIKEQIERKDFTSDQWNVCSLFLGLGNMLVWFG

VLRYLTFFKTYNVVILTLEKAAPQVARFLLCALLIYAGFTFSGWLILGPYHLKFRSLSTTSECLFSLINGDDMFATFSIM

SSKSTMLWWFSKIYLYSFISLYIYIILSLFISVIMDAYDTIKIYYIEGFPKSDLQIFIGDTNFEDVSSGIFRTASNDSLG

GLMKDLCCCTCTKLQKSYSSLSRGSLATEGSRRGSNAV

>TcXP_968670

mfgsdcnner alsqveksfl lhaergdcat vrriieeygg gegdqefdin cidalnrsaf

viaiengnie lvklllesqi dvkdgllhai neefveavem lldweerthq vgdlyswekv

ercsatftpd itplvlaghk nnyeiiklll drgatlplph dvkcgcdecv csnqsdslrh

sqarinsyka ltspslisls stdplftafe lsgdlrrlsr letefraeyn nmrtvvenfa

allldhvkts eelmillnyd eknpswvlge qqtlgrlkla ikfkqkkfva hpnvqqilgt

iwyeglpgfr rknlfgqtlq lmrlsamftv yclvymllpt skmglfikkp fvkfichsss

yilflvllgv tsqrfeitil ewvdtgwsrd fvdewkrker gallgfaeca vvlyllklvf

lgkmckeikh lwasnlleyv sdlwnivdfv tnlfyvawls lrlcsiyltr asvwslgedp

wfpreewnsf epmliaegaf aaamifsflk lvhifsvnph lgplqislsr miidivkfff

iytlvlfafg cglnqllwyy aeleknkcyh rggmpdfeah dkactiwrry anlfetsqsl

fwasfglvdl lsfeltgiks ftrfwallmf gsysviniiv llnmliamms nsfqiisera

dvewkfarsk lwmtyfeddd vlpppfnilp ntelfcrvfk lrrkknkicn kesqlrikak

hdsvvrlllk ryntseqrkr hefgvteddv ieirqdistl ryeiidilkn ngmrtpnmsf

edkqvvgkkg kmierrilrd fhigvvdevg ksevsyspnk rkrrtfrkek kdwnslvrkn

tksydpigss knhfe

>TcXP_968598

mgdsvdveak vtrktlimpn lpkslsieek kyllaiergd manvrrmlqk ahrkksfdin

cvdslgrgal siaidqehle mvellvimgv etrdallhai hvefveavel lleheelihk

dgepyswqkv dintamftpd itpltlaahr nnyeilkill drgatipmph dikcgcdeci

keseedylrh slarlneyka laspslials ssdpiltafq lswelrnlaf aepeckseym

dlrrqcqqfa vdllhqtrss qelafilnhd paappyeege hmklarlela iqykqkkfva

hpniqqllaa iwyegvpgfr rksstqkimi ivkvavlfpf ycmlymitpg tktgklmrkp

fmkflihass ylfflfllil vsqraedqii elfgtesmkq nlqekyrkqr gnpptileyv

vliyvigfif eetheiyveg mksylrnlwn fidftrnfly tavfllrvaa yiqqtseien

dpftayirre ewqafdpqli aeglfaaani fsalklvhlf sinphlgplq islgrmvidi

vkfffiytlv lfafacglnq llwyfsdlek kkcyhlpsge adfdnaadsc mkwrrfanvf

essqslfwas fgmvdlasfe ltgiktytrf wgllmfgsys vinvivllnl liammsnsya

midehsdtew kfartklwms yfeesatlps pfnifpkpkh lfkllglrkk dkirkmstkr

rnreekerdy kytavmralv wryvsamhrk leenavtedd vnelksdiss fryellevlq

kngmdissae mkekavlgrk mkvwerrlmk dfkvapvvte eeeefiyqpp pegenslakf

rriarlavvn snlskwrqvv kgaciasqig hchsrdsfkk qqnlqramee arklkvkspd

qsrattpiql pdttgsniik iirgitpetv dtkycltprd ksplisispp ktpekepdli

nlesaktspv lpvkampsdd vslesvdkii ndsnsldddk vrslptspts sddksldsls

kkalfssgsf tpeenekkee etepknvtfs fdfidesskg essaeilkks eeeteenldk

sdepepslee tkeeseevhd lpeetvqaks edeveetenn eieepqadle esepqldeke

eesqvkseel tveekeiqve vekvcaksvd eleapekkee veekiekeks plavrniqri

gdrgrrqpkt gwl

>TcXP_970049

msrdpenpda tipppppppm pnfgrgpgrm gyrrleedde llmprpsill phlqesekrf

felvhsgdva avndflnenp gfnincvnfq gvsglliavq srseamvefl lsqpdidigd

cvlhairdnq pkilelllek qrntapsley vgvthssdfp dyvtplilaa qcghyeiiem

lidrghtisk phspscrsvp kvryghccrc mdckaqlerd dllhaehlrl nlyksvcnpa

yichssrdpi ltsfqlstel rqcsflvpef rnsylelane vsnfavelia gcrnsgevet

ilkqkagiqn asifmyprlv lamdykqkff vahahvqhiv eslwrgnwyd ynlkpipaki

iypifrilll piivvmcifl pkhqmvahwn iplnrminhv aaflvflvii flesnrsken

qkrmppnsgl epviiifvva ncwsvvrmcl iqgprryfty lwnwhavisn tffvltfvfw

lasyvdavnn dqvdlerkyw hhldpvliae gtfavavimt yfklmffcrl nyylgplqis

lgkmcsdmak yitifiiiii sftaglcrfy syydgmvqvd sngiktaqvs sfidfsttlk

tffwavfcma pletgdviie nlpgdtentt iinkhlftea vgyiafalfe vltvvmilnm

liatmsstfq rvldnidvew tfgktdfyle ymlqptlppp lnliptpsgi safmewfqsp

kvdpreeekr ndypalmsql vqryfrdkds aaspendlef lkqeiaeikq avndlleke

>TcPain

MGGRSKKMDLVRTNSICPPPETVLLESVKKNDTESMKTVIKANPSILQHLYPYTSQTILSLACSEPGVAPDTVNQLIELG

ADIEGTPQWKPLHLSADNPNILILEVVIKHLKPGQINEKWNGNTALHTLIKGEKIKKDEENFCRHVELLLQSGIDVNQGD

GKNLTAIFWAAKYGYKKIVKVILEESVLHVDLDTCSLRDKTARDLINEKGLYEGPLPERILYQVPKDKIFGLIKEGKEDE

FISYFETLSLNNPSDFVNADDGASTLLQYSCERGLAKVVEYLLDKGANVNLVTKNQRKPIDIVADIGYFEIFELLFKCPD

LELSIDTLCNLLKHSNSPKFGKINHEKCCKLLLDKLGNRRPPIDINEVDHLNNIPLHYSLRYCDTTTTQKLLQLGASLAY

KNEFGSMPIQDIKPEVLENHLDNCVTFDLKNAEKRDFEVVFDYQTLLPPRKRKRFKYEEVDPEFLATNNVKPETEVIAYM

SKAPEFRALLKHPLIVSFLFMKWHQIRLLFYTNLVFYICFVLSLVVYIFTHYANFDRTQSDYCLIFGKFSWFTLNLTFWV

LVLREVFQVAVAPRKYFCNFENLVEIILIVVTGMILYIDSPTSHTRRQLASVAILLAAFELVLMVGQHPKLSTNVVMLKT

VSVNFFKLLLWYSLLIIAFALSFYILFAKTEMAQSVNGTETGDEDDVFKGPGKSLFKTIVMLTGEFDAGSINFHTYPVTS

KIIFSLFVFMITIILLNLLNGLAVSDTQTIKNDAELVGHISRAQHIYYVESMLLGNILPTSFIQAVQRLFCCCPCDSDTT

YTFFKPLSRKVCLFTQNCQLTVLPNEYGKISCELNSSAKKRPDPLVTCTRNCSEAYLDKNTIRRIADIVKARREREEYPT

VTNLKQLYGEIVSIKAKLDQILGSLSTNQNAL

>TcTRPA5

MSSSISPLHRAVTSGDVSLVKRLLAAGSDPNAADSHGRTPIVFAECIKNDELFEQIVELLIAHGADVNACNDTPLYSAVF

YGKKHLVERLLKAGAGVRSNNLLHIVAEKGDDTILALLLGDKRSEEMIDGEDANGRTSAFLAAQHDHKRCLKMLIAKGAD

LSRADLQGESVMEAIFENMTKPVEFLTEVLDASVTLERGEKNKYFVASEEEKTSVLQHPVIELYLSIKWSRMCYFFYLWI

FAYVLFVLSFSVYVMLCRDYRNLDVLVTASRWISIWSGACLLGHGVLECCLTHGNHFRKYEMWLNLACTSLSLIVAIAGK

RDGNREDELAAPNWVLHVTSIAILLSWTELMLLIGRLPTYGYYALMFSAVLQNVIRVLLAFLCLVVGFSLSFSIEFANCS

EFNDPWRALVKTTVMMMGEFDYSDLFTGLERPASRVIFLLFVILTSIVLMNLMVGLAVSDIQCLQMVSHARKLEKMADFL

AQLEKVLTSEKFKKRWLPQIVQKILRRNFIDVRYELETSARFRRSKKLSSKLIGSWCNVIIEHRLRRRTCSRLPYILIPS

SSPRGPSRIINPNRGPSPKQ

>TcWtrw

MDNMGFQDSTNSRKLGRSVSVNAEVESFPLYKTHTCPSGLRWRRPRDDPEIVVEEEFPSDDFEYMECGPSPPADHTPNIY

ESFEEFSRSELTVQICHDTIKMNLLEHMKMASGRHQLLDDIECKKTTQDTLTEAFQGATKLEINIAFLWAAFMKRWELLD

GLLKLGAQLRYYEPSQGLSALHLASFSGCIPGTQFLLAQGCDVNAIFKCYTPLHCSAFGDSPETAMILLNNGAKVQALTN

SPNNCHESVLHCAVRANAIACVRLFTAEGADVGQFEFSGMSPVHLAADLGHPQCLKIMLEAKGVNVNAKTKEKELTPLHL

AAEGGYVECVEILLDKGADANIRNHRGQTPLHLAARAQAYDCVEMLLRKGNADPNIGDFDKRTPLHAAVCKAARSYDIIE

ILVSWGADVNTKDQYGYTPLHIAALNELSQCVEILIYHGADVTAKSKFGMTALGIITRKTPASLAMVTHKLDSAITLHHH

PESSNREVELRLDFRSILQHCHPREISFLNTFVDEGQKEILLHPLCSAFLYLKWEKIRKYYIARMLFCFIFVLSLSLYVL

TALAHNCYNHGKNMNDTQPQNVIELCEKKSMMGHMLRTNPFVIEMQWFVLVGITCCEILRKVYGIAGYPTVKQYLSHPEN

IIEWTVVVSVFVISFIYTGRTYTWQNHIGAFAVLFGWTNLMLMIGQLPVFGSYVAMYTRVQGEFAKLLLAYSCLLIGFTI

SFCVIFPDSSTFANPFIGLITVLVMMTGELNLDLLVDDDPEDPPFLLEISAQVTYILFLLFVTVILMNLLVGIAVHDIQG

LQKTAGLSKLVRQTKLISYMELALFNGYLPRYLLNLLHWTALVSPKAYRVVLNVKPLNPREKRLPRDILKAAHEIAKKRK

HYAHTISSHGSNATTRKIINNNMDQNSEILEYPSAFPLLQSKIEKSTEQIEHLSKEVRELKIALQNNQKIMEQLLHGLVN

TKNSSNC

>TcPyr

MSLRHERPGKTIEESTVRWNGDISLDMTDEMGSISSEDETGSEYDGEQRSRGKSVLEIWDDDYIQARLQASILPDAEDII

EMIEQDNTETIFTKNPTSLLLIATWLQKEKVLQEVLEKGVSLQAVDGEGRSALHLAACTGNIDCIKLLLQHGAEISARDA

LNRATPLHCAASKGHLSAVKLLIRHGADVNAGLDNKSPLHYAVQSLAIDCVKELLENNAIPNTSQVYSETPLHVAAALGA

PEIVKLLLDHGAAVNVQCGTDKLTPLHLAAEDSDAESARLLIDAGAQLTSENHKKQTPLHLAALSQCSETLELLLARGCN

PNARDADGRTPLHGAIVKVSRSCECVRLLLKAGADVNRQDSFGYTPLHLAALNEFSNCVMMLLNHGGDVTVRTNGGVSVL

SFITRKTPDVIPRYISKFDSSIKINDHEIGDVDCELKLDFRILVPTMGHQETELLLNFIEVGHREVLKHPLCETFLFLKW

RRIRKFFLFSLFYHSLFVLLFSIYTIGVYIQDCPSFRALLTRPCRVPQYYNIIGYILLVFNFMFLAKELFQICHSWRSYI

QQWENWLQWLIIISVFCCVQPSLDNDMDIRYKVMRWQHHVAAVGIFLAWVELMMIVGRFPIFGLYIQMFTTVAVNFIKFV

IAYFCLLLAFAFSFGVLFAKYKSFKLLKWIIIKVLVMMSGELEYEDIFYDEEAPIQYDYTSQFVFLAYVILVTIILANLL

VGLAVSDIQGLQQSAGLDRLVRQAELVAHLESMLFSRLLTCIPHKLMHFFHKKALLLKSQYHWALYIKPNDPREERIPKD

LIKNIYQLVAERKEKPRKKRRSNKIKSDFVSPPMSRLNSISDSYGGADKQVALKCELEEIQKEFAEFTRTFREKMEGITN

QIKNTKS

AmTRPM

maigsiicga stpkmkrkka katerswiea tfqkrecskf ipsaddehkf mqvkgdknae

ydvittsrcc cgysythhcr agidvqsytl sntkekdreq wspakntrpf ptdaygtief

qggphptkaq yvrlaydtrp epivhllcre wnlglpklli tvhggrsnfe lqptlkkvlr

kgllkaaktt gawiftggtn tgvtrqvgda lllersqrqg rvvsigiapw gildkshelv

grggevsydc lsspwskyav lnnrhayfll vdngtggryg aeivlrrrlekyisnlklqp

ythssipvva lvieggtnti rsvleyvtdv ppvpvvvcdg sgraadliaf mhkyasegdg

engdiegple smrehlldti krtfkvsaeq asqlysellq ctrkkhlitv frisqerpqe

ldqtiltalf kskqlspaeq lslsliwnrv diarceifvy gqnwppgale qammqalqhd

ridfvkllle ngvsmrkfls iprleelynt kegpsntlgy ilrdvrpnip rgymytlhdi

glvinklmgg ayrsqytrrr frmiytkvmk rsgahpqhlh rnscilgntt ryysgsgskq

dsltmsllae tlpanrdtpl fdypfnelli wavltkrqqm allmwqhgee alakalvalk

lykamaheaa eddletevyd elrsygkefe nigtnicdfc yrqdddqtkq lltselqnws

gqtclslavt anhrpllahp csqiiladlw mgglrtrknt nlkvvlglic pfyiiclefk

sreelqlmpq tqeehliale dekedsdseh giptgpdvea lisnehttti vketivqeng

kvltdnddgi hraygihsdy ydiknsrplr lrkklyefyt apitkfwana iaymiflvlf

sysilihmdd hpslaeiyai ayictlgcek vreiatsepa tlshkfsvwa wnmwnpcdaa

aiiffqigla lrlrhstldv grviycvdci ywylrilnil gvnkyfgplv tmmgkmvknm

tyfvvllivv llsfgvtrqa ilnpnaepkf riirdifmep yfmlygevya dnidpdcgde

pgmipclpgr witpavmsiy llianillin lliavfnnif nevnavahqv wmfqrftvvm

eyeqkpvlpp plivvchiyl vvkyllryit qgkassgety dnglklflea ddmerlydfe

edcvegyfre qelklqmste ervkitterv enmhskiedi dkkentqnas lqavefrirk

leelneqtla hlgvihrfma thmpniegls nfdiegrqrr vsersevlse tdshtqlpti

takrkrlvrs mtdatflnlg psldddimkh setimsrenl srnessisgd ghiiqddikt

ttsqetefsk idgeretikk dsqsdsrets repseepssk epstdpsrqt srdlsretsk

datskepsre asseapasep irqdsierpi rqnsrtrses ddimilpsni prgvtwaepr

vavipsvsst ntqrsillam raeytsitde lesycgllsp prtppisppp srvrnlsems

npemawqien ehlrdaeecd yqqmedliqr ryiaddeplh vsdeasggsf fisnehrhql

rrtsaidees rrppptisvt reieqtlsrp pirdsensdp ndknlstvpa pasetmc

>AmWtrw

MAVIFTRFVNIQNAINNNNFGSLKNTWNIVQYSGVIFKSYCVNNLKNNISFDNSKKGKKISKVMKIYLERSREYQNFIRK

ETLEYNIGKRNLAKMMGEDPDNFTKENIKNAISYLFPSGIYDKEARPMMADPEELYAREKEAEFNEDGRPHHFLYYTIKP

NYYEILHKMVESITYLNKIEDKFLLINHKIDEDKKIDLNGSDWLEKSKLENILLEKLSDSEYEYFIKSMQRLADHPVSKH

VEPFIMEYRKSLTKTNENIELPKPQYDNDNRPFVLIEKCQRKSSIGQVKVIGNGSGNITINGQDITYFSDMYCREQVRNV

DMENVTVSIPSTPKRTTMTSLLLTRWLTSKSSTRTNPETSWSSDDRSLEYGTPPPIEEPHCYSMCASESSCTEEINCTLQ

VNKDIIKNLLVEHMRHIGGRLQLLDDLEENKMDLKNTENFAKLYKQHEINALLLHSSFLGHVDAIKSLHKCGADLNHSEQ

GQGLTPLHLCAFSNCLEGVQYLLDNGANIHLERTHTPFHYAAFGNACKVAQYFLQLGISQESCYGEETVLHSAARSDAYN

VLKLLAPNNPTLDNLDCNGYAAIHHVADRGDPSCLTVLLDAGCKLDLTTKKNDTALHLAAAASCVENVDLLVERGANVHL

RNHRDQTALHIAARSHSLECVEILLKKGGCDPNIEDSDGRTPLHLALGRSLLAYDVTEFLITWKANVNKTDKYGYTPLHI

AALNELSQCVDILIQHGADLSARTKGGTSALSIILRKTPTSLNVFKQRLDASITLHQHGSATGEAELRLDFHPLLMNQQQ

GEIRYLGTFVKEGYKEILEHPLCQAFLHLKWQKIRKYYVGRLIFYLFYVLILTGWVMTALAHNCYNESHGQLDNGQPPLC

ANTTGINGFLYRHPALLEVEWYALMVLTILEVFRKLTSIPTYPCVRQFFTQAENMVEWCVILSVFATSFIYTGRTYPWQS

HVGAFAVLCGWSNLMLMIGQLPIFGAYVAMFTSVQAQVFKLLLAYACLLVGFTASFCVIFPRSKSFSSPHIGLIKVLVMM

TGELNFEDLFFPHEEDGKTIDSGSTSWILLQVSAQLSFVLFLLFVTIVLMNLLVGIAVHDIKGLQKTAGLAKLVRQTKLI

CDVETALFLGLMPKRLMKFLRWTALLLPSPLRAVLTVRPLNPRESRLPRDLLATAHKVAKERKNCISGTLYNRKRNAVYA

CLKSEPYLTFRRNFPEEEISTIDDYAIKGELAELKKICERNHQLLQDIVLMLVNDKRE

>AmIav

MGGVCSFRGRGSQVNAGSILDRVISQASDEDQCLLYRLANYKKGGELIESYNQGGQFEVEKLIREQFGVLMYADGKGQVI

NRAEYLRWKFRDLEQVVLPIEASLSQFDPLAQWNDHEACWQMQYRGSLGETLLHVLIICDTRIHTRVARILLKCFPRLAI

DVVEGEEYLGASALHLAIAYNNNELVQDLVEAGAIISQRAIGSFFLPRDQQRTNPAKNTDYEGLAYLGEYPLAWAACCAN

ESVYNLLLDSGADPDEQDSFGNMILHMVVVCDKLDMFGYALRHPKLPARNGIVNAAGLTPLTLACQLGRAEVFREMLELS

AREFWRYSNITCSAYPLNALDTLLPDGRTNWNSALFIILNGTKEEHLDMLDGGIIQRLLEEKWKTFARFLKRLIILAFHL

TSLSLAVYLRPSNTDAQLLKWPEEITEVARTIAECITVLGVLSYILVQLGGEIINIGLLSFMKQLSHEPAKLIFLISNLL

ILACIPCRLAGNRHAEDAILIVAVPGSWFLLMFFAGYVYVIVSWKVENTLGKLYKLLCLTDELVDALLMDHLFSKTCCIS

TQAVRLTGPFVTMVYSMITGDMLTFGIIYMVVLFGFCQSFYFLYKGFPGVKSSLYSSYHSTWMALFQITLGDYNYTDLSY

TTYPNLSKMVFAIFMVLVPILLLNMLIAMMGNTYAHVIEQSEKEWVKQWAKIVVSLERAVSQKDAQNYLQEYSIKLGPGD

DPNNPAAEQRGVLIIKSKSKTKAKQRKGAVANWKRVGKVTINELKKRGLTGEELRRIMWGRASFSTPVRVSPKGVEPQVS

VVTAGFGDALTTALDVMTFAHDLDLSTATEGIPTNIDAKQSKPKSATKETKSTVNNQQNVTTNIEPLKSTTENVDDQANN

PREKKVTSQAATVHEMNLKNANHSSVTEDFQDPLLELVIASENTNDPETLLEIAKRAAAGFETETSSKINLQILEQFTMT

KIPMDEKVNVTRKQYFVESSDNDFGGDNLLGTEARLRRIRSANNRFITTRRRSRNVDDDLSSTSSTSMDRNPRYQSLLNG

HENSIDRPIESRECSIEAINSQIKQNGPCETMKAKVQKKRPKTARNRCINLQHADLDDKDIETILHWHNTYRNTVASGKE

IRGNPGPQRPAKFMMEVMWDDELALIARRWVVQCNLLEKDQCRDVGK

>AmNan

MGNTESNVASGVKKQTDASSILLYKLVDLKGGGLLVDMMKRATQTKQYAELDHALRTKVEPYLYNKGKGKWIPIEKLVLL

RNKDRPKHKMLPPLRAMENPADYDIDKDMGEDEVDETKIDKSKYRLVCWSLSERGAVGETILHLCMLHATAIHIDLAKRL

LRFYPKLINDVYISDEYYGESALHIAIVNEDPSMVKFLLDSGADVHERCIGNFMCPEDQKASRADSLDHEWVCVTPETNY

NGYVYWGEYPLNFAACLGQEECYRLILARGADPDKQDTNGNTVLHMLVIYEKLATFDMAYEVGASLAIRNAQHLTPLTLS

AKLAKIEMFFHILNIEREIYWQIGSITCAAYPLSQVDTIDVDTGSISHNSALNLVVFGEKDEHLELMDGILVDLLNAKWN

TFVKFRFYRQFFLFCFYFVLSLISFTLRPGPATTSNDAKPIDETVLRINSTILFNGDGISFTSKYNWWNNLTEECRLMHL

DTLSTKIRLTAEVLMEIAATLYIFAALREARFLGLNMFIENLMTAPSRVMFLFSCCILLTFPFLRLICADEIEDMLAVVV

MLTTAPYFLFFCRGFKTVGPFVVMIYRMIMGDLLRFVSIYLVFVMGFSQAYYIIFLSFDNPNTPEGVDDSMSNPMPSPIE

SIMAMFLMSMTNFGDYYGAFERTQHEMEAKFLFVVYMAIVAILLVNMLIAMMGNTYQKIAETRNEWQRQWARIVLVVERG

VSPDERLKKLMDYSQPMSDGRRALVLRLNQSEEDKEEMKEILEMKRTHDKLYKRRQSKTMKKILTSEDNVIL

>AmNompC

MSSSGSKRASGGAKDERKNASSKEESPVTGRDRDEGSGGTSADGTPQPGSKPGSAGASSREAVQKLLALAARGEWAPVDQ

LLKSLEKAAQSAGEDGGPLLPLASVMDPATGMTPLMYAVKDNRTGLLDRMIELGADVGARNNDNYNALHVAAMYSREDVV

KLLLSKRSVDPYATGGPRQQTAIHLVASRQTGTATSILRALLAAAGRDIRLKVDGKGKIPLLLAVEAGNQSMCRELLAQQ

APDQLRATTATGDSALHLAARRRDIDMVRILVDYGATVDMQNGDGQTALHIASAEGDETLVKYFYGVRASASITDHQDRT

PMHLAAENGHASIIELLADKFKASIFERTKDGSTLMHIASLNGHSECATMLFKKGVYLHMPNKRGARSIHTAAKYGHVGI

ISTLLQRGEKVDATTNDNYTALHIAVENAKPAVVETLLGYGAEVHVRGGKLRETPLHIAARVADGDRCALMLLKSGAGPN

LTTDDGQTPVHVAASHGNLATLLLLLEDGGDPMCKSKNGETPLHLACRGCKADVVRHLIKFVKERRGAETATSYVNSLTN

EGASGLHYAAQIEPSEVGTAGDDRAVIRALLEGGADVSLQTKQAQESAFHHCALAGNNEILSEMISGMSATEVQKALNRQ

SAVGWTPLLIAAHRGHMELVTTLLANHARVDVFDLEGRSALHLAAEHGYLQVCDALLANKAFINSKSRVGRTALHLAAMN

GYSHLVKFLVQDHGAAIDVLTLRKQTPLHLAAGAGQLEVCKLLLELGASIDATDDQGQKPIHAAAMNNYAEVAQLFLQRH

PSLVMACTKDGNTCAHIAAMQGSVRVIEELMKFDRQGVISARNKLTEATPLQLAAEGGHAEVVRALVRAGASCADENRAG

FTAVHLAAQHGHGQVLEVMRSSQSLRISSKKLGVTALHVAAYFGQADTVRELLTHVPGTVKSDPPTGGSLVGELGSESGM

TPLHLAAYSGNENVVRLLLNSAGVQVEAATTENGFNPLHLACFGGHITVVGLLLSRSAELLHSSDRYGKTGLHIAATHGH

YQMVEVLLGQGAEINATDKNGWTPLHCAARAGYLDVVKLLVESGASPKSETNLGSAPIWFAASEGHNDVLKYLMEKEHDT

YALMEDKRFVYNMMVCSKNNNNKPIEEFVLVSPAPVDTAAKLSNIYMKLSEKEKERAKDLIAAGKQCEAMATELLALAAG

ADSAGRILTSMDRRNVEFLDVLIENEQKEVIAHTVVQRYLQELWQGSLNWNAFRTILLFVAFLVCPPVWVVFALPLGHKY

NNVPIIKFMSYLTSHIYLMVFLLLVGIIPIYPVVRASLLPYWYEWCLLVMLSGLLLFELTNPSDKSGLGWIKLAVLLFGI

CGVAFHLMGFVIVHRPYWPTLLYLRNQLFALSFLLACVQILDFLSFHHLFGPWAIIIGNLMKDLARFLAVLAIFVFGFSM

HFVALNQAFETQPDGGRRMDDKKKKGVFNDDLLTNVTEWMMETPSTAPPRRYGRYKKKECCDDNSRDIKMNPVLAFEYLF

FAVFGQTTHGELKVETNQPQWTSVLFKLAFGVYMLVSVVVLINLLIAMMSDTYQRIQAQSDIEWKYGLSKLIRNMHRTTT

APSPLNLLTTWIVYFIKVCKQHAAKRKRPSLVHMMGLQRAARLSPRSKMGAKWLAKVKKGQVRPKDSVTLSVVHLSPLGS

QLSFNSATRIENVVDWDSIRKKYLALAGNEPEKEADKDAKNEDEENEDENIHMVANSSTIPTTATPPV

>AmPain

MDLEDETLQMHLLHDYTTNSIKSQTIYKLLLDYLRTKNFRHFKCLVEQNLKKQPPIININYAYPNQSNETFLDIACKNGL

SEFVKFLLEKGAKVNRINEVHNRGPIHFATENGHADVLSILLDEPTINPNLEVVQQTALHIAVKKNDLKCASLLLEKGAS

PNIPNNKGLTALHIAAMKDYRNMVNLILEKTKHALNLDTYKDYNDQTARQILEKKIPNISLPPIEKQNVNIHDLKYYLNA

NDEMNFLRCLKIVQNDMLNNDIETLIEMAVQKNFKEAIILLLERTKEIKCNLEKAANLAIQRGSPHILRQILETDIEVKS

DLLLNACIELNIPHKGGSQDMSDRLECFNLILEREDVDVRCIDGKGNTPLHYAAKADCREAVTLLLEKGSYIGHMNNFGI

PPVADISISTLSQYFDDCIVARKERTNEYTIEFDYKCLMPHDNSYIINQQKNFKNQEKREMDIFQYIASNNSLKHLLKHP

LLSSFLYLKWHRIRYILYLNFAFYILFYLLLNIYILQITYVKNTQISTNSSEQMNGEIKNSISIYILQIFTGIMTALFAF

REILQLLSSPCHYMLCLENWIEMTLIILGFSILNGATTQVAAVTILLSAWELVILIGKHPRMSTGIEMFKTVSFNFMRFL

FLYAFLILAFALAFFILFKDGGNENFPDPGHSLFKTIIMLTGEFDANDIPFVSHPILSHFVFILFVFLIAIVLFNLLNGL

AVSDTVNILEKAELVGLISRIRILAYIENVIIQAPFTHGSYCLICSNLLSGWRCNPLAFLIQKILLFPNYLNSGKLNVIS

YDSLETYESKLYDKVELNKNSQNKIIPISKMDPDIIKQAKNILMKKGQESDNEKIFSKLEKLEKRFMTMEVILNSIKKKI

ENNNCNILEHEN

>AmGB16264

MNPSESQQNLLADDARAPSVQSLNAPTDYALGPVEKHFLLSAERGDCATVKRLLLENKDHPEILNINCVDPLDRSALIAA

IENENIELIKLLLELGIQVKSWEAVDKSSSNFTPDITPLILAAHKNNYEILKILLDRGATLPTPHDARCGCDECVTSSEQ

DSLRHSQARINAYRALTSPSLIALSSRDPLLTAFELSWELRRLSRMEQEFRFEYEEMRELTQNFATSLLDHARTSLELEI

MLNYNPYGDNWEPGERQTLDRLKLAIKYKQKQFVAHPNVQQLLAAIWYDGLPGFRRKNMVGQFIDVGKLAAMFPVYSSIY

MLSPTSPMGLFMKKPFVKFICHSSSYAFFLSLILGEIRSLWSDGLMEYISDLWNIVDFVQNMFYVIWIMLRITAWIIVQK

EYRSGLDPWYPRDQWHAFDPMLLSEGAFAAGMIFSFLKLVHIFSVNPHLGPLQISLGRMIIDIIKFFFIYTLVLFAFGCG

MNQLMWYYADLEKQKCYNIKDFPDLPDFDNQEKACSIWRRFANLFETSQSLFWASFGMVDLMSFDLTGIKSFTRFWALLM

FGSYSVINVIVLLNMLIAMMSNSYQIISERSDTEWKFARSHLWMSYFEDGDTVPPPFNMIPTSKTFNKVLSCGKAGRQTR

SLIKKSREKAMARHDTVMRLLIRRYVTAEQRKRDQFGITEDDVMEIRQDISTLRYELIDILRQNGMRTPMLEKQDAAISG

KKGRVMERRLQKDFQIGIVEGIVNAVIQSEKEPKDVFSQIAKAIGRKSSGSKKKDWNAVVRQNTIAKDPIGSSNEAFEKQ

TRRSIRRLGHQPNSDLTSLDPNRLVDYNPNLMEVSPTTRIAYAKFMMRRPKPAAGEEEGKYIFVTFDIFAIHI

>AmGB11954

MSCSRVPENYVANLDAVRVCAKPIRGKDWMKRERMSVSESAHKDEEHGAGGARSPGESGLGEEIGAPDEDCAHLALRMDT

NTSRGWNSGGRAFLRGIGQRVSFDPEAPPPPSQPARKIDEKVKRHSIHGMIEEENVVRPHQEMASLSYQEKKYLLAVERG

DVASVRRMLQSAQETEMNINCVDPLGRSALLMAIDNENLEMVELLIEHKVDTKDALLHAISEEFVEAVEVLLEHEESLHR

NGEPHEQSECRNESWEALPSDTATFTPDITPLILAAHRDNYEIIKILLDRGSTLPMPHDVRCGCDECVTSRREDSLRHSR

SRINAYRALASPSLIALSSKDPILTAFELSWELRRLSFLEHEFKCEYQELRRQCQDFATALLDHTRSSYELEVLLNHDPT

GPAFEHGERMHLNRLKLAIKLRQKKFVAHPNVQQLLASIWYEGLPGFRRKNMVLQALEIVKIGVLFPFFSVAYIIAPHCV

VGQTMRKPFIKFICHSASYFTFLFMLILASQRIESVIGNWMGRDVVEQDTVPTKRGAAPTIIEWFILAWVSGLIWSEVKQ

LWDVGLEEYVNDMWNVIDFVTNSLYVATAALRVVAYYRVKWEIEKSGSEVELQREQWDTWDPMLISEGLFSAANIFSSLK

LVYIFSVNPHLGPLQVSLSRMVMDIMKFFFLYVLVLFAFSCGLNQLLWYYADMEKKRCPTAMSYAPNASVTTDSNACIVW

RRFANLFETTQTLFWAVFGLVDLESFELDGIKAFTRFWGMLMFGTYSVINIVVLLNLLIAMMNHSYQLISERADIEWKFA

RSKLWISYFEEGGTVPPPFNIIPTPKSVWYMGQWLYRKLCGHSRAAKKEHMRTIRRKVKQASERDFRYQSIMRNLVRRYV

TVEQRKAESEGVTEDDVNEIKQDISALRCELIEILKNSGMNTSTASGTGTGAGGKKNRQKERRLMKGFNIAPQPSGSGSL

PPVDEFAASLQQAQQENSHELFGSTLSGIFGPGTTPKKSPHHTSTNSVPGLGTSRQSRRIRGSSKKKRWENLIEAAKVRG

KVSRLIGRSRSEDSVYSPASEDGGSRSEGSTDSKSSLEQGANDGQPTHHPHHAHHSHHHEGGHHVFPHGLGALVALRKKR

KTFSDSRSSTSGMRSGSGTNPIYPLATALVSKVSRKQQLQRASSVPTRGPELGQQPIPPRRHEGTQSQQPSIDTPEGASA

NEQPDRFVCVVCEVVPAAPLTPSTTEESVAASTSLPATMKRNGSATQLQRLPGIEPISGHDVSGGWL

>AmGB15944

MGCEKTEKEVEAPPTEENAYLIHFPKSLNIEEKKYLLAVERGDLVNVKRFIQYANKSHGKSMDINCVDSLGRGALCLSID

SENLEMVKLLVVMGIETKDALLRAIDQEFVEAVEFLLEHEELLTANSMENNSEKEVIHSWQKIDPASARYPPEITPLILA

AQRNNYEILKLLLDRGATLPMPHDIRCGCADCLRSTIGDPLRISSTRVSEYKALASPSLIALSSPDPLLTAFNELGTSRI

DDCRTGKFAVDLLQQVRTTTELHTILNYDPEDDANLPPKQLARLELAIQYKQKTFVAHSHVQQLLAAIWYDGLPGFRRMS

TGKRCGILAKTALMFPFYCMMYLIAPESKIGQLMRKPFVKFLVHASSYLFFLFILMLVSQRAEIEIVRLFGSDETIKNMD

IELTKQRGAAPSLLEFIVILYVIGFIWQEMREAYIDGLKGYLRDMWNFIDFTRNFLYIATAILRLVAYLQQKAEIRDNPS

AALIPRENWSDFDPQLIAEGLFAAANINSALKLIHLFSINPHLGPLQISLGRMVIDIVKFFFIYTLVLFAFACGLNQLLW

YFAELERQKCYVDFADPSWDPASDNCLRWRRFSNLFESCQSLFWASFGGIGIESFELTGIKSYTRFWGLLMFGSYSVINV

IVLLNLLIAMMSNSYAVIEERADKEWKFARTKLWMSYFEDSGTLPPPFNIIPPPKLFLRLCGLRKITGKMSDCKRARDSK

YGAVMKALIWRYIVNTHSEHEMNPVTEDDVHELKSDLSSWRCELLEILRRNGMDIASADTKERTILGKKMKVWERRLMKD

FQVTVPLSIDEDQMESFQQTNEEEDNIAKWKRIAKLAVLQSANHRWNQVLDSAVKSSQIGKSTSKSSIQNQISLKKAMEE

AQKLNNKSTLPPVSLASKPVMSMDYIDEKSAKIVPPQQLKRKSLNSSTTYQQQPEMLSSHSTNTFKYTASSPIKKSGSGT

ESLSAHMDEIPKTLKSRLKPTLSPKKNLNMPEDKEKLVNVMPRSPRGPSKSPRKGGWL

>AmPyr

MCGKSPLYYAVLSNAVDCVKALLEAGASPNNPQVYTETPLHVAAGLGSIACTKLLLNYGADVRVQFGSMRSTPLHLAAEE

GSVECTKLLLDAGATCEAKNARGQAPMHLAVLSQSMETLEVLINIGANVNIEDNDNRTPLHAAVAKTTRGIDLVKILLQA

GALINKADKFGYTPLHIAALNESSSIVIMLLSKGADVTARTKGGISALSFIVRRTPDVLPRFISRLDQAISLHDHELGDI

DCELKLDFRPLVSGGRGETDLMLCLVEVGQRHVLKHPLCESFLYLKWLRIRKFFLLNLIFHSIFVVFLTAYITITYLSNI

ENTEKFRKLFFWLVLIFIVTLASKEFFQMVHGMYSYIKRWENWLQWSVVLLSSIVLIIPIHKWQYHIAALDILLIWIELM

TVIGRFPIFGLYIQMFTQVSINFFKFLGAYICLIVGFSLGFSVLHKNYDSFNNPLIALLKTIVMMSGELEFEDIFFSSKS

PILYSGTAHLMFLSFVILVTVILANLMMGLAVSDIQELRRCAGLDRLVRRAELVAHLEHMLFSKLLDYAPNTIMKACRQG

ALLLHPPHYCTIHIRPNDPREKRLPQELMKAIYRLVIEKKNRNMKKKMPIYNTYNIEMDIPKLNRLYSTTSSDNNHQQLN

EIIDELQRCFYNISTHLDELINKVESIVRDCNNSNT

>AmHsTRPA

MDDKQIHENSTDIPKASLQLPTITVGNERPRRYSLTSLKDRRLPFENENDCGRAVEGEVEAHENVDKTWQMKMKPLANHE

KRTRHYSLTRSKNRRSCLYTDEDRNFLTKSVVNETVNDKTTQTLRVPLSREGRTIHCSLSNLNDPSNDLLSTRLTSTFRS

VRYRQSSNSEKIAIDDIPPADSSNDHVEIDAGTPPPVDESLFYDNLDVFRQAQINSNDLRSIMWSIVTTAEMKILLQLEK

CNALPEIPAGERIRNIAYMWCCYRNLAHLLPKLEQSGVKLDYVEPSTGMNAILAASLSGSVACIEHLIKRGVDINYRNPI

NHYTPLHFAILGNSPDTARILLDNGAKPSTYLYQEVAEPVLHCAIRAGAVEIVKLLLERGASVVEKNHMGETPLHVACFV

QSIKCVELLLDSPGTNVNAVDRAHRTPLHFAVMTTYSSAKLVELLLKHGALVNAADKTGFTPLHVAALNEQSHCVDVLIW

AGADVSATTSAGLSALNIILRKIPDSLQVFRQRLDASITLRRPVPHNREFEMRLHFDLLFPSGNQCETSFINTFVQERRK

DLLSHPLVMAFLHLKWEKIRKFYLLRILVYAMTVICMTTYVLTALAYKCYNHDEANSSKICSSKRISGFLFRRPVIEIQW

YLLLIFTCISIPRKIFGFMVYTSAKQYFSNIDNVLDGVVIISVFVTSFVYTGRTYDWQNYVGAFAILCAWTNLMLMVGQL

PAFGTYVAMFTHIQFEFAKLLLAYSGLLIGFTISFCVIFVGEPSFGNPFTGLIKVLAMMAGELDFEGLITQIDQGLESDG

PFVIYHPLSVCSQILFTLFIVFVTVILMNLLVGIAVHDIQGLRNHAGLTKLVRQTKLILFTEMVLHNSSIPYAFRKWMSD

HKINVENRRRVLVVKPLNPLEKRLPKDILKAAYEIAQKNIPFMNDENINLSDHVIKMRQHSEEYFDSNLQLVIENLANKL

QSYEDMIKSLKEQLLDTNKMLESVVKNLAKEKNN

>AmTRPA5

msageddrsr liasiengdv etairifter fdkkllkpvg vlyvtavqla awqgeielld

lfyrsgadin atdkigrcal fhaahrgnye vvnwllehga ytenrvgiea cykkipgsss

lnigrnlptp ecwgrtamhq avknnhpevv rmlvnagada nvrddrgitp lllagskver

edsneiskyn ciieilvsak vcinvvhpdt gttalhsavl lgsllatrrl lnggacplyq

ckstgstplh laanagnpei lsillesiss hqidirdnid rtplhrasyq gnskcveili

dhggnlaakt gtgvtvidai fahiptpvll lndildscvk mncngnsqii vdfnvlapkg

elqmgvvtsl iaaassveql tilqhplvet flwlkwsklr iffftlvfih allvlslsgy

sitiqyhqtd ctplrrilas cstiillhyt iqvlmvpkyy lrqletwlsf acsvisftis

mnedrgteit tspggeglna rhppqwvlht islailvawm qmmlligrlp mcgnyalmfs

tvlknilkvl lafvclivgf afsfavlfhg ndqfrnswra vvktvvmmmg eyeygdlfsd

ekngssflta tsrvvflafv mlasivlmnl miglavndiq glekeghirq llkqaefvgh

lerltshrif rgnwlhprla rlldsrrgip tkitfgyhkc ylhesfpgip srlrealfll

atensrrfes grennndkve lrtlleqvll qlrmshtygi gykfpkhigm ksqidrrksa

i

>AmTRPML

MSEPRKRMVKDESRGNILRNHSWSNGPDSEDEQAGENELLNECSVSPSHHRSTITDQPICNMSLFTEEKMRRKLKFFFMN

PIEKWQAKRRFPYKFIVQVIKILLVTIQLCLFAHNNYIHVNYTWDNRIAFSHLFLRGWDSSLEVPVYPPVTGPLAIYKRD

DFYNTIDYALDGYYNVSNAVGSYSYIAENNSVGPVILCLYQYKEGIIFGFNESYVFDKEIIETCINITDKTYTESSKSKL

LLFKQNIKVNFSALVRAHLMFALKTVNLKAAGPMTPPDCYQFNIKINFDNRDFDGQMLLSLDAEPKRLQCKGDTRYVTDN

RIESALRTLLNLFVILICTVSLILCSRAIYRAQLLKFETMNFFKKAYGKILSLEGRLEFLNLWYVMIIVNDLLIIMGSAI

KEQIERKQFGSDHWNICSIFLGTGNLLVWFGVLRYLGFFKTYNVVILTLKKAAPKVARFLICAILIYAGFTFCGWLILGP

YHMKFRSLATTSECLFALINGDDMFATFSATSFSKSPMLWWYSRMYLYTFISLYIYVVLSLFISVIMDAYDTIKIYYRDG

FPKNDLQTFIAACTDEASSGLYKDDSEDSDLSEFIDRFCCCRKRPFYGSFSESSTKFSTKSEQTCGGAICI

>NvTRPM

MAIGSVICGANTPKTKRKKTKTTERSWIEATFQKRECTKFIPSAKDEHRCCCGHSYTHHCGTGADVQSFASSAAAEAERD

EWSPGKNTKHCPTDAYGTIEFQGLPHPTKAQYVRLAHDTKPEPIVQLLCREWNLGLPKLLITVHGGRSNFELQPTLKKVL

RKGLLKAAKTTGAWIFTGGTNTGVTRQVGDALLLERSQRQGRVVSIGIAPWGILEKSHELIGRGREVPYDSLASPWSKFA

VLNNRHAYFLLVDNGTGGRYGAEIVLRRKLEKYISNLKLQPYTHSSIPVVALVIEGGTNTIRSVLEYVTDEPPVPVVVCD

GSGRAADLIAFMHKYASENESEEEEGPLESMRDHLLDTIKRTFKVSPEQANQLYSELLQCVRRKHLITVFRISQDQPQEL

DQTILTALFKSQQLSPAEQLSLALIWNRVDIARSEIFVYGQKWPPGALEQSMMQALQHDRIDFVKLLLENGVSMRKFLSI

PRLEELYNTKEGPSNTLGYILRDVRPHIPRGYMYTLHDIGLVINKLMGGAYRSQYTRKEFRFLYTRVMKRSGGYQQHHAH

RNSCAIALNSRSFSGSGGKSTMSLLAETIPATQETALFDYPYNELLIWSVLTKRQHMALLMWQHGEEALAKALVALKLYK

AMAHEAAEDDLETEVYDELKGYGKEFENIALELLDYCYRQDDDQTQQLLTSELKNWSGQTCLSLAVTANHRALLAHPCSQ

IILADLWMGGLRTRKSTNFKVILGLFCPVYITQLEFKSREELQLMPQTAEEHLIALEDENEDSESEHGTPTGPDVEALIG

NEHGLGSAAPKETIVHENGKVLTDNDEGNHRAYGILSEYYDNKNSRPLRLKKKLYEFYTAPITKFWANAIAYVIFLLLFS

YSILVRMGDVPSWAEIYAIAYICTMGCEKVREIATSEPVALSHKFSVWAWNMWNPCDAAAIIFFIIGLVLRLRPSTFDVG

RTIYCVDCMYWYLRMLNILGVNKYLGPLVTMMGKMVKNMIYFVVLLLVVLMSFGVARQAILNPNAEPNWRIIRDVFMEPY

FMLYGEVYADQIDPDCDNNPGMEPCLPGRWITPAAMSVYLLVANILLINLLIAVFNNIFIEVNAVAHQVWMFQRFTVVME

YEQKPVLPPPLIVVSHLYLIIRYILRYVTQGRLQSGETSDNGLKLFLEADDKERLYDFEEDCVEGYFREQELKLQMSTEE

RVKVTTERVENMHQKIEDIDKKESNQSSSLQTVEFRIRKLEELNEQTLAHLGVIHRFMATYMPVGGGGAGGGGGPGNENS

SSTEALRHLDVELIRPRRVSERSEAGMSESDSHAQLPCIPLRRKKLIRAMTDAAYMNMAAGGLVGQQQHREQLQEQMEQD

EATDLNRAVEAMVGDNLSRNGSSVSGEIATLQDESKENTSHDESDLSKAVGELKDLKAEKSDDRDTSGDVTATTDSRQDS

VEKSSRQNSRTRSESLADDAAAAAAIAASVSGVVVPSASFQRGVTWAEPRVAVIQSSGSSNSRSLLLAMRAEYTSITDEL

ESYCGLLSPPRTPPVSPPPCRVRSVSERSNQNPVEIAWQIENEHLRDAEECDYQQMEDLIQRRYHISDEEDSSHDDPAVV

NTTSFFISSEHHPRLRRASAIDEDTTTRRRCHRSPPSINVTREIEQTLARPAATRDSAESDPNDKGMSGVAAPASETMC

>NvWtrw

MAEPSSEEPSPLDKVFPASPNSAVHNNNTTTVNHNNTRNRRVQLERSSSSIQFPASAMALAHKSKTSSNLLTGRCLSSSN

CSSTTRRVSLPSTEDPTWVGEDRCMEYGTPPPIEEPHCYSMCLSESSFSEESSCAVQINRDYLKGLLVEHTRTLGCRLLV

VEELEAGKLDLASADKYLEGCKKPQELNALLLYASFLGFGELIPIVHNHGADLNHSEPELGMTALHLCAFSNCLSGVRYL

LGHNVHVGSKKAHTPYHFAAFGNAIDVAKFFAQLGWSQESPLGEETVLHAAARNNALDVLRLLAPKNPTLNRLDSAGYAA

IHYAAERGDSGCLKVLLAAGCLVNVSTRKGETALHLSAEAGCAESLELLLAKGANPAVRNRRGQTALHLAARTHSLECVE

ALLQHSRSEPNAEDHDGRTPLHVALGRSLLAYDVTEMLISWKAQVNKPDKYGYTPLHVAALNELSQCVDILIQHGADLSA

KTKGGTSALSIILRKTPSSLNCFKQRLDASISMHQHGSAAGEVELRLDFHPLLQHQQQGEIGYLGTFVKEGYKEILEHPL

CQSFLHLKWQKIRKYYVGRLLFYLLYVLVLTAWVITALAHNCYNESHGQLDNSSRPLCANSTGLNGFLYRHPVVLEVEWY

ALVVLTVLEGLRKLMGILTYSTVRQFFTQAENMVEWCVIVSVFATSFVYTGRTYTWQSHVGAFAVLCGWSNLMLMIGQLP

MFGAYVAMFTSVQAQVFKLLLAYACLLVGFTASFCVIFPRSKSFSSPHTGLIKVLVMMTGELDFEELFFPGEEEGGRPVD

VGGTSWILLQVSAQLSFVLFLLFVTIVLMNLLVGIAVHDIKGLQKTAGLAKLVRQTKLICDLETALFLGLLPQCLMKVLR

WTALVLPSPLKAVLTVRPLNPREKRLPRDVLAGAYKVARERKGNLLNSTLSSKRSNNSTIYGYQKFDLNASVRRRTNMNE

EPATTLEEAARLKEEVAELRKICESNNLLIQELVKSFTFGSIASDYRKRRKVIYMILVIMSRKNKRNLRETSV

>NvIav

MGGACSCQGRDSQVNAGSILDRVISQASDEDQCLLYRLANYKKGGELIEAYNQGGQAEVERLIREQFGILMYADGKGQTI

NRAEYLRWKFRDLDQVILPIEASLSRFDPLAQWSDHEACWQMQYRGSLGETLLHVLIICDTRTHTRIARTLLKCFPRLAI

DVVEGEEYLGASALHLAIAYANNELVQDLVEAGAIVSQRAIGSFFLPRDQQRPRPAKSTDYEGLAYLGEYPLAWAACCAN

ESVYNLLLDSGAHPDEQDTFGNSILHMVVVCDKLVRFGYALRHPKLPASNGIANAAGLTPLTLACQLGRAEVFREMLELS

AREFWRYSNITCSAYPLNALDTLLPDGRTSXNSALFIILNGTKEEHLDMLDGGIIQRLLEEKWKTFARLQFLKRLVILVF

HLVSLSLAVYFRPADTDAELAQWPEEITDVVRVIAECVTVLGVLGYILLQLGGEIVNIGFFSFFKQLSHEPAKFIFLISN

LLILACIPCRLSGDRHTEDAILVVAVPGSWFLLMFFAGAVRLTGPFVTMVYSMILGDMRTFGIIYMIVICGFTQAFYFLY

KGYPGVKTTLFHSYPSTWMALFQITLGDYSYSDLSMTTYPNLSKAVFTIFMVLVPILLLNMLIAMMGNTYAHVIEQSEKE

WMKQWAKIVVSLERAVSQDDAHNYLQEYSIKLGPGDDPNDPASEQRGVMVIKSKSKTRARQRKGAVANWKRVGKVTIAEL

KKRGMTGEELRRIMWGRASFSTPVRSSPILMEPVSSVPGGFGDALTSALDVMTFAADVPVPIEGSIPKPVAVAPGQQPTT

TLALPNGAAKPQQPMMTTSTTTTTITTTVQKTLVTGTATTPAVGVTKTVAVPAATTVSTTTTRASASAVPPTVRPSELGP

ASAPCSADPLLELMLATEDPGSSEETLQRLAHSARIVGESATTSAKSDIDSSFLERLALGGLGLAAMPEQPDKPVEPKKK

QYLALKSSLKSDRASLQHPLSHDGQLGTEARLRRIRSASSRLAPTTTTSTSRRKSGQQKQQREARDDESTSSAASLDNIS

GYQPLLNEPDTTADQPATGTEDNKPANGRNLRKRPKTTSNEGKVFCSRRTIVVLNKNKKNKKLETSEANMNARKKETLLA

LHDMTSLISDNWIQINIARFQNNYSHSKVTKNLKKCHLHIESDVLLTSQQCCINISNSKAKMNQALLVVLSFFVVNEDQP

VAGWTWGKSATSSSNNSINDDRARYCRICASHTMCLFPYDDPGPKCAAVENGDLEPEEIEWILQRHNSLRDGIARAWKSQ

YRPLPARDMMQVFWDEELAKIARRWALQCNIHEKDQCRDVEEFSVAQSVSALDLQDAGNRSEMERLKFHLRSWYSQLEPD

FSNSAGVGLATPSVTNVGCGRATYSVAIDDSDVAAAVEVLVCNYGPIDDPETEDDSGCETRSRRYSELCQLTRRE

>NvNan

MGNAESNVTSGVKKQTDAGAIALYKLVDLKGGGLLVDMMKRASQTKQYAEIDHAIRTKVEPFLYNGGKGKWIPIAKLVLL

RNKERGRHKMLPVLKAMEKPEDYDIDKDMANDPEPDENTIGMMIMKILSNKSKYKLVCWTLSDRGAVGETILHLCMLNAT

QLHADLAKRLLRFYPNLINDIYIDDEYYGENVLHIAIVNEDPSMVKFLLDSGANVNERCCGNFMCPEDQKASRNDSVEHE

WVCVCSETNYDGYVYWGEYPLSFAACLDQEECYRLILAKGADPDSQDTNGNTVLHMLVIYEKLETFDMAYEVGSSLSIRN

VLQLTPLTLAAKLARVEMFFHILNIEREIYWQIGSITCAAYPLSQIDTIDVDTGKISNNSALNLVVFGDKEEHLKLLEGV

LIDLLNAKWNTFVKSRFYQQFYLFFCYFILSLISCTLRPGPITKTDAPTTTPHPMNDTSLYADVNETSASDPFDLNASDI

VILKKGKKELDDWWDDLTEDCRLMQMNSTSAKIRLTAEIFMEFGAILYICAALREARFLGLNMFIENLMTAPSRVMFLFS

CCILLSFPFLRMSCADEVEDILAVVVMLTTAPYFLFFCRGFKTVGPFVVMIYRMIMGDLLRFVSIYLVFVMGFSQAYYII

FLSFDNPITPEGVDDSKANPLPSAMESIMAMFLMSMTNFGDYYDAFENTEHEMLAKCLFVVYMAIVSVLLVNMLIAMMGN

TYQKIAETRNEWQRQWARIVLVVERGVAPAERLKKLNVYSQPMSDGRKALVLRLNQTDKDKEEMKDILEMKRIHIKSIKR

RKDKLQKAKEDAEKLQQQMADKDNKDKAVIIQ

>NvNompC

MSSSKKGGGKEEGKKRGAGAAVSGDVGGPSSKEDSPAGAGGLAGKDGEPGSAGGSGSGAASNGAPSAEPSQPGSKPGSAG

ATAREAAQRLLGVATRGEWTAADQLLKTLEKAVQSAGDEANLQPLAGLADPQTGMTPLMYAVKDNRTAFLDRMIELGADV

GARNLDNYNALHISAMFSREDVVKLLLSKRGVDPYAPGGPRQQTAVHLVASRQTGTATSILRVLLAAAGRDIRMKVDGKG

KIPLLLAVEAGNQSMCRELLSQQAPDQLKATTPTGDTALHLAARRRDVDMVRILVDYGASVDMQNGSGQTALHIASAEGD

ETLVKYFYGVRASAAITDHLDRTPMHLAAENGHASIIELLADKFKASIFERTKDGSTLMHIASLNGHSECATMLFKKGVY

LHMPNKKGARSIHTAAKYGHVGIISTLLQRGEKVDATTNDNYTALHIAVESAKPAVVETLLGYGAEVHVRGGKLRETPLH

IAARVPDGDRCALMLLKSGAGPNLATDDGQTPVHVAASHGNLATLKLLLEDGGDPMFKSKNGETPLHLACRGCRADVVRH

LIEFVKEKKGVDVATNYVNSLTFEGASALHYAAQIEPTEVVVEGDDRAVVRALLEGGADVSLQTKQAQESAFHYCALAGN

NEVLSEMIGHMSATEVQKALNRQSAVGWTPLLIAAHRGHMDIVKNLLENHARVDVFDLEGRSALHLAAEHGYLEVCDALL

ANKAFINSKSRVGRTALHLAAMNGNTHLVRFLVQDHQAAIDVLTLRKQTPLHLAAGAGQLQVCKLLLDLGASIDATDDQG

QKPIHAAAMNNYAEVAQLFLQKHPSLVMACTKDGNTCAHIAAMQGSVRVIEELMKFDRNGVITARNKLTEATPLQLAAEG

GHAEVVRALVRAGASCAEENRAGFTAVHLAAQHGHGQVLDVMMRSSQSLRISSKKLGVTALHVAAYFGQADTVRELLTHV

PGTVKSEPPTGGSLVGELGNESGMTPLHLAAYSGNENVVRLLLNSAGVQVEAATTENGFNPLHLGLSGGPISIGAAFREI

EFVRAIERSKKGLYCAGIFHSSIQVKRMRLSPRIRKAHRHMYTRAHCVLAFGNKSLESKRVCVRVCITQLDMIFDDEVVF

RSTVFLSQFVYNMMVCSKSHNNKPIEEFVLVSPAPVDTAAKLSNIYMKLSEKEKERAKDLIAAGKQCEAMATELLALAAG

ADSAGRILTSMDRRNVEFLDVLIENEQKEVIAHTVVQRYLQELWRGSLNWNGFRTILLFIVFVICPPVWMVFALPLGHKY

NNVPIIKFMAYLTSHIYLMVFLALVGILPIYPVNREPLTPYWYEWCLLVMLSGLLLFELTNPSDKSGLGWIKLAVLLFGI

CGVFVHLLGQSGLVKKNDWGTLLYIRNQFFALSFLLACVQILDFLSFHHLFGPWAIIIGNLMKDLARFLAVLAIFVFGFS

MHFVALNQAFKEGKQKDTNKRPAFSDGKFEMVAQNLTEWEMIDIPPPQPTPRENYISEISSAKSNTSKLNMKMNPVLAFE

YLFFAVFGQTTHSELKVEQNQPEWTSSLFKLAFGVYMLVSVVVLINLLIAMMSDTYQRIQKRKQKKDRPSLVNMMGLRQG

GRLSARSKAGTKWLSKVKKTSQVAHKDSVTLSVMHLSPLGSQLSFGNATRIESVVDWDVVRRKYRELQGEVSDKSGMDSK

DDSESDSQLMPVDDASPSLVGSQATLIASTS

>NvPain

MDSEDEALEMQLLQEQNADRYTPLMHLLKNNHLATFKSFVRQALNRQPPSIDVNHLLSYPEHRTFLDAAASLNLPNFVAF

LLEVGANPNLINSERNRAPIHFAAEAGHSEALEALLKDRRVNLNLEAGGLTALHYAVKADCGICTRLLLDAGASPNIPND

KGNSPLHYAARAENREAIELLLQKGCYVGHMNSFGSPPLAHMAPGILEPHLDECLTSSNERTEEYEIIMNYQNLVPHNTQ

CGADVSYLERNRRRSSFKNRSSNPCSETEALLFIANNKSLRHLLKHPLLASFLYLKYLRIRHVLYVNFFLYLVYFFMLIS

YIWVITSEAELEAEELRSKNLTSIKPKQDDDVDVAEEENGMFANLENHPIHSALLFAALIYMIVRELLQFISSPLHYIGS

PENWIEITLIGLTGGLLLGGGVKLGSLAALLSTWELVILMSQHPRMSTDIEMFKTVTLNFARFLFLYVFLILAFAFAFFV

LFRDPENENFVQPPLALFKTIIMLTGEFDSSDLPFSHFPVLSRLVFCGFIFLIVIILLNLLNGLAVNDTAEILSQAELVC

LISRARLVAYAERIAVGQPFVPKTRLCCFINYLPCQSLTTGPVRFMARRILLFPRYLPHFTLSVKPLKNYEITLHGRPFT

GSRCSTLKMDPVVAQRAKEILEAKGRVSSEDKILEEIETMRRRFDGIEAMVREIRETVRNNNLNAED

>NvXP_001605329.1

MNASESQQNLLTNEAKPASTSQQWLSPPADYVLGPVEKHFLLSAERGDCATVRRLLEENKDHPEILNIDCVDPLNRSALI

AAIENENIDLILLLLELGISVKDALLHAIKEEYVEAVEILLEHEEKIHQPGQPYSWEAVDRSASNFTPDITPLILAAHKN

NYEILKILLDRGATLPTPHDVRCGCDECVSSSEQDSLRHSQSRINAYRALTSSSLIALSSRDPLLTAFELSWELRRLSRM

EQEFRSEYNEMRENVQVFATSLLDHARTSHELEVMLNYNPSGENWEPGERQTLERLKLAIKYKQKQFVAHPNVQQLLAAI

WYDGLPGFRRLSMMGQLLEVGKLGAQFPVYSTIYMLSPNSPKALFMKKPFVKFICHSSSYAVFLMLLGAASQRIEYLAIE

LFGNAWMRQILAGWKRRERGSIPGFVESGVIIYVFSLVVGEIRSLWADGLLEYVSDLWNIVDFVQNVFYVIWITMRATSW

LVVMREYWAGEDPWYPRDHWDDFEPMLLSEGAFAAGMIFSFLKLVHIFSVNPHLGPLQISLGRMIIDIIKFFFIYTLVLF

AFGCGMNQLLWYYADLEKNKCYHLPNGLPDFDNNEKACATWRRFANLFETSQSLFWASFGMVDLMSFDLTGIKSFTRFWA

LLMFGSYSVINIIVLLNMLIAMMSNSYQIISERADTEWKFARSHLWMSYFEDGDTVPPPFNMIPTAKTFDRIVKCGKSGR

PTRSVIKKSREKAKERHDTVMRLLIRRYVTAEQSKTDDFGITEDDVMEIRQDISTLRYELIDILRTNGMQTPNVDKGDTS

LSGKKGRVMERRLQKDFQIGIVEGIVNSVIQNEKESKDVFSQIAKAIGRKSSGGSKKKDWNAVVRQNTVARDPIGSSNEA

VDKQHRRSLRRHLQHGNSLLESLDPQRLLEYNPNLVEVSATTRVAYAKFMLRRPAAVKEAAEGEEAPADGEGKTSKRSSQ

RVAIIDPAKPGEKPPDRPPPATRALGTLSKKSLIKAASSDKPEAGKSSLEAKPSQEQRGGPSPIPEEPHEESKAPIGSRA

ASLKKQPSAAASEPKKPTADEGPTTKARGRSKATGQIVGGWI

>NvXP_001604587.1

MDFKEDELSEDEIHGPDTQSAGEDEDQFARGSGSPGGGSADEGSAEPSASATAAGVASNQSTVAVRMAENRRPRGFLAGP

ARHPPPPLPLQHTHVNFDPEAPPTPTSNQPLHGGGPGLLHPQAYPQMSLLEKPEIDKKVKRHSIHGMMEDENVIRPHQEM

IRLSTDDRKFLLAVERGDVAGVRKILTENKEKININCVDPLGRSALLMAIDNENLEMVELLINYKVDTKDALLHAISEEF

VEAVERLLDHEEEFHRNGEPHSWEALPPDTATFTPDITPLILSAHRDNFEITKILLDRGSTLPMPHDVRCGCDECVTSRL

EDSLRHSRSRINAYRALASPSLIALSSKDPILTAFELSWELRRLSFLEHEFKCEYQELRRQCQDFATALLDHTRSSYELE

VLLNHDPTGPAFEHGERMHLNRLKLAVKLRQKKFVAHPNVQQLLASIWYEGLPGFRRKNMVLQALEIVRIGILFPFFSVA

YIIAPHSVIGQTMRKPFIKFICHSASYFTFLFMLILASQRIESVIGVWTGRDISEHEIEPTKRGAAPTLVEWFILAWVSG

LIWSEVKQLWDVGLEEYVNDMWNVIDFVTNSLYVATVALRVVAYYRVQKENEGSDIIVDLQREQWDTWDPMLISEGLFSA

ANIFSSLKLVYIFSVNPHLGPLQVSLSRMVMDIMKFFFLYVLVLFAFSCGLNQLLWYYADMEKKRCPTAMPHLPPNANIT

QDPNACIVWRRFANLFETTQTLFWAVFGLVDLESFELDGIKVFTRFWGMLMFGTYSVINIVVLLNLLIAMMNHSYQLISE

RADIEWKFARSKLWISYFEEGGTVPPPFNIIPTPKSIWYITQWIYRKLCGHSRAAKKEHMRTIRASERDDRYRAIMKNLV

RRYVTVEQRKTENEGVTEDDVNEIKQDISAFRCELINILQKSGMATEHTSGTGTGVGGKKNRQKERRLMKGLGGLDGPLG

PQPSGSGPLPPVAEATASLRDHQHQTSGHHPHDIFSSTLSGIFRPGTTPKRNPHHTSTNSVPGLASCQRQSRDSRGSSRK

RRWGTLIEAAKAGRVSRLIGRSRSQDSVYSPASEDGESRSDGSSDSKSSLDVPSQDSHPHHPHHQSHHPHSSHHHQQHQH

HHQHHYALGPALAALRRKRKKFSASRASTPAMTMSSGNNSACTSSTLESVMQPIASALVSRVSKKQLQRASSVPTRSPEL

ALHQVVAPPRRHETTQSQQPSLDDTPSSNNGSKEQGGVTASAAAVAAMTSSAVDLTPSTTEESVLSASTAIVTAVKRNGS

AGQQQQQQQQQQKLPPGIEPISGHDGTASTGWL

>NvXP_001604491.1

MVSSASEKEMEAAPEKKRGLSHYAFHLPRPLNIEEKKYLLSVERGDLPNVRRILQAARKPINKSIDVNCVDSLGRGALTL

AIEAENLEMLELLIVMGVETKDALLHAINQEFVEAVELLLEHEEPAGKAEIVHSWQKVDPSLARYAPDMTPLILAAQKNN

YEILKLLLDRGATLPMPHDIKCSCDDCVRAATGDPLRLSSTRIAEYRALASPSLIALSSPDPLMTAFQLSWELRGLALAE

PESCAEYLRLRKQVEKFAVDLLQQTRSSSELNTILNYDPDDHSAEPTKQLARLEQAIRYRQKKFVAHPHVQQLLAAIWYE

GVPGFRRMGAIQRAWIIVKTALLFPFYCTIYFFTPNTSTGHTMRRPFMKFLVHASSYLFFLLVLILVSQRFEEELLSWFG

TDYDRQQLEEGFSKQRGLGPSFLECIVVIYVLGFILEETREVYVDGLRAYLRDLWNFIDFTRNALYVATVILRVAAYIQQ

SAEINQDSAAATIPRERWPDFDPQLIAEGLFAAANIFSALKLVHLFSINPHLGPLQISLGRMVIDIVKFFFIYTLVLFAF

ACGLNQLLWYFAELERKKCYSDIDEPTWDSASDSCIRWRRFSSLFETCQSLFWASFGGVGIDSFELTGIKSYTRFWGLLM

FGSYSVINVIVLLNLLIAMMSNSYAIIEEHADTEWKFARTKLWMSYFEEGNTLPPPFNILPHPKLLMRCCGVQRKSMSKR

SSIRRRAREQLSRYGSVMKALIWRYVVNAHSEHEMEPVTEDDIHELKSDFSSWRCELLEILKRNGMDTGGADTSNSTVLG

KKMRVWERRLMKDFQVASLADDPIEEMQHLENPPENEEIAAKWRRIARLAVLKSAEHRWTQVIDNTVTKSQIGRSNNRVS

LRNQANLKRVMEEARRLSLTSPPPPLTPIKLPDTINPTNILRVLKDDDDFNHFPMDVSPAISMTMAMTQEPASLTDNVRT

KNDSSALNYVNERYQGRSASPRPLAPIRTSPPRVVKRKPQAPPPGHALPRSPASNILSVDNLVVPGDGSSSNVVRPRPKS

PRRVPEVAISPVTPEQKASNCGRGFVKIEPRSPRVAAANKSPRRNGGWL

>NvPyr

MSSSRGKAGFLDKLAGRSKSGGRAGNDETKDQPPTIFIDCEDEEQIWMDNMDSDSISSEGSSGSAVCLRHRPSSHQQLWD

KEEVILSLTNLPAGEAALAVLPSLCGEPLDQVADEIGDKTVSQSADLSALIPRFANGCIRKASLIAENESALSSTPADLI

ANWPNTCLLVACWIGHSELAKLLLDKGAQVSYRDADGRTALHLAASIGSVKITELLLKHGADPCEWDFNRKCTALHCAAA

AGDVDTVQCLIRAGADVNAGLSGKSPLHYAVQNNAESCVEALLQAGASPNNPQVYTETPLHVAAGLGSEECMKLLLSHGA

DVRVQFGAARSTPLHLAAEEGSPECTRLLLEAGALPDANNSRGQTALHLAALAQSSETLDILIAGGADVNAEDDNGRSPL

HAAVAKAVRGSELVRALIQAGAVVNRPDKFGYTALHIAALNESSPIVILLLTKGADVTARTKGGVSALSFIIRRTPDVLP

RFVGRLDQAISLHDHELGDVDCELRLDFRPLVPGGRGESDLMLCLVDVGQRHVLKHPLCESFLHLKWLKIRKFFLFSLIF

HSIFVAIFTGYIACTFLWQLPKLGGVLFWFVLLFTVLLASKEIFQVAHGIWVYAKGWENALQWSVILTSGMVLIKPVADW

QHHVAAVGILLVWVELMMIVGRFPTFGLYVQMFTQVAINFFKFLGAYLCLIVGFSLGFSVMHKNYKSFQNPLVGLLKTII

MLSGELEFEDVFFDEAAPVLYAGTSHLMWLAFVILVTVILANLMMGLAVSDIQELRQCAGLDRLIRRAELVAHLESLLFS

KLLDYAPRRIVKACRSSALLLHLPHHCAIHIRPNDPREKRLPRELIKSIYRLVSERKTKSKSCGNNASIRSLNGNGYSSH

RLSRLYSNSSFSEFNRQQLSDLAMELKKCSLTIGARLNALTSKVEAIAKEIDVATQLQE

>NvHsTRPA

MSRSWKLDEVVEEDPSDRVDMSDLNRAPRSPRSECRKPRHYSLGCIYERQRRTSRDLLPGRLASLYKSRQGMRPSICPDK

VVVTEPQDSAEHVEIDAGAPPPVDDSLFFDREFEALKHVKIDGTSLKTAMWAAVNVVEMKDLLEMEKGNIEKGPDGKLLP

NADQRFKNVMFLWACFRGLAHLLPKLEECGADRDYVEPCTGMNPILVASLTSSIACLEYLIEKGADVNYANTALFYTPLH

FAAFGNSSEAAELLIKHGAKLNSSNCQDVEPVLHCAVRARAEKVVKLLLEKAASVAQKNSTGETPLHVACFVQSIGCTEL

LLCSPGTDPNAVDQNHRTPLHYAVMNTCSAPELVELLLKHGAAVNVKDKQELTPLHIASLNEQSQCVDALIWAGADVSAT

TKTGLTALNIILRKIPESLQVFRQRLDASIRLKRPVPHNREFEMRLHFDLLFPSNNQCETSFINTFVQEHQKDLLSHPLV

MAFLHLKWEKIRKFYLMRIFLYALTVIVMTTYVLTALAYRCYNLSESKSSKICNNNRASGFLFRRHVIEIEWYVSLILTC

VTIPRKIFGFMVYKSARQYFMNIDNVLDAIVIVSVFVTSFIYTGRTYDWQNYVGAFAILCAWTNLMLMVGQLPAFGTYVA

MFTHIQFEFAKLLLAYSGLLIGFTISFCVIFVGEPSFGNPFTGLIKVLAMMAGELDFEGLINQDDILHDGSFVLYHPLSV

CSQILFTLFIVFVTVILMNLLVGIAVHDIQGLRNHAGLTKLVRQTKLILFTEMVLYNGRIPYTFKKWMSDHKIDVDNRKR

VLVVKPLNPLEKRLPKDIMKAAYEIAQRNAPLVDEDDVSLDEHVTWMKQQSEEGSDYLLHSTIDQLSTQMKSAEDDIKVI

KEQLLDTNKMLKNLVARRVQPR

>NvTRPML

MARSISARPPSARIDLVEKFVWAPAPFQPPSTGFGSRLTTTPVPTTYMEERMRRRLRFFFMNPIEKWQAKSRFPYKFVVQ

VIKIVLVTVQLCLFAHNNYTHVNYTWDNRITFSHLFLRGWDATQEVPAYPPATGPLALYKQDEFYETIDYALKGYYNIGN

AIGSYSYTAEDNSVGVVVLCLHHYEEGIIFAYNESYIFNSKIVEKCINITKQEEETVTSKLLMQQNKIPVNFSALVKAEL

KFDVKTVNLKAAGPITPPDCYRFRIKILMDNKDFDGQMLLSLDAEPVRLVCKDTQYITDNQIESALRTLLNLLVIFICSL

SLGLCSRAIYRAQLLKFETMNFFKKVYGTSLSLEGRLEFLNLWYVMIIINDLLIIMGSAIKQQIERKQYGNDHWNLCSIF

LGTGNLLVWFGVLRYLGFFKTYNVVILTLKKAAPKVARFLICAILIYAGFTFCGWLVLGPYHMKFRSLATTSECLFALIN

GDDMFATFSITSFKSPMLWWYSRIYLYTFISLYIYVVLSLFISVIMDAYDTIKVYYRDGFPKNDLQSFVSVCTDEASSGV

YRDEADDEPILGGLFGRICCCSQRKNKPHSDFSPLTNSNSTSSTIDSVQVCPSAICI
